# Supplementary material for: Cochrane systematic review and meta-analysis of botulinum toxin for the prevention of migraine
Source: BMJ Open. 2019 Jul 16;9(7):e027953. doi: 10.1136/bmjopen-2018-027953 (PMC6661560; doi:10.1136/bmjopen-2018-027953)
Supplement: Supplementary file 1 [file bmjopen-2018-027953supp001.pdf]

MEDLINE (via OVID)

#1 Exp headache disorders/

#2 headache/

#3 (headache* or migrain* or cephalgi* or cephalalgi* or hemicrani*).mp.

#4 or/1-3

#5 exp botulinum toxins/

#6 (botulin* adj toxin*).tw

#7 (botulinum* or oculinu* or boto* or onabotulinum*).tw.

#8 exp botulinum toxin type A/

#9 Exp clostridium botulinum/

#10 clostridium botulin*.tw.

#11 or/5-10

12 randomized controlled trial.pt.

13 controlled clinical trial.pt.

14 randomized.ab.

15 placebo.ab.

16 drug therapy.fs.

17 randomly.ab.

18 trial.ab.

19 groups.ab.

20 12 or 13 or 14 or 15 or 16 or 17 or 18 or 19

21 exp animals/ not humans.sh.

22 20 not 21

23 4 and 11 and 22
