# Supplementary material for: Cochrane systematic review and meta-analysis of botulinum toxin for the prevention of migraine
Source: BMJ Open. 2019 Jul 16;9(7):e027953. doi: 10.1136/bmjopen-2018-027953 (PMC6661560; doi:10.1136/bmjopen-2018-027953)
Supplement: Supplementary file 3 [file bmjopen-2018-027953supp003.pdf]

Table 1: Characteristics of botulinum toxin vs. placebo studies

Trial (Reference)	Total N randomised	Comparison	No of injection sites (Fixed or FTP)	Location of injection sites	No. treatment cycles	Mean Age (SD)	EM/CM	Ratio of MOH/No MOH
Allergan 2015 (40)	52	155 U Botox® vs Placebo	nr Fixed	Head and neck; no. sites/units per region nr	2	42.8(12.2)	All CM	nr
Anand 2006 (20)	32	50 U Botox® vs Placebo	10 Fixed	3 pericranial muscle regions; no. sites/units per region nr	1	nr	All EM	0/32
Aurora 2010 (PREEMPT 1) (6)	679	Botox® 155-195 U vs Placebo	31 Fixed + 8 OFTP	Seven specific head/neck muscle areas; no. sites/units per region nr	2	41.6	All CM	462/217
Aurora 2007 (21)	369	Pooled Botox® vs Placebo from: Placebo responders(PR) Botox® vs PR Placebo vs Placebo non-responders (PNR) Botox vs PNR	23-58 FTP	Frontal/glabellar, 25-40 U; occipitalis, fixed, 20 U; temporalis, 20-50 U; masseter optional, 0-50 U; Trapezius, 20-60 U; Semispinalis, 10-20 U; Splenius capitis, 10-20 U; no. sites per region nr	3	45	All EM	0/369
Barrientos 2003 (22)	30	Botox® 50 U vs placebo	15 Fixed	Temporalis, 10 U, 2 sites; frontalis, 10 U, 4 sites; glabellar, 8 U, 4 sites; procerus, 2 U, 1 site; trapezius, 10 U, 2 sites; splenius capitis, 10 U, 2 sites.	1	41.1	All EM	0/30
Blumenkron 2006 (23)	30	Botox® 100 U vs placebo	25 Fixed	Front points 10 U, 4 sites, procerus muscle 10 U, 1 site, corrugator muscles 10 U, 2 sites, temporalis muscles 30 U, 6 sites, trapezius muscle 50 U, 10 sites	1	nr	nr	nr
Cady 2008 (24)	61	Botox® 139 U vs placebo	17 Fixed	Corrugator, 12 U, 2 sites; splenius capitis, 20 U, 2 sites; trapezius, 40 U, 4 sites; temporalis, 40 U, 4 sites; procerus, 3 U, 1 site; frontalis, 24 U, 4 sites	1	42.1(11.5)	nr	nr
Cady 2014 (36)	20	Botox® 155 U vs placebo (cross-over design)	31 Fixed + nr OFTP	PREEMPT paradigm + optional follow the pain in occipitalis, temporalis, and trapezius; no. sites/units per region for OFTP nr	1	48.5(12.9)	All CM	nr
Chankrachang 2011 (25)	128	DYSPORT® 120 U vs DYSPORT® 240 U vs placebo	6 Fixed	Frontal, 2 sites; temporal, 2 sites; occipital, 2 sites; units per region nr	1	38.6(9.9)	All CM	nr
Diener 2010 (PREEMPT 2) (7)	705	Botox® 155-195 U vs Placebo	31 Fixed + 8 OFTP	Seven specific head/neck muscle areas; no. sites/units per region nr	2	40.9	All CM	444/261
Elkind I 2006 (26)	418	50 U Botox® vs 25 U Botox® vs 7.5 U Botox® vs Placebo	11 Fixed	Frontal, 4 sites; temporal, 2 sites; glabellar, 5 sites; units per region nr	1	44.1	409 EM/9 CM	nr
Freitag 2008 (27)	41	Botox® 100 U vs placebo	22 Fixed	Glabella, 20 U, 4 sites; temporal 20 U, 4 sites; frontal 10 U, 4 sites; suboccipital 30 U, 6 sites; trapezius 20 U, 4 sites	1	42.3	All CM	0/41
Hollanda 2014 (28)	38	PROSIGNE® 96 U (max) vs Placebo	12-24 FTP	Frontal 12-24 U, 4-8 sites; temporal 16-32 U, 4-8 sites; occipital 20-40 U, 4-8 sites	1	45.3(13.4)	All CM	0/38
Hou 2015 (29)	102	HENGLI® 25 U fixed vs HENGLI® 25 U acupoint vs placebo	10 Fixed	Fixed & placebo: frontal & occipital belly of occipitofrontalis, corrugator supercilii, temporalis & superior part of trapeziue. Acupoint: Yintang (EX-HN3), Taiyang (EX-HN5), Baihui (GV20), Shuaigu (GB8), Fengchi (GB20), Tianzhu (BL10); no. sites/units per region nr	1	40.7(9.0)	66EM/36CM	nr

Jabbari 2014 (38)	25	Botox 200-300 U vs placebo	22-24 Fixed	Frontalis, 40 U, 10 sites; temporalis, 60 U, 4 sites; occipitalis 10 U, 2 sites; cervical region: splenius cervicis, semispinalis capitis, trapezius muscles, 90-120 U, 6-10 sites	1	60	All CM	nr
Jost 2011 (30)	22	Botox® 10 U vs placebo (cross-over design)	2 FTP	Corrugator muscle and the occipitalis muscle of the side affected; no. sites/units per region nr	1	45.2(11.1)	nr	nr
Lauretti 2009 (31)	40	25 U Botox® vs 25 U Prosigne® vs 33.3 U Prosigne® vs saline	10 Fixed	Frontal region, 8 sites; temporal region, 2 sites; units per region nr	1	45.7(14.3)	All CM	nr
Mazza 2016 (39)	94	Botulinum toxin type A (Brand not reported) up to 200 U vs Placebo	nr FTP	Subcutaneous injections into trigeminal or occipital areas depending upon area of maximal pain; no. sites per region nr	2	nr	All CM	nr
Petri 2009 (32)	127	DYSPORT® 210 U vs DYSPORT® 80 U vs placebo	18 Fixed	Trapezius 45 U, splenius capitis 20 U, temporalis 20 U, frontalis 10 U, corrugator 10 U; no. sites per region nr	1	46.2(11.8)	All EM	0/127
Relja 2007 (33)	495	Botox® 225 U vs Botox ® 150 U vs Botox® 75 U vs Placebo	20 Fixed	Frontalis, 4 sites; corrugator, 2 sites; temporalis, 4 sites; splenius capitis, 2 sites; trapezius, 4 sites; semispinalis capitis, 2 sites; suboccipital region, 2 sites; units per region nr	3	43.2	All EM	0/495
Saper 2007 (34)	232	Botox® (25 U) all muscle sites vs Botox® (10 U) frontal vs Botox® (6 U) temporal vs Botox® (9 U) glabellar vs placebo all muscle sites	11 Fixed	Frontal, 4 sites; temporal, 2 sites; glabellar, 5 sites; units per region nr	1	43.6	All EM	0/232
Silberstein 2000 (35)	123	Botox® 75 U vs Botox® 25 U vs placebo	11 Fixed	Frontalis, 30 U; temporalis, 18 U; glabellar, 27 U; no. sites per region nr	1	44	All EM	0/123
Vo 2007 (37)	49	Botulinum toxin type A (Brand not reported) vs Placebo	22 Fixed	Corrugator 5 U, 2 sites; frontalis, 20 U, 4 sites; temporalis, 20 U (≥65 kg, 40 U), 4 sites; posterior neck, 60 U (≥65 kg, 90 U), 6 sites; occipitalis, 10 U, 2 sites; sternocleidomastoid, 20 U (≥65 kg, 40 U), 4 sites	1	42.4(8.3)	EM/CM ratio nr	nr

CM, chronic migraine; EM, episodic migraine; FTP, follow the pain; MOH, medication overuse headache; nr, not reported; OFTP, optional follow the pain.

Table 2: Characteristics of botulinum toxin vs. oral prophylactic agent studies

Trial (Reference)	Total N randomised	Comparison	No of injection sites (Fixed or FTP)	Location of injection sites	No. treatment cycles	Mean Age (SD)	EM/CM	Ratio of MOH/No MOH
Blumenfeld 2008 (41)	59	BOTOX ® ≤100 U vs DVPX;DEPAKOTE® 250 mg twice daily	nr FTP	Procerus, 2.5-5 U; Corrugators, 2.5-5 U; frontalis, 25 U; temporalis, 7.5-20 U; splenius capitis, 2.5-10 U; sternocleidomastoid, 7.5-15 U; trapezius, 2.5-5 U; occipitalis, 2.5-5 U; cervical paraspinalis, 7.5-15 U; semispinalis capitis, 5-10 U; masseter, 5-15 U	2	42.1(10.3)	45/14	0/59
Cady 2011 (42)	59	OnabotulinumtoxinA up to 200 U vs Topiramate 25 mg escalated to max 200 mg (ave 136 mg)	nr Fixed + nr OFTP	100 U fixed sites + up to 100 U in FTP	1	39.6	All CM	0/59
Mathew 2009 (43)	60	BOTOX® 200 U vs TOPAMAX® (topiramate) 100-200 mg/day	nr Fixed + nr OFTP	100 U fixed sites + up to 100 U in FTP	2	36.8(10.3)	All CM	nr

CM, chronic migraine; EM, episodic migraine; FTP, follow the pain; MOH, medication overuse headache; nr, not reported; OFTP, optional follow the pain.
