# Supplementary material for: Cochrane systematic review and meta-analysis of botulinum toxin for the prevention of migraine
Source: BMJ Open. 2019 Jul 16;9(7):e027953. doi: 10.1136/bmjopen-2018-027953 (PMC6661560; doi:10.1136/bmjopen-2018-027953)
Supplement: Supplementary file 4 [file bmjopen-2018-027953supp004.pdf]

BTX-A compared to placebo for the prevention of migraine in adults

Outcomes	Result with BTX-A (95% CI)	Number of participants (studies)	Quality of the evidence (GRADE)
Number of migraine days per month - Chronic migraine	MD 3.1 days lower (4.7 lower to 1.4 lower)	1497 (4 RCTs)	⊕⊕⊖⊖ LOW [a b]
Number of headache days per month - Chronic migraine	MD 1.9 days lower (2.7 lower to 1.0 lower)	1384 (2 RCTs)	⊕⊕⊕⊕ HIGH
Number of migraine attacks	MD 0.5 attacks lower (1.3 lower to 0.4 higher)	2004 (6 RCTs)	⊕⊕⊖⊖ LOW [c d]
Headache intensity measure - Chronic migraine (Visual Analogue Score 0-10)	MD 2.7 cm lower (3.3 lower to 2.1 lower)	75 (2 RCTs)	⊕⊖⊖⊖ VERY LOW [e f]
Headache intensity measure - Episodic migraine (Visual Analogue Score 0-10)	MD 4.9 cm lower (6.6 lower to 3.2 lower)	75 (1 RCT)	⊕⊖⊖⊖ VERY LOW [e f]
Headache Impact Test-6	MD 1.6 points higher (2.1 lower to 5.3 higher)	45 (1 RCT)	⊕⊖⊖⊖ VERY LOW [e f]
Total number of participants experiencing an adverse event	RR 1.28 (1.1 to 1.5)	3325 (13 RCTs)	⊕⊕⊕⊖ MODERATE [g]

Footnotes

CI: Confidence interval; RR: Risk ratio; MD: Mean difference. [a] Downgraded once due to inconsistency: Statistical heterogeneity observed despite similarities in populations and doses. [b] Downgraded once due to imprecision: Sensitivity analysis testing robustness of result suggested small studies may be over estimating treatment effect. [c] Downgraded once due to indirectness: Sensitivity of this outcome measure at risk of being too low to detect clinically

meaningful differences. [d] Downgraded once due to publication bias: Evidence found of trials that have never been published which record this outcome. [e] Downgraded once due to risk of bias: High or unclear risk of selective reporting bias and poor reporting of this outcome measure had a large effect on numbers analyzed. [f] Downgraded twice due to imprecision: Study size small, new trial evidence likely to change result. [g] Downgraded once due to imprecision: Study size small, new trial evidence likely to change result. GRADE Working Group grades of evidence- High quality: We are very confident that the true effect lies close to that of the estimate of the effect; Moderate quality: We are moderately confident in the effect estimate: The true effect is likely to be close to the estimate of the effect, but there is a possibility that it is substantially different; Low quality: Our confidence in the effect estimate is limited: The true effect may be substantially different from the estimate of the effect; Very low quality: We have very little confidence in the effect estimate: The true effect is likely to be substantially different from the estimate of effect
