# Supplementary material for: Cochrane systematic review and meta-analysis of botulinum toxin for the prevention of migraine
Source: BMJ Open. 2019 Jul 16;9(7):e027953. doi: 10.1136/bmjopen-2018-027953 (PMC6661560; doi:10.1136/bmjopen-2018-027953)
Supplement: Supplementary file 5 [file bmjopen-2018-027953supp005.pdf]

BTX-A compared to other established prophylactic agent for the prevention of migraine in adults

Outcomes	Result with BTX-A (95% CI)	Number of participants (studies)	Quality of the evidence (GRADE)
Number of migraine days per month - chronic migraine	One trial using topiramate in its comparison arm reported narratively on this outcome stating that there was no significant difference between groups.	43 (1 RCT)	⊕⊖⊖⊖ VERY LOW [a b c]
Number of headache days per month	MD 1 days lower (4.3 lower to 2.3 higher)	59 (1 RCT)	⊕⊖⊖⊖ VERY LOW [a b]
Headache intensity measure assessed with: 5-point scale, 5 being severe, 1 being mild - chronic migraine only	MD 0.4 points lower (0.79 lower to 0.01 lower)	46 (1 RCT)	⊕⊖⊖⊖ VERY LOW [a b]
Global impression of disease assessed with: Migraine impact and disability assessment scores	MD 4.3 points higher (28 lower to 37 higher)	101 (2 RCTs)	⊕⊖⊖⊖ VERY LOW [a b]
Total number of participants experiencing an adverse event	RR 0.8 (0.4 to 1.9)	114 (2 RCTs)	⊕⊖⊖⊖ VERY LOW [a b]

Footnotes

CI: Confidence interval; RR: Risk ratio; MD: Mean difference. [a] Downgraded once due to risk of bias: Unclear or high risk for selection, performance, detection and attrition bias. [b] Downgraded twice due to imprecision: Study sizes small, new trial evidence likely to change result. [c] Downgraded once due to imprecision: Narrative description only. GRADE

Working Group grades of evidence- High quality: We are very confident that the true effect lies close to that of the estimate of the effect; Moderate quality: We are moderately confident in the effect estimate: The true effect is likely to be close to the estimate of the effect, but there is a possibility that it is substantially different; Low quality: Our confidence in the effect estimate is limited: The true effect may be substantially different from the estimate of the effect; Very low quality: We have very little confidence in the effect estimate: The true effect is likely to be substantially different from the estimate of effect.
