# Supplementary material for: Cochrane systematic review and meta-analysis of botulinum toxin for the prevention of migraine
Source: BMJ Open. 2019 Jul 16;9(7):e027953. doi: 10.1136/bmjopen-2018-027953 (PMC6661560; doi:10.1136/bmjopen-2018-027953)
Supplement: Author's manuscript [file bmjopen-2018-027953.draft_revisions.pdf]

BMJ Open is committed to open peer review. As part of this commitment we make the peer review history of every article we publish publicly available.

When an article is published we post the peer reviewers' comments and the authors' responses online. We also post the versions of the paper that were used during peer review. These are the versions that the peer review comments apply to.

The versions of the paper that follow are the versions that were submitted during the peer review process. They are not the versions of record or the final published versions. They should not be cited or distributed as the published version of this manuscript.

BMJ Open is an open access journal and the full, final, typeset and author-corrected version of record of the manuscript is available on our site with no access controls, subscription charges or pay-per-view fees (http://bmjopen.bmj.com).

If you have any questions on BMJ Open's open peer review process please email info.bmjopen@bmj.com

BMJ Open

Cochrane systematic review and meta-analysis of Botulinum toxin for the prevention of migraine.

Journal:	*BMJ Open*
Manuscript ID	bmjopen-2018-027953
Article Type:	Research
Date Submitted by the Author:	22-Nov-2018
Complete List of Authors:	Herd, Clare; University of Birmingham, Institute of Applied Health Research<br>Tomlinson, Claire; University of Birmingham, BCTU<br>Rick, Caroline; University of Birmingham, BCTU<br>Scotton, William; University of Birmingham, Institute of Metabolism and Systems Research<br>Edwards, Julie; Sandwell and West Birmingham Hospitals NHS Trust, Department of Neurology,<br>Ives, Natalie; University of Birmingham, BCTU<br>Clarke, Carl; University of Birmingham, Neurology<br>Sinclair, AJ; University of Birmingham, Institute of Metabolism and Systems Research
Keywords:	Migraine Disorders, Botulinum toxin, Botox, Systematic Review, Randomised Controlled Trial

SCHOLARONE™
Manuscripts

Cochrane systematic review and meta-analysis of Botulinum toxin for the prevention of migraine.

Clare P Herd[1], Claire L Tomlinson[2], Caroline Rick[2], William J Scotton[3], Julie Edwards[4], Natalie Ives[2], Carl E Clarke[1,4], Alexandra Sinclair[3]

[1]Institute of Applied Health Research, University of Birmingham, Birmingham, UK.

[2]Birmingham Clinical Trials Unit, University of Birmingham, Birmingham, UK.

[3]Institute of Metabolism and Systems Research, University of Birmingham, Birmingham, UK

[4]Department of Neurology, Sandwell and West Birmingham Hospitals NHS Trust, Birmingham, UK

C.P.Herd@bham.ac.uk; C.L.Smith.1@bham.ac.uk; C.E.Rick@bham.ac.uk; W.Scotton@bham.ac.uk; julie.edwards14@nhs.net; N.J.Ives@bham.ac.uk; C.E.Clarke@bham.ac.uk;

Corresponding Author: A.B.Sinclair@bham.ac.uk

Word count: 4,304 (inclusive of abstract)

Keywords: Migraine Disorders, Botulinum toxin, Botox, Systematic Review, Randomised Controlled Trial

Data sharing statement

Full data analysis and trial summaries available in Cochrane review DOI: 10.1002/14651858.CD011616.pub2

For peer review only

Abstract

Objectives

To assess the effects of botulinum toxin for prevention of migraine in adults.

Participants

A total of 4190 adults with chronic or episodic migraine, with or without the additional diagnosis of medication overuse headache, were included in the trials reviewed.

Interventions

Botulinum toxin compared with placebo, active treatment or clinically relevant different dose.

Primary and secondary outcome measures

The primary outcome measure was number of migraine days per month. Diary data covering frequency, intensity, and duration of migraines and use of rescue medication, as well as global impression scales, quality of life rating scales, cost effectiveness and adverse events were included as secondary outcome measures.

Design

Cochrane methods were used to review randomized, double-blind, controlled trials. Twelve week post-treatment time-point data was analyzed.

Results

Twenty-eight trials (N=4192) were included. Trial quality was mixed. Botulinum toxin treatment resulted in reduced frequency of -3.1 migraine days/month (95% confidence interval -4.73 to -1.41, N=1497) in chronic migraineurs compared with placebo. An improvement was seen in migraine severity, measured on a numerical rating scale 0-10 with 10 being maximal pain, of -2.70 cm (95% confidence interval -3.31 to -2.09, N=75) and -4.9 cm (95% confidence interval -6.56 to -3.24, N=32) for chronic and episodic migraine respectively. Botulinum toxin had a relative risk of treatment related adverse events twice

For peer review only

that of placebo, but a reduced risk compared to active comparators (relative risk 0.76, 95% confidence interval 0.59 to 0.98) and a low withdrawal rate (3%). Although individual trials reported non-inferiority to oral treatments, insufficient data were available for meta-analysis of effectiveness outcomes.

Conclusions

In chronic migraine, botulinum toxin reduces migraine frequency by three days/month and has a favorable safety profile. Inclusion of medication overuse headache does not preclude its effectiveness. Evidence to support or refute efficacy in episodic migraine was not identified.

Strengths and limitations

- This paper is a summary of a Cochrane review conducted using systematic and thorough methodology to identify and synthesize all available evidence for the effectiveness of botulinum toxin for prophylactic treatment of migraine.
- No language or date restrictions were placed on the search strategy.
- Many of the included studies were small in size and failed to fully report their data which impacted the quality ratings and the content of the meta-analyses.
- Our chosen primary outcome measure, though recommended in current guidelines for controlled trials of prophylactic treatment of chronic migraine, was not commonly recorded.

For peer review only

Introduction

Migraine is the seventh leading cause of years lived with disability globally and is estimated to affect around 15% of the worlds population.[1] Days lost from work and other activities of daily living resulting from migraines have a major economic impact.[2] Many people with migraine suffer prolonged and frequent migraine attacks despite optimised acute and prophylactic treatments.[3-5]

Botulinum toxin type A (BTX-A) has been licensed for use in migraine in some countries, based largely on two commercially sponsored trials.[6 7] The recommended reconstituted dose is 155–195 units, administered intramuscularly as 0.1 ml (5 units) injections to between 31 and 39 sites around the head and neck.[3] Cost of treatment and administration of BTX-A is much higher than standard doses of the two first line treatments for the prevention of migraine, propranolol and topiramate (around 25 times and 15 times respectively in the UK).[8-10]

Migraine can be categorized as chronic or episodic and these terms are commonly used in eligibility criteria for clinical trials and systematic reviews. Chronic migraine is currently defined by the International Headache Society (IHS) as headache for at least 15 days per month with migraine features on eight of those days.[11] Episodic migraine is commonly used to describe patients with symptoms of migraine who have less than 15 headache days per month and according to official guidance is a term which can be used for migraine that is not covered by the definition of chronic migraine.[11] Migraine can occur with medication overuse headache; the IHS definition has evolved, but currently this is defined as an interaction between a therapeutic agent used excessively and a susceptible patient.[11 12] Trials recruiting participants with chronic migraine will come across many patients with this dual diagnosis. Current UK NICE guidelines recommend the use of BTX-A for chronic migraine, but not for

For peer review only

high frequency episodic migraine, and only when the condition is 'appropriately managed' for medication overuse.[8]

The aim of this evidence review was to assess the effects of botulinum toxin (BTX) versus placebo or alternative active treatment for the prophylaxis of episodic migraine or chronic migraine in adults.

This paper is a summary of key aspects from a Cochrane review first published in The Cochrane Library 2018, Issue 6 (see http://www.thecochranelibrary.com/ for information).[13] Cochrane reviews are regularly updated as new evidence emerges and in response to feedback, and The Cochrane Library should be consulted for the most recent version of the review.

Methods

The protocol for this review was published in the Cochrane Database of Systematic Reviews in advance of the publication of the full review which replaced it. Deviations from the protocol are listed in the full review.[13]

Search strategy

A systematic search of the literature published before December 2017 was carried out. We designed a highly sensitive search strategy using methods recommended by the Cochrane collaboration to minimize publication bias. No date, language or publication status restrictions were applied. We used a combination of index terms and free text terms for headache, migraine, cephalalgia or hemicrania; and botulinum toxin, botox, onabotulinum toxin, oculinum or clostridium botulinum. Relevant trials were identified through electronic searches of Cochrane Central Register of Controlled Trials, Medline (see full strategy in supplemental file 1), Embase, clinicaltrials.gov and World Health Organization International clinical trials registry, hand-searching reference lists and citation searches on key

For peer review only

publications, and correspondence with all major manufacturers of BTX products relevant to this review.

Selection criteria

We included randomized, double-blind, controlled trials of people over the age of 18 years suffering from migraine as defined by any edition of the IHS criteria,[11 12 14] or meeting reasonable criteria designed to distinguish between migraine and tension-type headache. Patients with both chronic migraine and episodic migraine were included in this review. Medication overuse headache was included as these types of participants have been included in large and prominent trials in this area. Trials must compare BTX (any sero-type) injected into the head and neck muscles with placebo injections, clinically relevant different dose of same treatment or active preventative agent. Trials allowing the use of concomitant preventative or rescue treatments were included.

Screening of abstracts and assessment of eligibility of full papers were carried out independently in duplicate and according to criteria predefined in the peer reviewed protocol. If disagreements occurred at any stage, a third author considered the available information or if necessary the study authors were contacted for clarification. When eligibility could not be determined through consideration of published materials or contact with trial authors the studies were excluded.

Quality assessment

Eligible material was assessed, independently by two reviewers for each trial, for methodological quality using Cochrane risk of bias methods. Publications were assessed on their method of randomization, blinding and concealment of allocation, the number of participants lost to follow-up, evidence of selective reporting and study size.

Data extraction

For peer review only

Data extraction was carried out independently and in duplicate onto forms designed and tested at protocol stage. The primary outcome was frequency of migraine days per month. Secondary outcomes included: frequency of headache days, frequency of migraine attacks, severity of migraine, duration of migraine, 50% responder rate, global impression scales, quality of life measures and adverse event reporting. We used risk ratios (RRs) as the preferred statistical output for dichotomous outcomes, with 95% confidence intervals (CIs). For continuous data, we used mean differences (MDs) with 95% CIs. Results with p values lower than 0.05 were considered to be statistically significant. Twelve week time-point data following final round of treatment was analyzed. We sought data from the first phase for any cross-over trials identified. We attempted to contact authors and obtain missing data.

Statistical analysis

The review authors assessed trial baseline characteristics to identify clinical heterogeneity during the extraction of trial information. If clinical and methodological homogeneity were confirmed, we carried out meta-analysis of the data. We tested for statistical homogeneity of pooled estimates of effectiveness using the Chi^2 test and the I^2 statistic, for which a statistically significant (P value $\leq$0.1) value of the Chi^2 test together with I^2 value of at least 50% indicates heterogeneity.

Heterogeneity present in doses, injection sites and participant populations led to the decision that a random-effects model should be used for the analysis. Within our eligible comparisons, we split data into migraine classification subgroups in order to show results for chronic migraine, episodic migraine and a mixed group for which the diagnosis could not be split. We planned to use the following subgroups to test for variation in the effects of the intervention:

1. Trials including medication overuse headache versus trials excluding this type of patient.

For peer review only

2. Different sero-types of BTX (e.g. A versus B) and within sero-types (Dysport® versus Botox®).

3. Different types of agents for the prevention of migraine versus BTX.

4. Accepted and licensed 31 injection pattern versus other injection patterns used.

At least two trials and 200 participants per group were required for any particular subgroup analysis to be carried out.

We carried out sensitivity analyses for our primary outcome only. Prevailing evidence suggests that smaller trials are more likely to report stronger effect estimates than large trials.[15 16] To assess whether these stronger effect estimates reflected the true treatment effect we carried out a sensitivity analysis in which we examined the effect of removing studies at high risk of bias from study size.

We assessed the validity of our findings as well as the level of confidence suitable to any estimates of effect generated by our analyses using the Grading of Recommendations Assessment, Development and Evaluation (GRADE) approach.[17]

Patient and Public Involvement

There was no patient or public involvement in the design or reviewing process. However, the final Cochrane manuscript including a lay summary, which is accessible to the public through the Cochrane library, was reviewed by a patient representative as part of the editorial process. Their feedback was incorporated into the final draft.

Results

Description of included studies

The flow of information through the review process is given in the PRISMA flow chart in supplemental file 2.

For peer review only

We identified 28 eligible trials, involving a total of 4192 participants, which were eligible for inclusion in this review. Twenty-three of these trials compared BTX type-A with placebo injections [6 7 18-38] and three compared with an alternative established oral prophylactic agent.[39-41]

Five trials, reported in four articles, compared alternative doses of BTX type-A,[24 31-33] all but one of these also included a placebo arm[24] and one compared with injections of histamine.[42] Due to the paucity of the data, review of the dosing studies and the histamine study are included as appendices in the Cochrane review and is not repeated here.[13]

The results of the critical appraisal were mixed (fig 1). Across all domains poor reporting was an issue and in all but attrition bias and study size at least 50% of trials provided insufficient information to allow judgments about risk of bias to be made. Only two trials were at low risk of bias due to study size (at least 200 participants per trial arm) and these two trials were also at low risk of bias across all other domains.[6 7]

INSERT FIGURE 1

Figure 1: Risk of bias graph: review authors' judgements about each risk of bias item presented as percentages across all included studies.

Sixteen trials were commercially sponsored, including the only two trials at low risk from study size.[6 7 19 20 22-25 30-33 38 39 41]

For those trials providing information on the migraine diagnosis of their participants the ratio of chronic/episodic migraine was 1872/1928, leaving 392 included participants unclassified and analyzed as 'Mixed'. The mean age was 42 years and 85% of all participants were female. Pregnant women were generally explicitly excluded. All included trials used BTX type-A, of these 21 had at least one arm treated with the Botox® formulation,[6 7 18-22 24 25 28 29 31-33 36 38-42] two used Dysport®,[23 30] two used Prosigne®,[26 29] and one HengLi®.[27] The range of doses administered in trials of Botox® was 6 U to 300 U. The trials using Dysport®

For peer review only

administered doses of 80 U up to 240 U in treated arms (dose equivalency reported by trial publications: 2 to 3 U:1 U Botox®). HengLi® and Prosigne® trials used doses ranging from 25 U to 96 U (dose equivalency reported by trial publications: 1 U:1 U Botox®).

Effectiveness versus placebo

Comparison with a placebo group was made in 23 trials with 3912 participants.

Meta-analysis of our primary outcome for the four trials in chronic migraine which reported it showed that there was a reduction of 3.1 days of migraine per month (95% CI -4.7 to -1.4) in favor of BTX type-A treatment (fig 2). At least 60% of the participants in this analysis had medication overuse headache. The episodic migraine subgroup involved only one trial of 418 participants which showed no difference in the number of migraine days between treated and placebo groups (P=0.49). Insufficient data were available to carry out any of the planned subgroup analyses on the primary outcome measure. Concern about small trial effects caused us to carry out a sensitivity analysis. Removal of all chronic migraine trials at high risk of bias from study size left just the two PREEMT trials, which gave a more conservative reduction of 2.0 days per month (95% CI -2.8 to -1.1).

INSERT FIGURE 2

Figure 2: Comparison of BTX type-A versus placebo in relation to number of migraine days per month.

Migraine severity score on a 10 cm visual analogue scale (VAS), improved by -3.30 cm (95% CI -4.16 to -2.45) more with active treatment (fig 3). Only four small trials reported meta-analyzable data for this outcome. For Chronic migraine the improvement was -2.70 cm (95% CI -3.31 to -2.09, N=75), and for episodic migraine it was -4.9 cm (95% CI -6.56 to -3.24, N=34).

INSERT FIGURE 3

Figure 3: Comparison of BTX type-A versus placebo in relation to severity of migraine measured on a 10 cm visual analogue scale.

For peer review only

A reduction in the number headache days per month of 1.9 days (95% CI -2.7 to -1.0, 2 trials, N = 1384) in favor of BTX type-A treatment was also seen. However data for number of migraine attacks from six trials of both chronic migraine and episodic migraine participants (N = 2004) showed no significant between group difference (P = 0.30). Duration of migraine in hours was fully reported by only one trial showing a greater reduction of -5.1 hours (95% CI -6.2 to -4.0) for 102 chronic and episodic migraine participants. A further four trials with 420 participants reported no significant difference between groups for this outcome. Global assessment measures and quality of life measures were poorly reported and it was not possible to carry out statistical analysis of these outcome measures.

Effectiveness of BTX versus oral prophylactic agents

Three trials with 178 participants compared Botox® injections with oral prophylactic agents using double dummy techniques. Two trials compared 100 U fixed dose plus optional dose of up to 100 U of Botox® with topiramate maximum dose 200 mg/day.[40 41] The third trial compared treatment with up to 100 U Botox® with sodium valproate 250 mg twice daily.[39] Fourteen of the 178 participants had episodic migraine, all other participants had chronic migraine. Where meta-analysis was possible we pooled data from these three trials as there were insufficient data to allow us to explore comparisons with individual drug types or effects on chronic migraine and episodic migraine populations.

The primary outcome, number of migraine days per month was recorded in only one of the active comparison trials. The trialists reported that there was no statistically significant difference between treatment with BTX type-A and topiramate for this outcome.[41]

The number of headache days per month was recorded in two trials. No difference in number of headache days per month between treatment with BTX type-A and sodium valproate was reported (P=0.55).[35] No data were reported but it was stated that there was also no statistically significant difference between BTX type-A and topiramate treated groups.[40] A 5-

For peer review only

point scale was used to compare the effect of BTX type-A with alternative agents in two trials, Blumenfeld et al reported no significant difference and Mathew et al reported within group analysis only.[39 41] Number of migraine attacks and duration of migraine were not reported by any trial. No difference between BTX type-A and topiramate was stated for use of rescue medications.[41]

Of all the secondary outcome measures, data for meta-analysis were available only for the Migraine disability assessment (MIDAS) scores. Results of this showed no significant difference in change scores between the established drug treatments and injection with Botox® (P = 0.80, 2 trials, N = 101).

Safety

BTX type-A had an RR of treatment related adverse events of twice that seen for placebo (2.18, 95% CI 1.73 to 2.75, 6 trials, N=2839) (fig 4). All of these events were transient and non-serious, the most common being blepharoptosis, muscle weakness, injection site pain and neck pain.

INSERT FIGURE 4

Figure 4: Comparison of BTX type-A versus placebo in relation to treatment related adverse events.

Compared with oral treatments, BTX type-A showed a reduced RR of treatment related adverse events of 0.76 (95% CI 0.59 to 0.98, 2 trials, N=73). There was also difference in favor of BTX type-A in the RR of withdrawing due to adverse events of 0.28 (95% CI 0.10 to 0.79; I2 = 0%) which is a RR reduction of 72%.

A low withdrawal rate of 3% for BTX type-A was generated using data from all those trials treating with more than one injection cycle irrespective of the type of comparison arm.

Quality of the Evidence

The quality of the evidence assessed using GRADE methods was varied but mostly low and very low; the primary outcome measure was low and very low quality evidence for the

For peer review only

placebo and active control comparisons respectively. Small trial size, high risk of bias and unexplained heterogeneity were common reasons for downgrading the quality of the evidence. All judgements and reasons for gradings are given in Supplemental files 3 and 4.

Discussion

Evidence was identified to support the use of injections of BTX type-A into the head and neck muscles, to reduce the number of migraine days experienced per month. Mean frequency of migraine days was significantly reduced by 3 days per month more by BTX type-A treatment than by placebo. All patients included in this analysis had chronic migraine and so had a high baseline frequency with an average of 20 days per month quoted by the two largest trials in the analysis.[6 7] For patients with chronic migraine, likely to be refractory to first and second line treatment, a 3 day improvement may well represent a meaningful difference. BTX type-A groups also fared better than placebo in the frequency of headache days by 2 days per month. Severity of migraine measured on a visual analogue scale was improved by 3 cm for chronic migraine and 5 cm for episodic migraine on a 10 cm scale. Though these results were from few small trials and the estimate is considered to be low quality evidence, the differences in severity scores were in excess of the minimal clinically important difference of 1.2 cm determined by Kelly *et al.*[43] and indicate that the treatment is reducing the impact of each migraine attack. In contrast to this no significant difference from placebo was observed for frequency of migraine attacks. Patient and clinician reported global assessment scales and quality of life scales were underused and when they were incorporated into trials they were poorly reported, so no aggregation of data of this type comparing investigative treatment with placebo was possible in this review.

It was not possible to carry out any analysis on headache diary outcomes or severity measures for head-to-head comparisons between BTX type-A and other established agents due to lack

of available data. MIDAS scores for 101 patients from two small trials, one comparing Botox® with topiramate and one with sodium valproate were available and these showed no significant between group difference (P=0.8).

Trials included in this review commonly state that BTX's have good safety profiles and the evidence from the 23 trials included in this review which reported adverse events in some form support those assertions. Although an increased risk of experiencing treatment related adverse events was found for the BTX type-A treated group compared with placebo, the event types were non-serious and transient.

A relative risk reduction (RRR) of 24% in treatment related adverse events in favor of BTX type-A was found when comparing with topiramate and sodium valproate in two trials. These two trials found an RRR in favor of BTX type-A of 72% for withdrawal rate due to adverse events. Percentage withdrawals due to adverse events for all of those trials included in this review which used more than one round of BTX type-A injections, irrespective of the comparison arm type, was 3%. The data sets for the direct comparisons with other prophylactic agents were small, but the relationship is supported by the indirect comparison of this percentage with published rates of 20% for topiramate and 12% for sodium valproate.[44 45] This result suggests that patients tolerate this treatment better than the oral alternatives.

Reporting was generally poor, with only six of 28 trials reporting data on our primary outcome in a usable format, and an additional five providing data for frequency of migraine attacks. These two outcomes are recommended as primary outcomes by the trial guidelines produced by the IHS and should be fully reported to allow individual trials to be placed in the context of the totality of the evidence.[46] A large proportion of the recorded data were missing from the published reports of our included trials. Failure to fully report data in trial publications led to problems throughout the meta-analysis and greater confidence in the

For peer review only

conclusions would have been possible if all trials that recorded our outcomes of interest had fully reported them.

Prophylactic treatments for migraine aim to reduce the frequency, duration and/or the intensity of attacks. Frequency of migraine attacks was commonly used as the primary outcome particularly in studies carried out before the publication of the PREEMT trials. Use of this measure may mask an important improvement in symptoms seen in the form of shorter and less intense migraine attacks. Use of the more sensitive measures, number of days or hours spent with migraine per month coupled with a measure of intensity, may enable detection of such changes and could be particularly relevant to episodic migraine patients for whom attacks may be shorter at baseline. Another problem with focusing on this outcome measure was the failure generally to define what was meant by a migraine attack, and therefore, the likelihood of variation in the definitions used across the trials.

Neither efficacy nor safety data were available for long term treatment with BTX. The longest treatment period in any of the studies included in this review was three treatments with 12 weeks between treatments, so we cannot know the implications of treating patients with BTX over a period longer than 9 months.

Most trials did not report whether or not they had included patients with medication overuse symptoms and those that did stated they had largely excluded medication overuse patients. Pooled data for the two PREEMPT trials for the chronic migraine plus medication overuse subgroup (N=906) showed that the difference between groups for both migraine and headache day frequencies was 2 days (P<.001) in favor of treatment with BTX.[47] The medication overuse subgroup result falls within the confidence intervals of the pooled estimate generated by this review for the same outcome measure in combined populations with and without medication overuse headache. It would appear from these data that the

For peer review only

inclusion of patients with medication overuse does not change the effectiveness of BTX for prophylactic treatment of migraine.

Conclusions

We have data which suggest that BTX effectively reduces the duration and severity of migraines in sufferers. There are however question marks over the quality of the evidence. Efficacy measures were commonly reported as showing non-inferiority of BTX to topiramate and sodium valproate and the withdrawal rate from BTX is much lower than that for first line prophylactic treatments for migraine. So should we be using more BTX?

It is currently recommended by NICE guidance that medication overuse headache should be addressed before treatment with BTX but trial data suggests it is efficacious in chronic migraine patients with untreated medication overuse headache. So although treatment of medication overuse headache is good practice, perhaps it should not be a requirement before prescription of BTX. NICE recommends the use of BTX to treat chronic migraine that has not responded to at least three prior pharmacological prophylaxis therapies. The confidence in the effectiveness of these drugs is arguably no greater than that for BTX and patients seem better able to tolerate BTX.[4 5 44 45] If, as is suggested by trial data, BTX has the equivalent efficacy to other agents but lower withdrawal rates, then if it were not for the higher cost, BTX would likely be recommended as an earlier preventative treatment for chronic migraine. The difference between chronic and episodic migraine diagnoses is arbitrary and so there is no pathophysiological reason that treatment with BTX would be efficacious in people with 15 days headache per month and inefficacious in people with 14 days of headache per month in a stepwise fashion. The treatment may well be useful for episodic migraine, particularly in high frequency episodic migraine, but data is lacking.

Acknowledgements

For peer review only

We would like to thank Joanne Abbott, Information Specialist at the Cochrane Pain, Palliative and Supportive Care Group, who ran searches in MEDLINE, Embase and CENTRAL. We would also like to thank Ana Hughes, Cancer Clinical Trials Unit, University of Birmingham for her translation of Blumenkron 2006.

Author contributions

CEC and AS conceived the review. CH ran searches not covered by the review group's information specialist. CH, CLT, CR and AS screened the search results. CH, CLT, CR, WS, CEC and AS assessed the quality of studies and extracted data. CH contacted trial authors, managed data and entered it into RevMan, and carried out data analysis. NI provided statistical advice. CH, CLT, CR, NI, CEC and AS were involved in interpretation of the results. All authors read and edited final version of the review.

Funding

The authors received no financial support for the research, authorship, or publication of this article.

AJS is funded by an NIHR Clinician Scientist Fellowship (NIHR-CS-011-028), by the Medical Research Council, UK (MR/K015184/1)

Competing interests

JE received funding from Allergan in 2017 to attend a Master Class in Botulinum toxin. For all remaining authors none declared.

References

1. Collaborators GDaIIaP. Global, regional, and national incidence, prevalence, and years lived with disability for 310 diseases and injuries, 1990-2015: a systematic analysis

For peer review only

for the Global Burden of Disease Study 2015. Lancet (London, England) 2016;**388**(10053):1545-602.
2. Linde M, Gustavsson A, Stovner LJ, et al. The cost of headache disorders in Europe: the Eurolight project. European Journal of Neurology 2012;**19**:703-11.
3. Linde K, Rossnagel K. Propranolol for migraine prophylaxis. Cochrane Database of Systematic Reviews 2004(2).
4. Linde M, Mulleners WM, Chronicle EP, et al. Gabapentin or pregabalin for the prophylaxis of episodic migraine in adults. Cochrane Database of Systematic Reviews 2013(6).
5. Linde M, Mulleners WM, Chronicle EP, et al. Antiepileptics other than gabapentin, pregabalin, topiramate, and valproate for the prophylaxis of episodic migraine in adults. Cochrane Database of Systematic Reviews 2013(6).
6. Aurora SK, Dodick DW, Turkel CC, et al. OnabotulinumtoxinA for treatment of chronic migraine: results from the double-blind, randomized, placebo-controlled phase of the PREEMPT 1 trial. Cephalalgia : an international journal of headache 2010;**30**(7):793-803.
7. Diener HC, Dodick DW, Aurora SK, et al. OnabotulinumtoxinA for treatment of chronic migraine: Results from the double-blind, randomized, placebo-controlled phase of the PREEMPT 2 trial. Cephalalgia 2010;**30**(7):804-14.
8. National Institute for Health Care Excellence. Botulinum toxin type A for the prevention of headaches in adults with chronic migraine. NICE technology appraisal guidance [TA260]. Published date: June 2012. Available from http://wwwniceorguk/guidance/ta260.
9. National Institute for Health Care Excellence. Headaches: Diagnosis and management of headaches in young people and adults. NICE guidelines [CG150]. Published date: September 2012. Available from http://wwwniceorguk/guidance/cg150.
10. British Medical Association, Society RP. British National Formulary (BNF). Available from wwwbnforg.
11. Headache Classification Committee of the International Headache S. The international classification of headache disorders, 3rd edition. Cephalalgia 2013;**33**(9):629-808.
12. Headache Classification Committee of the International Headache Society. The International Classification of Headache Disorders, 2nd edition, 1st revision. Cephalalgia 2004;**24**(Suppl 1):1-160.
13. Herd CP, Tomlinson CL, Rick C, et al. Botulinum toxins for the prevention of migraine in adults. Cochrane Database Syst Rev 2018;**6**:CD011616.
14. Headache Classification Committee of the International Headache Society. Classification and diagnostic criteria for headache disorders, cranial neuralgias and facial pain. Cephalalgia 1988;**8**(Suppl. 7):1-96.
15. Dechartres A, Trinquart L, Boutron I, et al. Influence of trial sample size on treatment effect estimates: meta-epidemiological study. BMJ 2013;**346**:f2304-f04.
16. Nüesch E, Trelle S, Reichenbach S, et al. Small study effects in meta-analyses of osteoarthritis trials: Meta-epidemiological study. BMJ 2010;**341**:c3515-c15.
17. Guyatt GH, Oxman AD, Vist GE, et al. GRADE: an emerging consensus on rating quality of evidence and strength of recommendations. BMJ 2008;**336**(7650):924-26.
18. Anand KS, Prasad A, Singh MM, et al. Botulinum toxin type A in prophylactic treatment of migraine. American journal of therapeutics 2006;**13**(3):183-7.
19. Aurora SK, Gawel M, Brandes JL, et al. Botulinum toxin type a prophylactic treatment of episodic migraine: a randomized, double-blind, placebo-controlled exploratory study. Headache 2007;**47**(4):486-99.

For peer review only

20. Barrientos N, Chana P. Botulinum toxin type A in prophylactic treatment of migraine headaches: A preliminary study. Journal of headache and pain 2003;**4**(3):146-51.
21. Blumenkron D, Rivera C, Cuevas C. Efficacy of botulinum toxin type A in patients with migraine. [Spanish] Eficacia del tratamiento con toxina botulinica tipo A en pacientes con migrana. Medicina Interna de Mexico 2006;**22**(1):25-31.
22. Cady R, Schreiber C. Botulinum toxin type A as migraine preventive treatment in patients previously failing oral prophylactic treatment due to compliance issues. Headache 2008;**48**(6):900-13.
23. Chankrachang S, Arayawichanont A, Poungvarin N, et al. Prophylactic botulinum type A toxin complex (Dysport®) for migraine without aura. Headache 2011;**51**(1):52-63.
24. Elkind AH, O'Carroll P, Blumenfeld A, et al. A series of three sequential, randomized, controlled studies of repeated treatments with botulinum toxin type A for migraine prophylaxis. The journal of pain : official journal of the American Pain Society 2006;**7**(10):688-96.
25. Freitag FG, Diamond S, Diamond M, et al. Botulinum Toxin Type A in the treatment of chronic migraine without medication overuse. Headache 2008;**48**(2):201-9.
26. Hollanda L, Monteiro L, Melo AS. Botulinum toxin type A for cephalic cutaneous allodynia in chronic migraine: A randomized, double-blinded, placebo-controlled trial. Neurology international 2014;**6**(4):70-3.
27. Hou M, Xie JF, Kong XP, et al. Acupoint injection of onabotulinumtoxin a for migraines. Toxins 2015;**7**(11):4442-54.
28. Jost WH. Low-dosed botulinum toxin a in the prophylactic management of unilateral migraine: A randomized double-blind placebo-controlled crossover study. Open pain journal 2011;**4**(1):4-7.
29. Lauretti GR, Rosa CP, Kitayama A, et al. Comparison of Botox® or Prosigne® and Facial Nerve Blockade as Adjuvant in Chronic Migraine. Journal of Biomedical Science and Engineering 2014;**7**:446-52.
30. Petri S, Tölle T, Straube A, et al. Botulinum toxin as preventive treatment for migraine: a randomized double-blind study. European neurology 2009;**62**(4):204-11.
31. Relja M, Poole AC, Schoenen J, et al. A multicentre, double-blind, randomized, placebo-controlled, parallel group study of multiple treatments of botulinum toxin type A (BoNTA) for the prophylaxis of episodic migraine headaches. Cephalalgia : an international journal of headache 2007;**27**(6):492-503.
32. Saper JR, Mathew NT, Loder EW, et al. A double-blind, randomized, placebo-controlled comparison of botulinum toxin type a injection sites and doses in the prevention of episodic migraine. Pain medicine (Malden, Mass) 2007;**8**(6):478-85.
33. Silberstein S, Mathew N, Saper J, et al. Botulinum toxin type A as a migraine preventive treatment. Headache 2000;**40**(6):445-50.
34. Cady R, Turner I, Dexter K, et al. An exploratory study of salivary calcitonin gene-related peptide levels relative to acute interventions and preventative treatment with onabotulinumtoxinA in chronic migraine. Headache 2014;**54**(2):269-77.
35. Vo AH, Satori R, Jabbari B, et al. Botulinum toxin type-a in the prevention of migraine: a double-blind controlled trial. Aviation, space, and environmental medicine 2007;**78**(5 Suppl):B113-8.
36. Jabbari B. Investigation of Efficacy and Safety of Botulinum Toxin A (Botox-Allergan Inc) in Migraine Headaches. clinicaltrials.gov, 2008.
37. Mazza MR, Ferrigno G, Vescio B, et al. Subcutaneous botulinum toxin type a treatment for prophylaxis of headaches in chronic migraine: A new therapeutic strategy. Cephalalgia 2016;**36**:35.

For peer review only

38. Allergan. Use of a Treatment Benefit Questionnaire in Patients With Chronic Migraine Treated With OnabotulinumtoxinA (BOTOX®). clinicaltrials.gov, 2013.
39. Blumenfeld AM, Schim JD, Chippendale TJ. Botulinum toxin type A and divalproex sodium for prophylactic treatment of episodic or chronic migraine. Headache 2008;**48**(2):210-20.
40. Cady RK, Schreiber CP, Porter JA, et al. A multi-center double-blind pilot comparison of onabotulinumtoxinA and topiramate for the prophylactic treatment of chronic migraine. Headache 2011;**51**(1):21-32.
41. Mathew NT, Jaffri SF. A double-blind comparison of onabotulinumtoxina (BOTOX) and topiramate (TOPAMAX) for the prophylactic treatment of chronic migraine: a pilot study. Headache 2009;**49**(10):1466-78.
42. Millán-Guerrero RO, Isais-Millán S, Barreto-Vizcaíno S, et al. Subcutaneous histamine versus botulinum toxin type A in migraine prophylaxis: a randomized, double-blind study. European journal of neurology 2009;**16**(1):88-94.
43. Kelly AM. The minimum clinically significant difference in visual analogue scale pain score does not differ with severity of pain. Emerg Med J 2001;**18**:205–07.
44. Linde M, Mulleners WM, Chronicle EP, et al. Valproate (valproic acid or sodium valproate or a combination of the two) for the prophylaxis of episodic migraine in adults. Cochrane Database of Systematic Reviews 2013(6).
45. Linde M, Mulleners WM, Chronicle EP, et al. Topiramate for the prophylaxis of episodic migraine in adults. Cochrane Database of Systematic Reviews 2013(6).
46. Tfelt-Hansen P, Pascual J, Ramadan N, et al. Guidelines for controlled trials of drugs in migraine: third edition. A guide for investigators. Cephalalgia 2012;**32**(1):6-38.
47. Silberstein SD, Blumenfeld AM, Cady RK, et al. OnabotulinumtoxinA for treatment of chronic migraine: PREEMPT 24-week pooled subgroup analysis of patients who had acute headache medication overuse at baseline. Journal of the neurological sciences 2013;**331**(1-2):48-56.

For peer review only

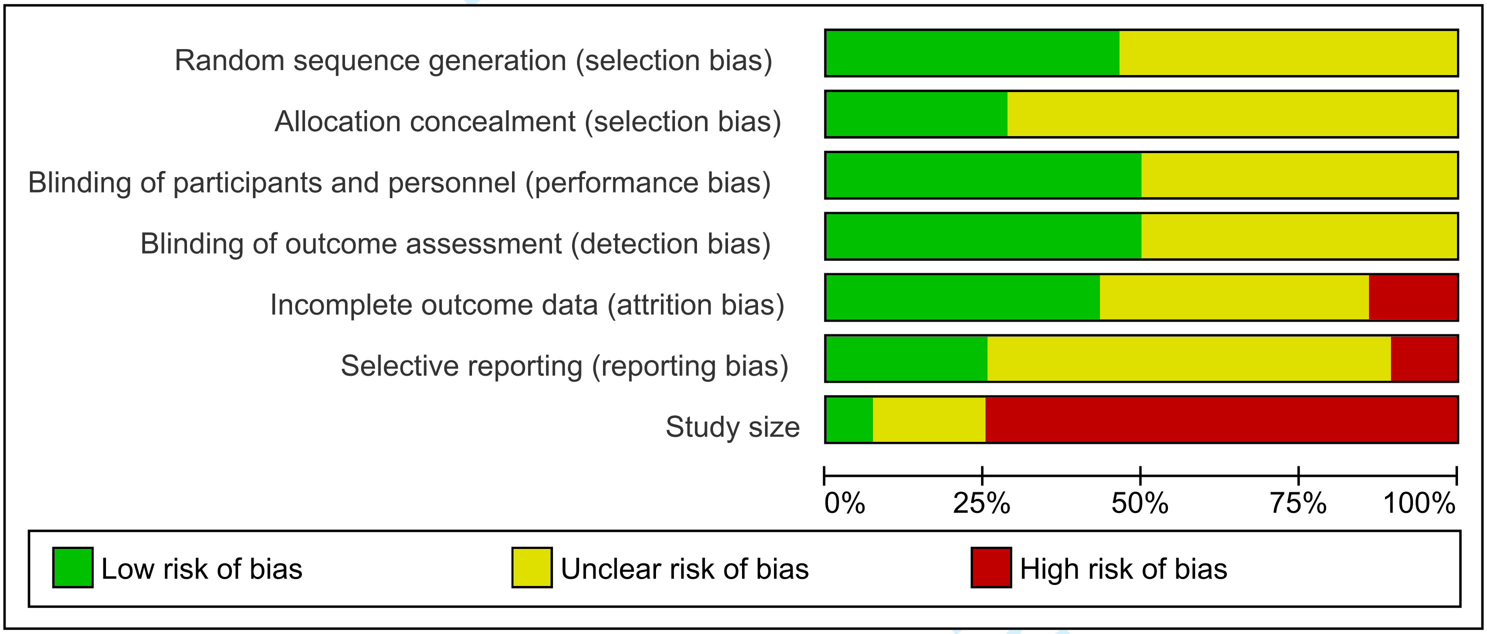

For peer review only - http://bmjopen.bmj.com/site/about/guidelines.xhtml

	Botulinum toxin			Placebo				Mean Difference
**Study or Subgroup**	**Mean [Days]**	**SD [Days]**	**Total**	**Mean [Days]**	**SD [Days]**	**Total**	**Weight**	**IV, Random, 95% CI [Days]**
**1.1.1 Chronic migraine**								
Aurora 2010 (PREEMPT 1)	-7.6	6.5	341	-6.1	6.8	338	24.3%	-1.50 [-2.50, -0.50]
Cady 2014	-10.1	5.9	9	-5.2	5.1	10	7.6%	-4.90 [-9.88, 0.08]
Diener 2010 (PREEMPT 2)	-8.7	6.6	347	-6.3	6.7	358	24.4%	-2.40 [-3.38, -1.42]
Mazza 2016	9.4	8.6	63	15.1	1.8	31	17.9%	-5.70 [-7.92, -3.48]
**Subtotal (95% CI)**			**760**			**737**	**74.1%**	**-3.07 [-4.73, -1.41]**
Heterogeneity: Tau² = 1.84; Chi² = 12.56, df = 3 (P = 0.006); I² = 76%								
Test for overall effect: Z = 3.62 (P = 0.0003)								
**1.1.2 Episodic migraine**								
Elkind I 2006	-1.4	2.5	312	-1.2	2.6	106	25.9%	-0.20 [-0.77, 0.37]
**Subtotal (95% CI)**			**312**			**106**	**25.9%**	**-0.20 [-0.77, 0.37]**
Heterogeneity: Not applicable								
Test for overall effect: Z = 0.69 (P = 0.49)								
**Total (95% CI)**			**1072**			**843**	**100.0%**	**-2.39 [-4.02, -0.76]**
Heterogeneity: Tau² = 2.59; Chi² = 35.80, df = 4 (P < 0.00001); I² = 89%								
Test for overall effect: Z = 2.88 (P = 0.004)								
Test for subgroup differences: Chi² = 10.26, df = 1 (P = 0.001), I² = 90.3%								

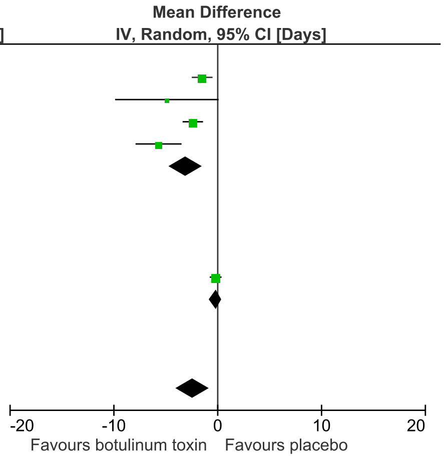

For peer review only

	Botulinum toxin			Placebo				Mean Difference
**Study or Subgroup**	**Mean**	**SD**	**Total**	**Mean**	**SD**	**Total**	**Weight**	**IV, Random, 95% CI**
**1.4.1 Chronic migraine**								
Hollanda 2014	3.45	2.39	20	6.17	2.5	18	18.3%	-2.72 [-4.28, -1.16]
Lauretti 2014	6.5	1.3	28	9.2	0.7	9	36.6%	-2.70 [-3.36, -2.04]
**Subtotal (95% CI)**			**48**			**27**	**54.9%**	**-2.70 [-3.31, -2.09]**
Heterogeneity: Tau² = 0.00; Chi² = 0.00, df = 1 (P = 0.98); I² = 0%								
Test for overall effect: Z = 8.67 (P < 0.00001)								
**1.4.2 Episodic migraine**								
Anand 2006	2.4	3.2	16	7.3	1.1	16	17.0%	-4.90 [-6.56, -3.24]
**Subtotal (95% CI)**			**16**			**16**	**17.0%**	**-4.90 [-6.56, -3.24]**
Heterogeneity: Not applicable								
Test for overall effect: Z = 5.79 (P < 0.00001)								
**1.4.3 Mixed**								
Hou 2015	2.8	1.2	83	6.3	2.2	19	28.1%	-3.50 [-4.52, -2.48]
**Subtotal (95% CI)**			**83**			**19**	**28.1%**	**-3.50 [-4.52, -2.48]**
Heterogeneity: Not applicable								
Test for overall effect: Z = 6.71 (P < 0.00001)								
**Total (95% CI)**			**147**			**62**	**100.0%**	**-3.30 [-4.16, -2.45]**
Heterogeneity: Tau² = 0.40; Chi² = 6.74, df = 3 (P = 0.08); I² = 55%								
Test for overall effect: Z = 7.58 (P < 0.00001)								
Test for subgroup differences: Chi² = 6.74, df = 2 (P = 0.03), I² = 70.3%								

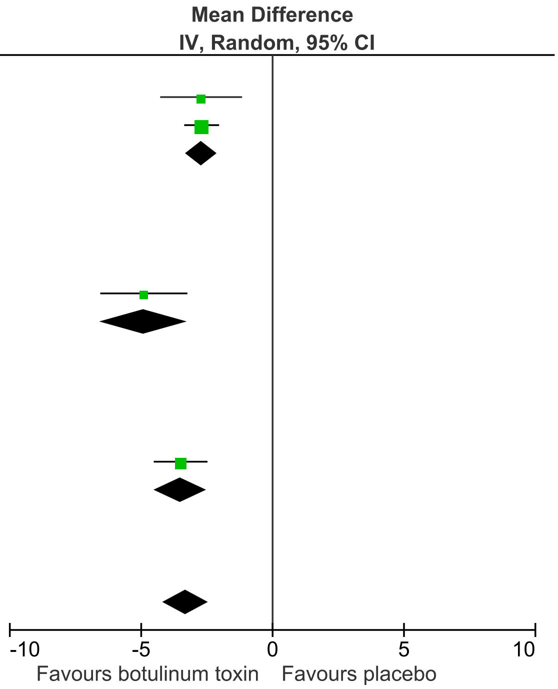

Study or Subgroup	Botulinum toxin Events	Botulinum toxin Total	Placebo Events	Placebo Total	Weight	Risk Ratio M-H, Random, 95% CI
**1.11.1 Chronic migraine**						
Aurora 2010 (PREEMPT 1)	86	340	39	334	18.6%	2.17 [1.53, 3.06]
Diener 2010 (PREEMPT 2)	116	347	49	358	20.8%	2.44 [1.81, 3.30]
**Subtotal (95% CI)**		**687**		**692**	**39.3%**	**2.32 [1.85, 2.91]**
Total events	202		88			
Heterogeneity: Tau² = 0.00; Chi² = 0.26, df = 1 (P = 0.61); I² = 0%						
Test for overall effect: Z = 7.28 (P < 0.00001)						
**1.11.2 Episodic migraine**						
Aurora 2007	113	187	39	182	20.7%	2.82 [2.09, 3.81]
Elkind I 2006	61	312	7	106	7.3%	2.96 [1.40, 6.27]
Relja 2007	243	377	37	118	21.9%	2.06 [1.56, 2.71]
Saper 2007	45	187	11	45	10.8%	0.98 [0.55, 1.75]
**Subtotal (95% CI)**		**1063**		**451**	**60.7%**	**2.06 [1.37, 3.08]**
Total events	462		94			
Heterogeneity: Tau² = 0.11; Chi² = 11.01, df = 3 (P = 0.01); I² = 73%						
Test for overall effect: Z = 3.50 (P = 0.0005)						
**Total (95% CI)**		**1750**		**1143**	**100.0%**	**2.18 [1.73, 2.75]**
Total events	664		182			
Heterogeneity: Tau² = 0.04; Chi² = 11.41, df = 5 (P = 0.04); I² = 56%						
Test for overall effect: Z = 6.59 (P < 0.00001)						
Test for subgroup differences: Chi² = 0.26, df = 1 (P = 0.61), I² = 0%						

Risk Ratio
M-H, Random, 95% CI

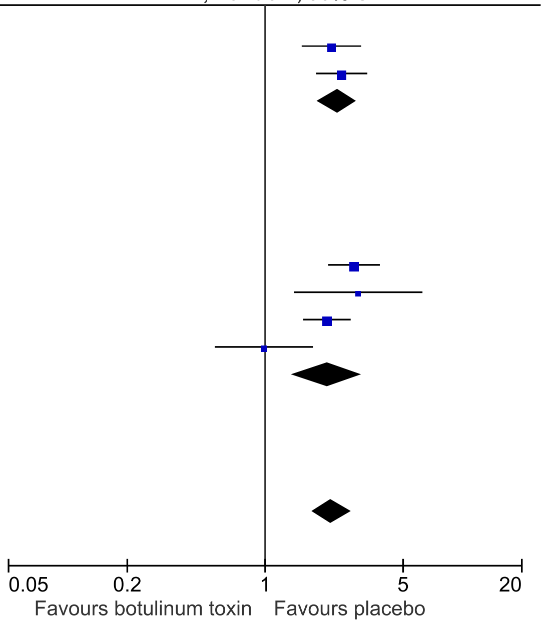

MEDLINE (via OVID)

#1 Exp headache disorders/

#2 headache/

#3 (headache* or migrain* or cephalgi* or cephalalgi* or hemicrani*).mp.

#4 or/1-3

#5 exp botulinum toxins/

#6 (botulin* adj toxin*).tw

#7 (botulinum* or oculinu* or boto* or onabotulinum*).tw.

#8 exp botulinum toxin type A/

#9 Exp clostridium botulinum/

#10 clostridium botulin*.tw.

#11 or/5-10

12 randomized controlled trial.pt.

13 controlled clinical trial.pt.

14 randomized.ab.

15 placebo.ab.

16 drug therapy.fs.

17 randomly.ab.

18 trial.ab.

19 groups.ab.

20 12 or 13 or 14 or 15 or 16 or 17 or 18 or 19

21 exp animals/ not humans.sh.

22 20 not 21

23 4 and 11 and 22

For peer review only

PRISMA 2009 Flow Diagram

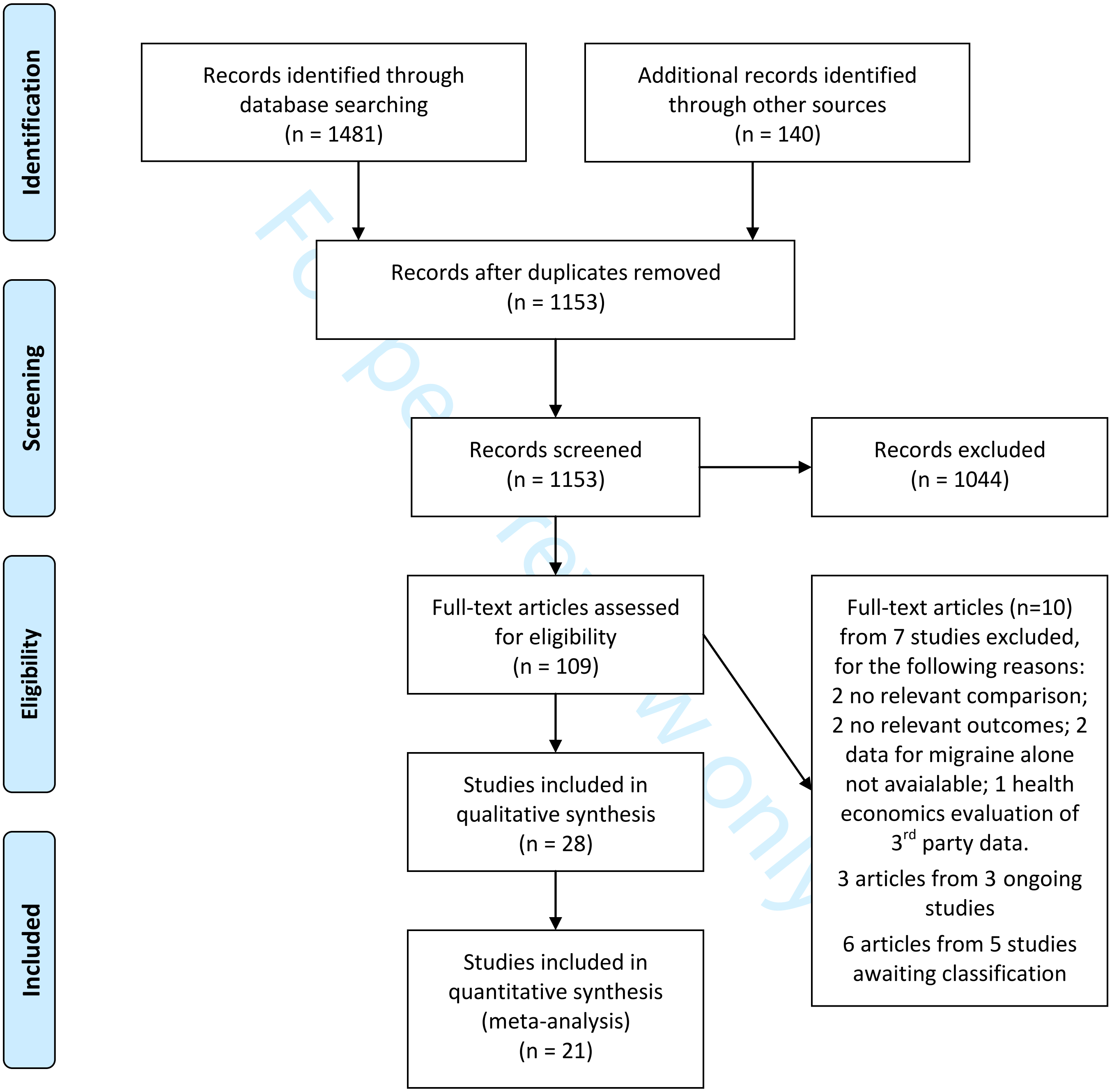

From: Moher D, Liberati A, Tetzlaff J, Altman DG, The PRISMA Group (2009). *P*referred *R*eporting *I*tems for *S*ystematic Reviews and *M*eta-*A*nalyses: The PRISMA Statement. PLoS Med 6(7): e1000097. doi:10.1371/journal.pmed1000097

For more information, visit www.prisma-statement.org.

For peer review only

BTX-A compared to placebo for the prevention of migraine in adults

Outcomes	Result with BTX-A (95% CI)	Number of participants (studies)	Quality of the evidence (GRADE)
Number of migraine days per month - Chronic migraine	MD 3.1 days lower (4.7 lower to 1.4 lower)	1497 (4 RCTs)	⊕⊕⊝⊝ LOW [a b]
Number of headache days per month - Chronic migraine	MD 1.9 days lower (2.7 lower to 1.0 lower)	1384 (2 RCTs)	⊕⊕⊕⊕ HIGH
Number of migraine attacks	MD 0.5 attacks lower (1.3 lower to 0.4 higher)	2004 (6 RCTs)	⊕⊕⊝⊝ LOW [c d]
Headache intensity measure - Chronic migraine (Visual Analogue Score 0-10)	MD 2.7 cm lower (3.3 lower to 2.1 lower)	75 (2 RCTs)	⊕⊝⊝⊝ VERY LOW [e f]
Headache intensity measure - Episodic migraine (Visual Analogue Score 0-10)	MD 4.9 cm lower (6.6 lower to 3.2 lower)	75 (1 RCT)	⊕⊝⊝⊝ VERY LOW [e f]
Headache Impact Test-6	MD 1.6 points higher (2.1 lower to 5.3 higher)	45 (1 RCT)	⊕⊝⊝⊝ VERY LOW [e f]
Total number of participants experiencing an adverse event	RR 1.28 (1.1 to 1.5)	3325 (13 RCTs)	⊕⊕⊕⊝ MODERATE [g]

Footnotes

CI: Confidence interval; RR: Risk ratio; MD: Mean difference. [a] Downgraded once due to inconsistency: Statistical heterogeneity observed despite similarities in populations and doses. [b] Downgraded once due to imprecision: Sensitivity analysis testing robustness of result suggested small studies may be over estimating treatment effect. [c] Downgraded once due to indirectness: Sensitivity of this outcome measure at risk of being too low to detect clinically

For peer review only

meaningful differences. [d] Downgraded once due to publication bias: Evidence found of trials that have never been published which record this outcome. [e] Downgraded once due to risk of bias: High or unclear risk of selective reporting bias and poor reporting of this outcome measure had a large effect on numbers analyzed. [f] Downgraded twice due to imprecision: Study size small, new trial evidence likely to change result. [g] Downgraded once due to imprecision: Study size small, new trial evidence likely to change result. GRADE Working Group grades of evidence- High quality: We are very confident that the true effect lies close to that of the estimate of the effect; Moderate quality: We are moderately confident in the effect estimate: The true effect is likely to be close to the estimate of the effect, but there is a possibility that it is substantially different; Low quality: Our confidence in the effect estimate is limited: The true effect may be substantially different from the estimate of the effect; Very low quality: We have very little confidence in the effect estimate: The true effect is likely to be substantially different from the estimate of effect

For peer review only

BTX-A compared to other established prophylactic agent for the prevention of migraine in adults

Outcomes	Result with BTX-A (95% CI)	Number of participants (studies)	Quality of the evidence (GRADE)
Number of migraine days per month - chronic migraine	One trial using topiramate in its comparison arm reported narratively on this outcome stating that there was no significant difference between groups.	43 (1 RCT)	⨁⊖⊖⊖ VERY LOW [a b c]
Number of headache days per month	MD 1 days lower (4.3 lower to 2.3 higher)	59 (1 RCT)	⨁⊖⊖⊖ VERY LOW [a b]
Headache intensity measure assessed with: 5-point scale, 5 being severe, 1 being mild - chronic migraine only	MD 0.4 points lower (0.79 lower to 0.01 lower)	46 (1 RCT)	⨁⊖⊖⊖ VERY LOW [a b]
Global impression of disease assessed with: Migraine impact and disability assessment scores	MD 4.3 points higher (28 lower to 37 higher)	101 (2 RCTs)	⨁⊖⊖⊖ VERY LOW [a b]
Total number of participants experiencing an adverse event	RR 0.8 (0.4 to 1.9)	114 (2 RCTs)	⨁⊖⊖⊖ VERY LOW [a b]

Footnotes

CI: Confidence interval; RR: Risk ratio; MD: Mean difference. [a] Downgraded once due to risk of bias: Unclear or high risk for selection, performance, detection and attrition bias. [b] Downgraded twice due to imprecision: Study sizes small, new trial evidence likely to change result. [c] Downgraded once due to imprecision: Narrative description only. GRADE

For peer review only

Working Group grades of evidence- High quality: We are very confident that the true effect lies close to that of the estimate of the effect; Moderate quality: We are moderately confident in the effect estimate: The true effect is likely to be close to the estimate of the effect, but there is a possibility that it is substantially different; Low quality: Our confidence in the effect estimate is limited: The true effect may be substantially different from the estimate of the effect; Very low quality: We have very little confidence in the effect estimate: The true effect is likely to be substantially different from the estimate of effect.

For peer review only

PRISMA 2009 Checklist

Section/topic	#	Checklist item	Reported on page #
**TITLE**			
Title	1	Identify the report as a systematic review, meta-analysis, or both.	1
**ABSTRACT**			
Structured summary	2	Provide a structured summary including, as applicable: background; objectives; data sources; study eligibility criteria, participants, and interventions; study appraisal and synthesis methods; results; limitations; conclusions and implications of key findings; systematic review registration number.	1-2
**INTRODUCTION**			
Rationale	3	Describe the rationale for the review in the context of what is already known.	2-3
Objectives	4	Provide an explicit statement of questions being addressed with reference to participants, interventions, comparisons, outcomes, and study design (PICOS).	3
**METHODS**			
Protocol and registration	5	Indicate if a review protocol exists, if and where it can be accessed (e.g., Web address), and, if available, provide registration information including registration number.	3
Eligibility criteria	6	Specify study characteristics (e.g., PICOS, length of follow-up) and report characteristics (e.g., years considered, language, publication status) used as criteria for eligibility, giving rationale.	4
Information sources	7	Describe all information sources (e.g., databases with dates of coverage, contact with study authors to identify additional studies) in the search and date last searched.	3-4
Search	8	Present full electronic search strategy for at least one database, including any limits used, such that it could be repeated.	Suppl.file 1
Study selection	9	State the process for selecting studies (i.e., screening, eligibility, included in systematic review, and, if applicable, included in the meta-analysis).	4
Data collection process	10	Describe method of data extraction from reports (e.g., piloted forms, independently, in duplicate) and any processes for obtaining and confirming data from investigators.	5
Data items	11	List and define all variables for which data were sought (e.g., PICOS, funding sources) and any assumptions and simplifications made.	5
Risk of bias in individual studies	12	Describe methods used for assessing risk of bias of individual studies (including specification of whether this was done at the study or outcome level), and how this information is to be used in any data synthesis.	5
Summary measures	13	State the principal summary measures (e.g., risk ratio, difference in means).	5
Synthesis of results	14	Describe the methods of handling data and combining results of studies, if done, including measures of consistency (e.g., I^2) for each meta-analysis.	5

For peer review only

PRISMA 2009 Checklist

Section/topic	#	Checklist item	Reported on page #
Risk of bias across studies	15	Specify any assessment of risk of bias that may affect the cumulative evidence (e.g., publication bias, selective reporting within studies).	6
Additional analyses	16	Describe methods of additional analyses (e.g., sensitivity or subgroup analyses, meta-regression), if done, indicating which were pre-specified.	6
**RESULTS**			
Study selection	17	Give numbers of studies screened, assessed for eligibility, and included in the review, with reasons for exclusions at each stage, ideally with a flow diagram.	6 + suppl.file2
Study characteristics	18	For each study, present characteristics for which data were extracted (e.g., study size, PICOS, follow-up period) and provide the citations.	7
Risk of bias within studies	19	Present data on risk of bias of each study and, if available, any outcome level assessment (see item 12).	7
Results of individual studies	20	For all outcomes considered (benefits or harms), present, for each study: (a) simple summary data for each intervention group (b) effect estimates and confidence intervals, ideally with a forest plot.	8-10
Synthesis of results	21	Present results of each meta-analysis done, including confidence intervals and measures of consistency.	8-10
Risk of bias across studies	22	Present results of any assessment of risk of bias across studies (see Item 15).	11+suppl. Files 3&4
Additional analysis	23	Give results of additional analyses, if done (e.g., sensitivity or subgroup analyses, meta-regression [see Item 16]).	8
**DISCUSSION**			
Summary of evidence	24	Summarize the main findings including the strength of evidence for each main outcome; consider their relevance to key groups (e.g., healthcare providers, users, and policy makers).	11
Limitations	25	Discuss limitations at study and outcome level (e.g., risk of bias), and at review-level (e.g., incomplete retrieval of identified research, reporting bias).	11-13
Conclusions	26	Provide a general interpretation of the results in the context of other evidence, and implications for future research.	14
**FUNDING**			
Funding	27	Describe sources of funding for the systematic review and other support (e.g., supply of data); role of funders for the systematic review.	15

From: Moher D, Liberati A, Tetzlaff J, Altman DG, The PRISMA Group (2009). Preferred Reporting Items for Systematic Reviews and Meta-Analyses: The PRISMA Statement. PLoS Med 6(7): e1000097. doi:10.1371/journal.pmed1000097

For more information, visit: **www.prisma-statement.org**.

For peer review only - http://bmjopen.bmj.com/site/about/guidelines.xhtml

For peer review only

BMJ Open

Cochrane systematic review and meta-analysis of Botulinum toxin for the prevention of migraine.

Journal:	*BMJ Open*
Manuscript ID	bmjopen-2018-027953.R1
Article Type:	Research
Date Submitted by the Author:	04-Apr-2019
Complete List of Authors:	Herd, Clare; University of Birmingham, Institute of Applied Health Research<br>Tomlinson, Claire; University of Birmingham, BCTU<br>Rick, Caroline; University of Nottingham, Nottingham Clinical Trials Unit<br>Scotton, William; University of Birmingham, Institute of Metabolism and Systems Research<br>Edwards, Julie; Sandwell and West Birmingham Hospitals NHS Trust, Department of Neurology,<br>Ives, Natalie; University of Birmingham, BCTU<br>Clarke, Carl; University of Birmingham, Neurology<br>Sinclair, AJ; University of Birmingham, Institute of Metabolism and Systems Research
**Primary Subject Heading**:	Neurology
Secondary Subject Heading:	Medical management
Keywords:	Migraine Disorders, Botulinum toxin, Botox, Systematic Review, Randomised Controlled Trial

SCHOLARONE™
Manuscripts

Cochrane systematic review and meta-analysis of Botulinum toxin for the prevention of migraine.

Clare P Herd[1], Claire L Tomlinson[2], Caroline Rick[3], William J Scotton[4], Julie Edwards[5], Natalie Ives[2], Carl E Clarke[1,5], Alexandra Sinclair[4]

[1]Institute of Applied Health Research, University of Birmingham, Birmingham, UK.

[2]Birmingham Clinical Trials Unit, University of Birmingham, Birmingham, UK.

[3]Nottingham Clinical Trials Unit, University of Nottingham, Nottingham, UK

[4]Institute of Metabolism and Systems Research, University of Birmingham, Birmingham, UK

[5]Department of Neurology, Sandwell and West Birmingham Hospitals NHS Trust, Birmingham, UK

C.P.Herd@bham.ac.uk; C.L.Smith.1@bham.ac.uk; C.E.Rick@bham.ac.uk; W.Scotton@bham.ac.uk; julie.edwards14@nhs.net; N.J.Ives@bham.ac.uk; C.E.Clarke@bham.ac.uk;

Corresponding Author: A.B.Sinclair@bham.ac.uk

Word count: 4,360 (inclusive of abstract)

Keywords: Migraine Disorders, Botulinum toxin, Botox, Systematic Review, Randomised Controlled Trial

Data sharing statement

Full data analysis and trial summaries available in Cochrane review DOI: 10.1002/14651858.CD011616.pub2

For peer review only

Abstract

Objectives

To assess the effects of botulinum toxin for prevention of migraine in adults.

Participants

A total of 4190 adults with chronic or episodic migraine, with or without the additional diagnosis of medication overuse headache, were included in the trials reviewed.

Interventions

Botulinum toxin compared with placebo, active treatment or clinically relevant different dose.

Primary and secondary outcome measures

The primary outcome measure was number of migraine days per month. Diary data covering frequency, intensity, and duration of migraines and use of rescue medication, as well as global impression scales, quality of life rating scales, cost effectiveness and adverse events were included as secondary outcome measures.

Design

Cochrane methods were used to review randomized, double-blind, controlled trials. Twelve week post-treatment time-point data was analyzed.

Results

Twenty-eight trials (N=4192) were included. Trial quality was mixed. Botulinum toxin treatment resulted in reduced frequency of -2.0 migraine days/month (95% confidence interval -2.8 to -1.1, N=1384) in chronic migraineurs compared with placebo. An improvement was seen in migraine severity, measured on a numerical rating scale 0-10 with 10 being maximal pain, of -2.70 cm (95% confidence interval -3.31 to -2.09, N=75) and -4.9 cm (95% confidence interval -6.56 to -3.24, N=32) for chronic and episodic migraine respectively. Botulinum toxin had a relative risk of treatment related adverse events twice

For peer review only

that of placebo, but a reduced risk compared to active comparators (relative risk 0.76, 95% confidence interval 0.59 to 0.98) and a low withdrawal rate (3%). Although individual trials reported non-inferiority to oral treatments, insufficient data were available for meta-analysis of effectiveness outcomes.

Conclusions

In chronic migraine, botulinum toxin reduces migraine frequency by three days/month and has a favorable safety profile. Inclusion of medication overuse headache does not preclude its effectiveness. Evidence to support or refute efficacy in episodic migraine was not identified.

Strengths and limitations

- This paper is a summary of a Cochrane review conducted using systematic and thorough methodology to identify and synthesize all available evidence for the effectiveness of botulinum toxin for prophylactic treatment of migraine.
- No language or date restrictions were placed on the search strategy.
- Many of the included studies were small in size and failed to fully report their data which impacted the quality ratings and the content of the meta-analyses.
- Our chosen primary outcome measure, though recommended in current guidelines for controlled trials of prophylactic treatment of chronic migraine, was not commonly recorded.

For peer review only

Introduction

Migraine is the seventh leading cause of years lived with disability globally and is estimated to affect around 15% of the world's population.[1] Days lost from work and other activities of daily living resulting from migraines have a major economic impact.[2] Many people with migraine suffer prolonged and frequent migraine attacks despite optimised acute and prophylactic treatments.[3-5]

Botulinum toxin type A (BTX-A) has been licensed for use in migraine in some countries, based largely on two commercially sponsored trials.[6 7] The recommended reconstituted dose is 155–195 units, administered intramuscularly as 0.1 ml (5 units) injections to between 31 and 39 sites around the head and neck.[3] Cost of treatment and administration of BTX-A is much higher than standard doses of the two first line treatments for the prevention of migraine, propranolol and topiramate (around 25 times and 15 times respectively in the UK).[8-10]

Migraine can be categorized as chronic or episodic and these terms are commonly used in eligibility criteria for clinical trials and systematic reviews. Chronic migraine is currently defined by the International Headache Society (IHS) as headache for at least 15 days per month with migraine features on eight of those days.[11] Episodic migraine is commonly used to describe patients with symptoms of migraine who have less than 15 headache days per month and according to official guidance is a term which can be used for migraine that is not covered by the definition of chronic migraine.[11] Migraine can occur with medication overuse headache; the IHS definition has evolved, but currently this is defined as an interaction between a therapeutic agent used excessively and a susceptible patient.[11 12] Trials recruiting participants with chronic migraine will come across many patients with this dual diagnosis. Current UK NICE guidelines recommend the use of BTX-A for chronic migraine, but not for

For peer review only

high frequency episodic migraine, and only when the condition is 'appropriately managed' for medication overuse.[8]

The aim of this evidence review was to assess the effects of botulinum toxin (BTX) versus placebo or alternative active treatment for the prophylaxis of episodic migraine or chronic migraine in adults.

This paper is a summary of key aspects from a Cochrane review first published in The Cochrane Library 2018, Issue 6 (see http://www.thecochranelibrary.com/ for information).[13] Cochrane reviews are regularly updated as new evidence emerges and in response to feedback, and The Cochrane Library should be consulted for the most recent version of the review.

Methods

The protocol for this review was published in the Cochrane Database of Systematic Reviews in advance of the publication of the full review which replaced it. Deviations from the protocol are listed in the full review.[13]

Search strategy

A systematic search of the literature published before March 2019 was carried out. We designed a highly sensitive search strategy using methods recommended by the Cochrane collaboration to minimize publication bias. No date, language or publication status restrictions were applied. We used a combination of index terms and free text terms for headache, migraine, cephalalgia or hemicrania; and botulinum toxin, botox, onabotulinum toxin, oculinum or clostridium botulinum. Relevant trials were identified through electronic searches of Cochrane Central Register of Controlled Trials, Medline (see full strategy in supplemental file 1), Embase, clinicaltrials.gov and World Health Organization International clinical trials registry, hand-searching reference lists and citation searches on key

For peer review only

publications, and correspondence with all major manufacturers of BTX products relevant to this review.

Selection criteria

We included randomized, double-blind, controlled trials of people over the age of 18 years suffering from migraine as defined by any edition of the IHS criteria,[11 12 14] or meeting reasonable criteria designed to distinguish between migraine and tension-type headache. Patients with both chronic migraine and episodic migraine were included in this review. Medication overuse headache was included as these types of participants have been included in large and prominent trials in this area. Trials must compare BTX (any sero-type) injected into the head and neck muscles with placebo injections, clinically relevant different dose of same treatment or active preventative agent. Trials allowing the use of concomitant preventative or rescue treatments were included.

Screening of abstracts and assessment of eligibility of full papers were carried out independently in duplicate and according to criteria predefined in the peer reviewed protocol. If disagreements occurred at any stage, a third author considered the available information or if necessary the study authors were contacted for clarification. When eligibility could not be determined through consideration of published materials or contact with trial authors the studies were excluded.

Quality assessment

Eligible material was assessed, independently by two reviewers for each trial, for methodological quality using Cochrane risk of bias methods. Publications were assessed on their method of randomization, blinding and concealment of allocation, the number of participants lost to follow-up, evidence of selective reporting and study size.

Data extraction

For peer review only

Data extraction was carried out independently and in duplicate onto forms designed and tested at protocol stage. The primary outcome was frequency of migraine days per month. Secondary outcomes included: frequency of headache days, frequency of migraine attacks, severity of migraine, duration of migraine, 50% responder rate, global impression scales, quality of life measures and adverse event reporting. We used risk ratios (RRs) as the preferred statistical output for dichotomous outcomes, with 95% confidence intervals (CIs). For continuous data, we used mean differences (MDs) with 95% CIs. Results with p values lower than 0.05 were considered to be statistically significant. Twelve week time-point data following final round of treatment was analyzed. We sought data from the first phase for any cross-over trials identified. We attempted to contact authors and obtain missing data.

Statistical analysis

The review authors assessed trial information and baseline characteristics to identify clinical and methodological differences during the data extraction process. If clinical and methodological homogeneity were confirmed, we carried out meta-analysis of the data using Review Manager (RevMan) 5.3.[15]

Heterogeneity present in doses, injection sites and participant populations led to the decision that a random-effects model should be used for the analysis. RevMan implements a version of random-effects meta-analysis that is described by Dersimonian and Laird[16] and presents an estimate of the between-study variance (Tau^2) at the bottom of each forest plot. We tested for statistical homogeneity of pooled estimates of effectiveness using the Chi^2 test and the I^2 statistic, for which a statistically significant (P value ≤ 0.1) value of the Chi^2 test together with I^2 value of at least 50% indicates heterogeneity.

Within our eligible comparisons, we split data into migraine classification subgroups in order to show results for chronic migraine, episodic migraine and a mixed group for which the diagnosis could not be split.

For peer review only

We planned to use the following subgroups to test for variation in the effects of the intervention:

1. Trials including medication overuse headache versus trials excluding this type of patient.
2. Different sero-types of BTX (e.g. A versus B) and within sero-types (Dysport® versus Botox®).
3. Different types of agents for the prevention of migraine versus BTX.
4. Accepted and licensed 31 injection pattern versus other injection patterns used.

At least two trials and 200 participants per group were required for any particular subgroup analysis to be carried out.

We carried out sensitivity analyses for our primary outcome only. Prevailing evidence suggests that smaller trials are more likely to report stronger effect estimates than large trials.[17 18] To assess whether these stronger effect estimates reflected the true treatment effect we carried out a sensitivity analysis in which we examined the effect of removing studies at high risk of bias from study size.

We assessed the validity of our findings as well as the level of confidence suitable to any estimates of effect generated by our analyses using the Grading of Recommendations Assessment, Development and Evaluation (GRADE) approach.[19]

Patient and Public Involvement

There was no patient or public involvement in the design or reviewing process. However, the final Cochrane manuscript including a lay summary, which is accessible to the public through the Cochrane library, was reviewed by a patient representative as part of the editorial process. Their feedback was incorporated into the final draft.

Results

For peer review only

Description of included studies

The flow of information through the review process is given in the PRISMA flow chart in supplemental file 2.

We identified 28 eligible trials, involving a total of 4192 participants, which were eligible for inclusion in this review. Twenty-three of these trials compared BTX type-A with placebo injections [6 7 20-40] and three compared with an alternative established oral prophylactic agent.[41-43]

Five trials, reported in four articles, compared alternative doses of BTX type-A,[26 33-35] all but one of these also included a placebo arm[26] and one compared with injections of histamine.[44] Due to the paucity of the data, review of the dosing studies and the histamine study are included as appendices in the Cochrane review and is not repeated here.[13]

The results of the critical appraisal were mixed (fig 1). Across all domains poor reporting was an issue and in all but attrition bias and study size at least 50% of trials provided insufficient information to allow judgments about risk of bias to be made. Only two trials were at low risk of bias due to study size (at least 200 participants per trial arm) and these two trials were also at low risk of bias across all other domains.[6 7]

INSERT FIGURE 1

Sixteen trials were commercially sponsored, including the only two trials at low risk from study size.[6 7 21 22 24-27 32-35 40 41 43]

For those trials providing information on the migraine diagnosis of their participants the ratio of chronic/episodic migraine was 1872/1928, leaving 392 included participants unclassified and analyzed as 'Mixed'. The mean age was 42 years and 85% of all participants were female. Pregnant women were generally explicitly excluded. All included trials used BTX type-A, of these 21 had at least one arm treated with the Botox® formulation,[6 7 20-24 26 27 30 31 33-35 38 40-44] two used Dysport®,[25 32] two used Prosigne®,[28 31] and one HengLi®.[29] The range of

For peer review only

doses administered in trials of Botox® was 6 U to 300 U. The trials using Dysport® administered doses of 80 U up to 240 U in treated arms (dose equivalency reported by trial publications: 2 to 3 U:1 U Botox®). HengLi® and Prosigne® trials used doses ranging from 25 U to 96 U (dose equivalency reported by trial publications: 1 U:1 U Botox®).

Effectiveness versus placebo

Comparison with a placebo group was made in 23 trials with 3912 participants.

Meta-analysis of our primary outcome for the four trials in chronic migraine which reported it showed that there was a reduction of 3.1 days of migraine per month (95% CI -4.7 to -1.4) in favor of BTX type-A treatment (fig 2). At least 60% of the participants in this analysis had medication overuse headache. The episodic migraine subgroup involved only one trial of 418 participants which showed no difference in the number of migraine days between treated and placebo groups (P=0.49). Insufficient data were available to carry out any of the planned subgroup analyses on the primary outcome measure. Concern about small trial effects caused us to carry out a sensitivity analysis. Removal of all chronic migraine trials at high risk of bias from study size left just the two PREEMT trials, which gave a more conservative reduction of 2.0 days per month (95% CI -2.8 to -1.1, N=1384).

INSERT FIGURE 2

Migraine severity score on a 10 cm visual analogue scale (VAS), improved by -3.3 cm (95% CI -4.2 to -2.4) more with active treatment (fig 3). Only four small trials reported meta-analyzable data for this outcome. For Chronic migraine the improvement was -2.7 cm (95% CI -3.3 to -2.1, N=75), and for episodic migraine it was -4.9 cm (95% CI -6.6 to -3.2, N=34).

INSERT FIGURE 3

A reduction in the number headache days per month of 1.9 days (95% CI -2.7 to -1.0, 2 trials, N = 1384) in favor of BTX type-A treatment was also seen. However data for number of migraine attacks from six trials of both chronic migraine and episodic migraine participants

For peer review only

(N = 2004) showed no significant between group difference (P = 0.30). Duration of migraine in hours was fully reported by only one trial showing a greater reduction of -5.1 hours (95% CI -6.2 to -4.0) for 102 chronic and episodic migraine participants. A further four trials with 420 participants reported no significant difference between groups for this outcome. Global assessment measures and quality of life measures were poorly reported and it was not possible to carry out statistical analysis of these outcome measures.

Effectiveness of BTX versus oral prophylactic agents

Three trials with 178 participants compared Botox® injections with oral prophylactic agents using double dummy techniques. Two trials compared 100 U fixed dose plus optional dose of up to 100 U of Botox® with topiramate maximum dose 200 mg/day.[42 43] The third trial compared treatment with up to 100 U Botox® with sodium valproate 250 mg twice daily.[41] Fourteen of the 178 participants had episodic migraine, all other participants had chronic migraine. Where meta-analysis was possible we pooled data from these three trials as there were insufficient data to allow us to explore comparisons with individual drug types or effects on chronic migraine and episodic migraine populations.

The primary outcome, number of migraine days per month was recorded in only one of the active comparison trials. The trialists reported that there was no statistically significant difference between treatment with BTX type-A and topiramate for this outcome.[43]

The number of headache days per month was recorded in two trials. No difference in number of headache days per month between treatment with BTX type-A and sodium valproate was reported (P=0.55).[35] No data were reported but it was stated that there was also no statistically significant difference between BTX type-A and topiramate treated groups.[42] A 5-point scale was used to compare the effect of BTX type-A with alternative agents in two trials, Blumenfeld et al reported no significant difference and Mathew et al reported within group analysis only.[41 43] Number of migraine attacks and duration of migraine were not

For peer review only

reported by any trial. No difference between BTX type-A and topiramate was stated for use of rescue medications.[43]

Of all the secondary outcome measures, data for meta-analysis were available only for the Migraine disability assessment (MIDAS) scores. Results of this showed no significant difference in change scores between the established drug treatments and injection with Botox® (P = 0.80, 2 trials, N = 101).

Safety

BTX type-A had an RR of treatment related adverse events of twice that seen for placebo (2.2, 95% CI 1.7 to 2.8, 6 trials, N=2839) (fig 4). All of these events were transient and non-serious, the most common being blepharoptosis, muscle weakness, injection site pain and neck pain.

INSERT FIGURE 4

Compared with oral treatments, BTX type-A showed a reduced RR of treatment related adverse events of 0.76 (95% CI 0.59 to 0.98, 2 trials, N=73). There was also difference in favor of BTX type-A in the RR of withdrawing due to adverse events of 0.28 (95% CI 0.10 to 0.79; I2 = 0%) which is a RR reduction of 72%.

A low withdrawal rate of 3% for BTX type-A was generated using data from all those trials treating with more than one injection cycle irrespective of the type of comparison arm.

Quality of the Evidence

The quality of the evidence assessed using GRADE methods was varied but mostly low and very low; the primary outcome measure was low and very low quality evidence for the placebo and active control comparisons respectively. Small trial size, high risk of bias and unexplained heterogeneity were common reasons for downgrading the quality of the evidence. All judgements and reasons for gradings are given in Supplemental files 3 and 4.

Discussion

For peer review only

Evidence was identified to support the use of injections of BTX type-A into the head and neck muscles, to reduce the number of migraine days experienced per month. Mean frequency of migraine days was significantly reduced by 3 days per month more by BTX type-A treatment than by placebo, but this result was revised to 2 days per month as a result of sensitivity analyses. All patients included in this analysis had chronic migraine and so had a high baseline frequency with an average of 20 days per month quoted by the two largest trials in the analysis.[6 7] For patients with chronic migraine, likely to be refractory to first and second line treatment, a 2-3 day improvement may well represent a meaningful difference. BTX type-A groups also fared better than placebo in the frequency of headache days by 2 days per month. Severity of migraine measured on a visual analogue scale was improved by 3 cm for chronic migraine and 5 cm for episodic migraine on a 10 cm scale. Though these results were from few small trials and the estimate is considered to be low quality evidence, the differences in severity scores were in excess of the minimal clinically important difference of 1.2 cm determined by Kelly *et al.*[45] and indicate that the treatment may be reducing the impact of each migraine attack. In contrast to this no significant difference from placebo was observed for frequency of migraine attacks. Patient and clinician reported global assessment scales and quality of life scales were underused and when they were incorporated into trials they were poorly reported, so no aggregation of data of this type comparing investigative treatment with placebo was possible in this review.

It was not possible to carry out any analysis on headache diary outcomes or severity measures for head-to-head comparisons between BTX type-A and other established agents due to lack of available data. MIDAS scores for 101 patients from two small trials, one comparing Botox® with topiramate and one with sodium valproate were available and these showed no significant between group difference (P=0.8).

For peer review only

Trials included in this review commonly state that BTX's have good safety profiles and the evidence from the 23 trials included in this review which reported adverse events in some form support those assertions. Although an increased risk of experiencing treatment related adverse events was found for the BTX type-A treated group compared with placebo, the event types were non-serious and transient.

A relative risk reduction (RRR) of 24% in treatment related adverse events in favor of BTX type-A was found when comparing with topiramate and sodium valproate in two trials. These two trials found an RRR in favor of BTX type-A of 72% for withdrawal rate due to adverse events. Percentage withdrawals due to adverse events for all of those trials included in this review which used more than one round of BTX type-A injections, irrespective of the comparison arm type, was 3%. The data sets for the direct comparisons with other prophylactic agents were small, but the relationship is supported by the indirect comparison of this percentage with published rates of 20% for topiramate and 12% for sodium valproate.[46 47] This result suggests that patients tolerate this treatment better than the oral alternatives.

Reporting was generally poor, with only six of 28 trials reporting data on our primary outcome in a usable format, and an additional five providing data for frequency of migraine attacks. These two outcomes are recommended as primary outcomes by the trial guidelines produced by the IHS and should be fully reported to allow individual trials to be placed in the context of the totality of the evidence.[48] A large proportion of the recorded data were missing from the published reports of our included trials. Failure to fully report data in trial publications led to problems throughout the meta-analysis and greater confidence in the conclusions would have been possible if all trials that recorded our outcomes of interest had fully reported them.

For peer review only

Prophylactic treatments for migraine aim to reduce the frequency, duration and/or the intensity of attacks. Frequency of migraine attacks was commonly used as the primary outcome particularly in studies carried out before the publication of the PREEMT trials. Use of this measure may mask an important improvement in symptoms seen in the form of shorter and less intense migraine attacks. Use of the more sensitive measures, number of days or hours spent with migraine per month coupled with a measure of intensity, may enable detection of such changes and could be particularly relevant to episodic migraine patients for whom attacks may be shorter at baseline. Another problem with focusing on this outcome measure was the failure generally to define what was meant by a migraine attack, and therefore, the likelihood of variation in the definitions used across the trials.

Neither efficacy nor safety data were available for long term treatment with BTX. The longest treatment period in any of the studies included in this review was three treatments with 12 weeks between treatments, so we cannot know the implications of treating patients with BTX over a period longer than 9 months.

Most trials did not report whether or not they had included patients with medication overuse symptoms and those that did stated they had largely excluded medication overuse patients. Pooled data for the two PREEMPT trials for the chronic migraine plus medication overuse subgroup (N=906) showed that the difference between groups for both migraine and headache day frequencies was 2 days (P<.001) in favor of treatment with BTX.[49] The medication overuse subgroup result falls within the confidence intervals of the pooled estimate generated by this review for the same outcome measure in combined populations with and without medication overuse headache. It would appear from these data that the inclusion of patients with medication overuse does not change the effectiveness of BTX for prophylactic treatment of migraine.

Conclusions

For peer review only

We have data which suggest that BTX effectively reduces the duration and severity of migraines in sufferers. There are however question marks over the quality of the evidence. Efficacy measures were commonly reported as showing non-inferiority of BTX to topiramate and sodium valproate and the withdrawal rate from BTX is much lower than that for first line prophylactic treatments for migraine. So should we be using more BTX?

It is currently recommended by NICE guidance that medication overuse headache should be addressed before treatment with BTX but trial data suggests it is efficacious in chronic migraine patients with untreated medication overuse headache. So although treatment of medication overuse headache is good practice, perhaps it should not be a requirement before prescription of BTX. NICE recommends the use of BTX to treat chronic migraine that has not responded to at least three prior pharmacological prophylaxis therapies. The confidence in the effectiveness of these drugs is arguably no greater than that for BTX and patients seem better able to tolerate BTX.[4 5 46 47] If, as is suggested by trial data, BTX has the equivalent efficacy to other agents but lower withdrawal rates, then if it were not for the higher cost, BTX would likely be recommended as an earlier preventative treatment for chronic migraine. The difference between chronic and episodic migraine diagnoses is arbitrary and so there is no pathophysiological reason that treatment with BTX would be efficacious in people with 15 days headache per month and inefficacious in people with 14 days of headache per month in a stepwise fashion. The treatment may well be useful for episodic migraine, particularly in high frequency episodic migraine, but data is lacking.

Acknowledgements

We would like to thank Joanne Abbott, Information Specialist at the Cochrane Pain, Palliative and Supportive Care Group, who ran searches in MEDLINE, Embase and

For peer review only - http://bmjopen.bmj.com/site/about/guidelines.xhtml

CENTRAL. We would also like to thank Ana Hughes, Cancer Clinical Trials Unit, University of Birmingham for her translation of Blumenkron 2006.

Author contributions

CEC, AS and JE conceived the review. CH ran searches not covered by the review group's information specialist. CH, CLT, CR and AS screened the search results. CH, CLT, CR, WS, CEC and AS assessed the quality of studies and extracted data. CH contacted trial authors, managed data and entered it into RevMan, and carried out data analysis. NI provided statistical advice. CH, CLT, CR, JE, NI, CEC and AS were involved in interpretation of the results. All authors read and edited final version of the review.

Funding

The authors received no financial support for the research, authorship, or publication of this article.

AJS is funded by an NIHR Clinician Scientist Fellowship (NIHR-CS-011-028), by the Medical Research Council, UK (MR/K015184/1)

Competing interests

JE received funding from Allergan in 2017 to attend a Master Class in Botulinum toxin. For all remaining authors none declared.

References

1. Collaborators GDaIIaP. Global, regional, and national incidence, prevalence, and years lived with disability for 310 diseases and injuries, 1990-2015: a systematic analysis for the Global Burden of Disease Study 2015. Lancet (London, England) 2016;**388**(10053):1545-602.
2. Linde M, Gustavsson A, Stovner LJ, et al. The cost of headache disorders in Europe: the Eurolight project. European Journal of Neurology 2012;**19**:703-11.

3. Linde K, Rossnagel K. Propranolol for migraine prophylaxis. Cochrane Database of Systematic Reviews 2004(2).
4. Linde M, Mulleners WM, Chronicle EP, et al. Gabapentin or pregabalin for the prophylaxis of episodic migraine in adults. Cochrane Database of Systematic Reviews 2013(6).
5. Linde M, Mulleners WM, Chronicle EP, et al. Antiepileptics other than gabapentin, pregabalin, topiramate, and valproate for the prophylaxis of episodic migraine in adults. Cochrane Database of Systematic Reviews 2013(6).
6. Aurora SK, Dodick DW, Turkel CC, et al. OnabotulinumtoxinA for treatment of chronic migraine: results from the double-blind, randomized, placebo-controlled phase of the PREEMPT 1 trial. Cephalalgia : an international journal of headache 2010;**30**(7):793-803.
7. Diener HC, Dodick DW, Aurora SK, et al. OnabotulinumtoxinA for treatment of chronic migraine: Results from the double-blind, randomized, placebo-controlled phase of the PREEMPT 2 trial. Cephalalgia 2010;**30**(7):804-14.
8. National Institute for Health Care Excellence. Botulinum toxin type A for the prevention of headaches in adults with chronic migraine. NICE technology appraisal guidance [TA260]. Published date: June 2012. Available from http://wwwniceorguk/guidance/ta260.
9. National Institute for Health Care Excellence. Headaches: Diagnosis and management of headaches in young people and adults. NICE guidelines [CG150]. Published date: September 2012. Available from http://wwwniceorguk/guidance/cg150.
10. British Medical Association, Society RP. British National Formulary (BNF). Available from wwwbnforg.
11. Headache Classification Committee of the International Headache S. The international classification of headache disorders, 3rd edition. Cephalalgia 2013;**33**(9):629-808.
12. Headache Classification Committee of the International Headache Society. The International Classification of Headache Disorders, 2nd edition, 1st revision. Cephalalgia 2004;**24**(Suppl 1):1-160.
13. Herd CP, Tomlinson CL, Rick C, et al. Botulinum toxins for the prevention of migraine in adults. Cochrane Database Syst Rev 2018;**6**:CD011616.
14. Headache Classification Committee of the International Headache Society. Classification and diagnostic criteria for headache disorders, cranial neuralgias and facial pain. Cephalalgia 1988;**8**(Suppl. 7):1-96.
15. Review Manager (RevMan) [program]. Version 5.3 version. Copenhagen: The Cochrane Collaboration, 2014.
16. DerSimonian R, Laird N. Meta-analysis in clinical trials. Controlled clinical trials 1986;**7**(3):177-88.
17. Dechartres A, Trinquart L, Boutron I, et al. Influence of trial sample size on treatment effect estimates: meta-epidemiological study. BMJ 2013;**346**:f2304-f04.
18. Nüesch E, Trelle S, Reichenbach S, et al. Small study effects in meta-analyses of osteoarthritis trials: Meta-epidemiological study. BMJ 2010;**341**:c3515-c15.
19. Guyatt GH, Oxman AD, Vist GE, et al. GRADE: an emerging consensus on rating quality of evidence and strength of recommendations. BMJ 2008;**336**(7650):924-26.
20. Anand KS, Prasad A, Singh MM, et al. Botulinum toxin type A in prophylactic treatment of migraine. American journal of therapeutics 2006;**13**(3):183-7.
21. Aurora SK, Gawel M, Brandes JL, et al. Botulinum toxin type a prophylactic treatment of episodic migraine: a randomized, double-blind, placebo-controlled exploratory study. Headache 2007;**47**(4):486-99.

For peer review only

22. Barrientos N, Chana P. Botulinum toxin type A in prophylactic treatment of migraine headaches: A preliminary study. Journal of headache and pain 2003;**4**(3):146-51.
23. Blumenkron D, Rivera C, Cuevas C. Efficacy of botulinum toxin type A in patients with migraine. [Spanish] Eficacia del tratamiento con toxina botulinica tipo A en pacientes con migrana. Medicina Interna de Mexico 2006;**22**(1):25-31.
24. Cady R, Schreiber C. Botulinum toxin type A as migraine preventive treatment in patients previously failing oral prophylactic treatment due to compliance issues. Headache 2008;**48**(6):900-13.
25. Chankrachang S, Arayawichanont A, Poungvarin N, et al. Prophylactic botulinum type A toxin complex (Dysport®) for migraine without aura. Headache 2011;**51**(1):52-63.
26. Elkind AH, O'Carroll P, Blumenfeld A, et al. A series of three sequential, randomized, controlled studies of repeated treatments with botulinum toxin type A for migraine prophylaxis. The journal of pain : official journal of the American Pain Society 2006;**7**(10):688-96.
27. Freitag FG, Diamond S, Diamond M, et al. Botulinum Toxin Type A in the treatment of chronic migraine without medication overuse. Headache 2008;**48**(2):201-9.
28. Hollanda L, Monteiro L, Melo AS. Botulinum toxin type A for cephalic cutaneous allodynia in chronic migraine: A randomized, double-blinded, placebo-controlled trial. Neurology international 2014;**6**(4):70-3.
29. Hou M, Xie JF, Kong XP, et al. Acupoint injection of onabotulinumtoxin a for migraines. Toxins 2015;**7**(11):4442-54.
30. Jost WH. Low-dosed botulinum toxin a in the prophylactic management of unilateral migraine: A randomized double-blind placebo-controlled crossover study. Open pain journal 2011;**4**(1):4-7.
31. Lauretti GR, Rosa CP, Kitayama A, et al. Comparison of Botox® or Prosigne® and Facial Nerve Blockade as Adjuvant in Chronic Migraine. Journal of Biomedical Science and Engineering 2014;**7**:446-52.
32. Petri S, Tölle T, Straube A, et al. Botulinum toxin as preventive treatment for migraine: a randomized double-blind study. European neurology 2009;**62**(4):204-11.
33. Relja M, Poole AC, Schoenen J, et al. A multicentre, double-blind, randomized, placebo-controlled, parallel group study of multiple treatments of botulinum toxin type A (BoNTA) for the prophylaxis of episodic migraine headaches. Cephalalgia : an international journal of headache 2007;**27**(6):492-503.
34. Saper JR, Mathew NT, Loder EW, et al. A double-blind, randomized, placebo-controlled comparison of botulinum toxin type a injection sites and doses in the prevention of episodic migraine. Pain medicine (Malden, Mass) 2007;**8**(6):478-85.
35. Silberstein S, Mathew N, Saper J, et al. Botulinum toxin type A as a migraine preventive treatment. Headache 2000;**40**(6):445-50.
36. Cady R, Turner I, Dexter K, et al. An exploratory study of salivary calcitonin gene-related peptide levels relative to acute interventions and preventative treatment with onabotulinumtoxinA in chronic migraine. Headache 2014;**54**(2):269-77.
37. Vo AH, Satori R, Jabbari B, et al. Botulinum toxin type-a in the prevention of migraine: a double-blind controlled trial. Aviation, space, and environmental medicine 2007;**78**(5 Suppl):B113-8.
38. Jabbari B. Investigation of Efficacy and Safety of Botulinum Toxin A (Botox-Allergan Inc) in Migraine Headaches. clinicaltrials.gov, 2008.
39. Mazza MR, Ferrigno G, Vescio B, et al. Subcutaneous botulinum toxin type a treatment for prophylaxis of headaches in chronic migraine: A new therapeutic strategy. Cephalalgia 2016;**36**:35.

For peer review only

40. Allergan. Use of a Treatment Benefit Questionnaire in Patients With Chronic Migraine Treated With OnabotulinumtoxinA (BOTOX®). clinicaltrials.gov, 2013.
41. Blumenfeld AM, Schim JD, Chippendale TJ. Botulinum toxin type A and divalproex sodium for prophylactic treatment of episodic or chronic migraine. Headache 2008;**48**(2):210-20.
42. Cady RK, Schreiber CP, Porter JA, et al. A multi-center double-blind pilot comparison of onabotulinumtoxinA and topiramate for the prophylactic treatment of chronic migraine. Headache 2011;**51**(1):21-32.
43. Mathew NT, Jaffri SF. A double-blind comparison of onabotulinumtoxina (BOTOX) and topiramate (TOPAMAX) for the prophylactic treatment of chronic migraine: a pilot study. Headache 2009;**49**(10):1466-78.
44. Millán-Guerrero RO, Isais-Millán S, Barreto-Vizcaíno S, et al. Subcutaneous histamine versus botulinum toxin type A in migraine prophylaxis: a randomized, double-blind study. European journal of neurology 2009;**16**(1):88-94.
45. Kelly AM. The minimum clinically significant difference in visual analogue scale pain score does not differ with severity of pain. Emerg Med J 2001;**18**:205–07.
46. Linde M, Mulleners WM, Chronicle EP, et al. Valproate (valproic acid or sodium valproate or a combination of the two) for the prophylaxis of episodic migraine in adults. Cochrane Database of Systematic Reviews 2013(6).
47. Linde M, Mulleners WM, Chronicle EP, et al. Topiramate for the prophylaxis of episodic migraine in adults. Cochrane Database of Systematic Reviews 2013(6).
48. Tfelt-Hansen P, Pascual J, Ramadan N, et al. Guidelines for controlled trials of drugs in migraine: third edition. A guide for investigators. Cephalalgia 2012;**32**(1):6-38.
49. Silberstein SD, Blumenfeld AM, Cady RK, et al. OnabotulinumtoxinA for treatment of chronic migraine: PREEMPT 24-week pooled subgroup analysis of patients who had acute headache medication overuse at baseline. Journal of the neurological sciences 2013;**331**(1-2):48-56.

Figure legends

Figure 1: Risk of bias graph: review authors' judgements about each risk of bias item presented as percentages across all included studies.

Figure 2: Comparison of BTX type-A versus placebo in relation to number of migraine days per month.

Figure 3: Comparison of BTX type-A versus placebo in relation to severity of migraine measured on a 10 cm visual analogue scale.

Figure 4: Comparison of BTX type-A versus placebo in relation to treatment related adverse events.

For peer review only

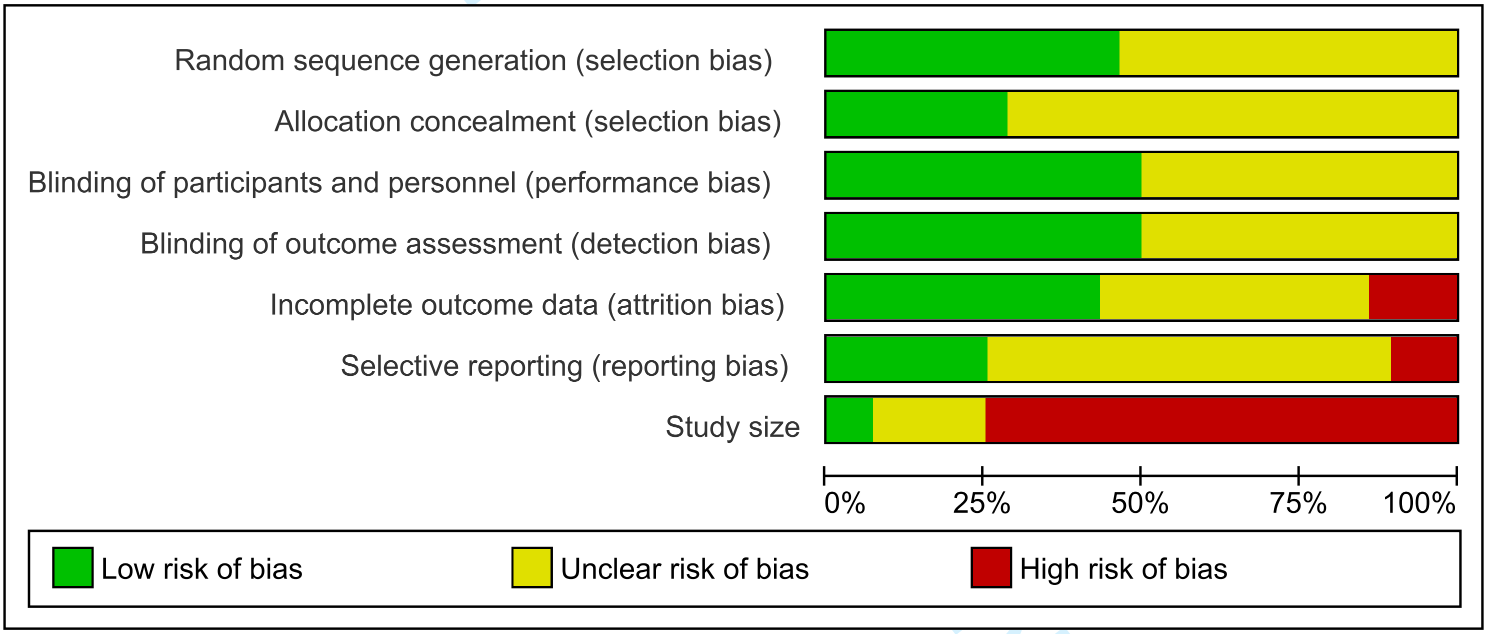

For peer review only - http://bmjopen.bmj.com/site/about/guidelines.xhtml

Study or Subgroup	Botulinum toxin Mean [Days]	SD [Days]	Total	Placebo Mean [Days]	SD [Days]	Total	Weight	Mean Difference IV, Random, 95% CI [Days]
**1.1.1 Chronic migraine**								
Aurora 2010 (PREEMPT 1)	-7.6	6.5	341	-6.1	6.8	338	24.3%	-1.50 [-2.50, -0.50]
Cady 2014	-10.1	5.9	9	-5.2	5.1	10	7.6%	-4.90 [-9.88, 0.08]
Diener 2010 (PREEMPT 2)	-8.7	6.6	347	-6.3	6.7	358	24.4%	-2.40 [-3.38, -1.42]
Mazza 2016	9.4	8.6	63	15.1	1.8	31	17.9%	-5.70 [-7.92, -3.48]
**Subtotal (95% CI)**			**760**			**737**	**74.1%**	**-3.07 [-4.73, -1.41]**
Heterogeneity: Tau² = 1.84; Chi² = 12.56, df = 3 (P = 0.006); I² = 76%								
Test for overall effect: Z = 3.62 (P = 0.0003)								
**1.1.2 Episodic migraine**								
Elkind I 2006	-1.4	2.5	312	-1.2	2.6	106	25.9%	-0.20 [-0.77, 0.37]
**Subtotal (95% CI)**			**312**			**106**	**25.9%**	**-0.20 [-0.77, 0.37]**
Heterogeneity: Not applicable								
Test for overall effect: Z = 0.69 (P = 0.49)								
**Total (95% CI)**			**1072**			**843**	**100.0%**	**-2.39 [-4.02, -0.76]**
Heterogeneity: Tau² = 2.59; Chi² = 35.80, df = 4 (P < 0.00001); I² = 89%								
Test for overall effect: Z = 2.88 (P = 0.004)								
Test for subgroup differences: Chi² = 10.26, df = 1 (P = 0.001), I² = 90.3%								

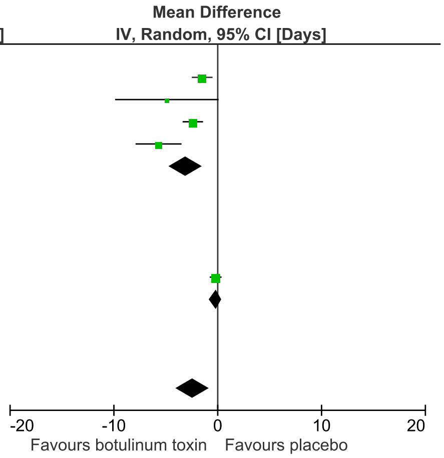

For peer review only

Study or Subgroup	Botulinum toxin Mean	SD	Total	Placebo Mean	SD	Total	Weight	Mean Difference IV, Random, 95% CI
**1.4.1 Chronic migraine**								
Hollanda 2014	3.45	2.39	20	6.17	2.5	18	18.3%	-2.72 [-4.28, -1.16]
Lauretti 2014	6.5	1.3	28	9.2	0.7	9	36.6%	-2.70 [-3.36, -2.04]
**Subtotal (95% CI)**			**48**			**27**	**54.9%**	**-2.70 [-3.31, -2.09]**
Heterogeneity: Tau² = 0.00; Chi² = 0.00, df = 1 (P = 0.98); I² = 0%								
Test for overall effect: Z = 8.67 (P < 0.00001)								
**1.4.2 Episodic migraine**								
Anand 2006	2.4	3.2	16	7.3	1.1	16	17.0%	-4.90 [-6.56, -3.24]
**Subtotal (95% CI)**			**16**			**16**	**17.0%**	**-4.90 [-6.56, -3.24]**
Heterogeneity: Not applicable								
Test for overall effect: Z = 5.79 (P < 0.00001)								
**1.4.3 Mixed**								
Hou 2015	2.8	1.2	83	6.3	2.2	19	28.1%	-3.50 [-4.52, -2.48]
**Subtotal (95% CI)**			**83**			**19**	**28.1%**	**-3.50 [-4.52, -2.48]**
Heterogeneity: Not applicable								
Test for overall effect: Z = 6.71 (P < 0.00001)								
**Total (95% CI)**			**147**			**62**	**100.0%**	**-3.30 [-4.16, -2.45]**
Heterogeneity: Tau² = 0.40; Chi² = 6.74, df = 3 (P = 0.08); I² = 55%								
Test for overall effect: Z = 7.58 (P < 0.00001)								
Test for subgroup differences: Chi² = 6.74, df = 2 (P = 0.03), I² = 70.3%								

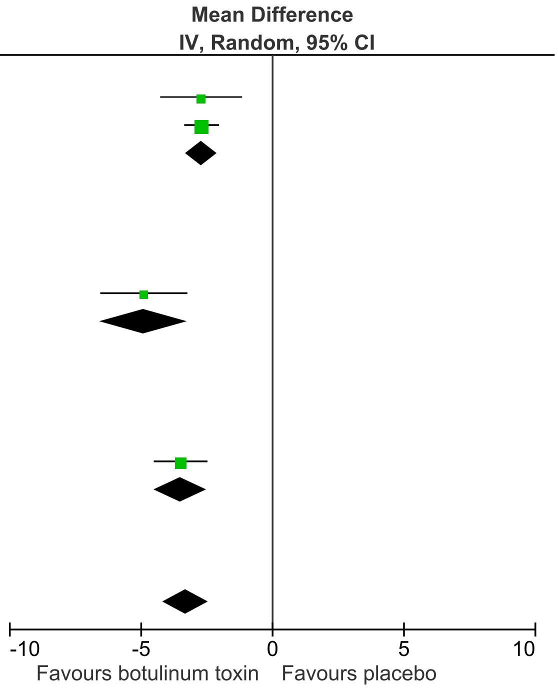

	Botulinum toxin		Placebo			Risk Ratio
**Study or Subgroup**	**Events**	**Total**	**Events**	**Total**	**Weight**	**M-H, Random, 95% CI**
**1.11.1 Chronic migraine**						
Aurora 2010 (PREEMPT 1)	86	340	39	334	18.6%	2.17 [1.53, 3.06]
Diener 2010 (PREEMPT 2)	116	347	49	358	20.8%	2.44 [1.81, 3.30]
**Subtotal (95% CI)**		**687**		**692**	**39.3%**	**2.32 [1.85, 2.91]**
Total events	202		88			
Heterogeneity: Tau² = 0.00; Chi² = 0.26, df = 1 (P = 0.61); I² = 0%						
Test for overall effect: Z = 7.28 (P < 0.00001)						
**1.11.2 Episodic migraine**						
Aurora 2007	113	187	39	182	20.7%	2.82 [2.09, 3.81]
Elkind I 2006	61	312	7	106	7.3%	2.96 [1.40, 6.27]
Relja 2007	243	377	37	118	21.9%	2.06 [1.56, 2.71]
Saper 2007	45	187	11	45	10.8%	0.98 [0.55, 1.75]
**Subtotal (95% CI)**		**1063**		**451**	**60.7%**	**2.06 [1.37, 3.08]**
Total events	462		94			
Heterogeneity: Tau² = 0.11; Chi² = 11.01, df = 3 (P = 0.01); I² = 73%						
Test for overall effect: Z = 3.50 (P = 0.0005)						
**Total (95% CI)**		**1750**		**1143**	**100.0%**	**2.18 [1.73, 2.75]**
Total events	664		182			
Heterogeneity: Tau² = 0.04; Chi² = 11.41, df = 5 (P = 0.04); I² = 56%						
Test for overall effect: Z = 6.59 (P < 0.00001)						
Test for subgroup differences: Chi² = 0.26, df = 1 (P = 0.61), I² = 0%						

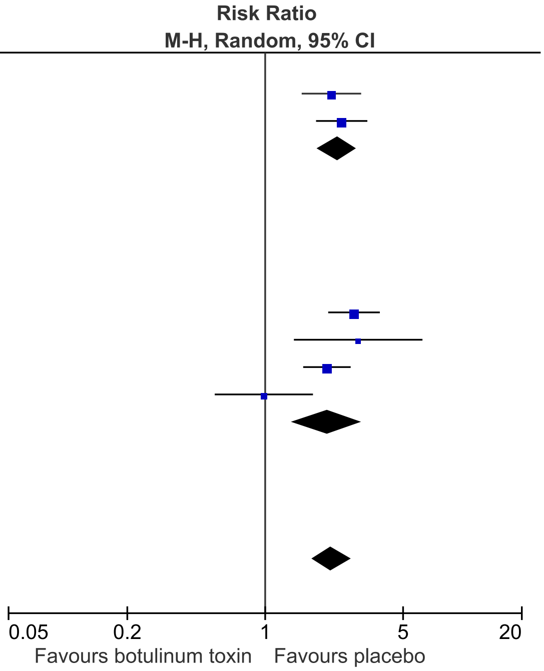

MEDLINE (via OVID)

#1 Exp headache disorders/

#2 headache/

#3 (headache* or migrain* or cephalgi* or cephalalgi* or hemicrani*).mp.

#4 or/1-3

#5 exp botulinum toxins/

#6 (botulin* adj toxin*).tw

#7 (botulinum* or oculinu* or boto* or onabotulinum*).tw.

#8 exp botulinum toxin type A/

#9 Exp clostridium botulinum/

#10 clostridium botulin*.tw.

#11 or/5-10

12 randomized controlled trial.pt.

13 controlled clinical trial.pt.

14 randomized.ab.

15 placebo.ab.

16 drug therapy.fs.

17 randomly.ab.

18 trial.ab.

19 groups.ab.

20 12 or 13 or 14 or 15 or 16 or 17 or 18 or 19

21 exp animals/ not humans.sh.

22 20 not 21

23 4 and 11 and 22

For peer review only

PRISMA 2009 Flow Diagram

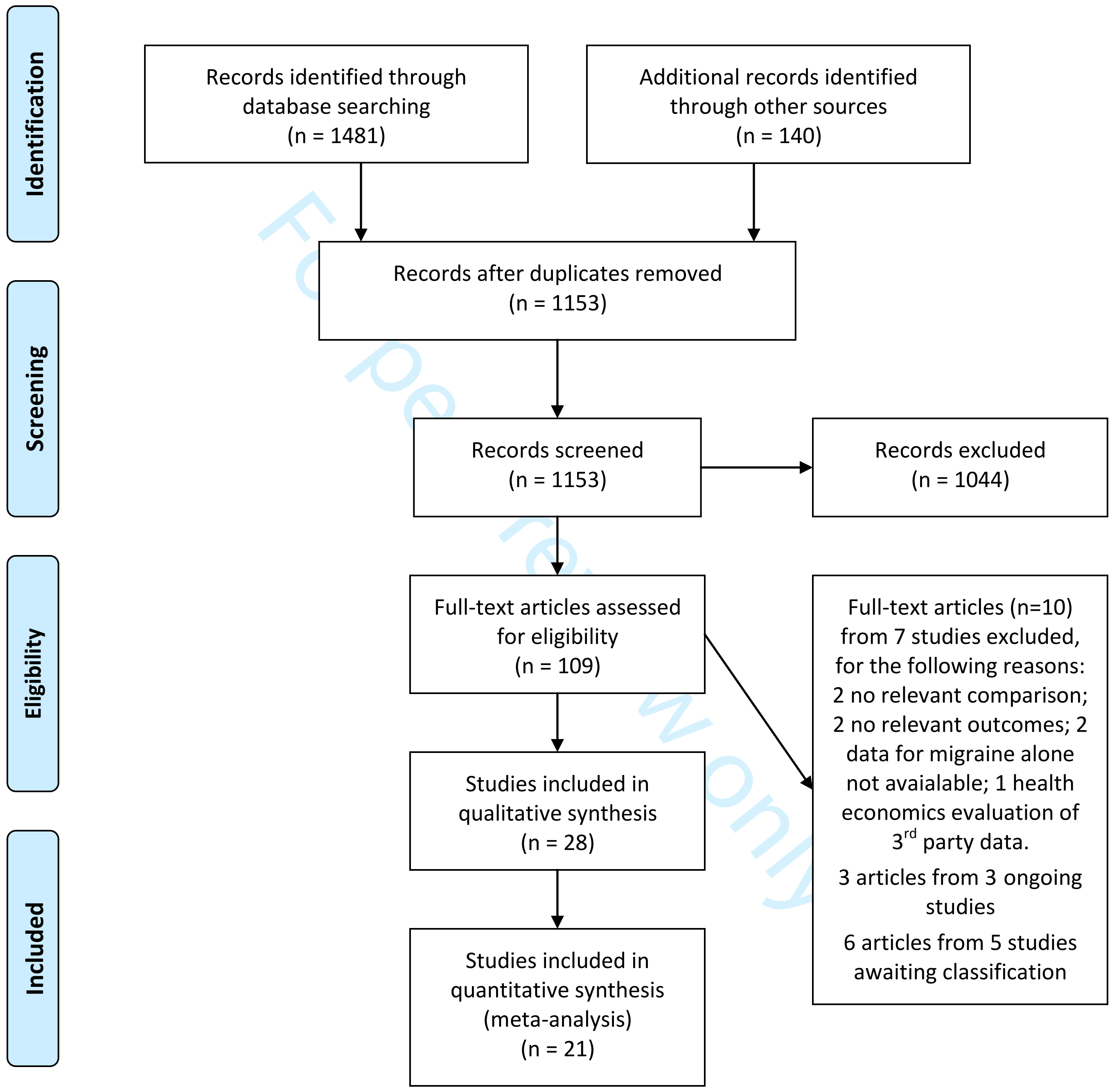

From: Moher D, Liberati A, Tetzlaff J, Altman DG, The PRISMA Group (2009). *P*referred *R*eporting *I*tems for *S*ystematic Reviews and *M*eta-*A*nalyses: The PRISMA Statement. PLoS Med 6(7): e1000097. doi:10.1371/journal.pmed1000097

For more information, visit www.prisma-statement.org.

For peer review only - http://bmjopen.bmj.com/site/about/guidelines.xhtml

BTX-A compared to placebo for the prevention of migraine in adults

Outcomes	Result with BTX-A (95% CI)	Number of participants (studies)	Quality of the evidence (GRADE)
Number of migraine days per month - Chronic migraine	MD 3.1 days lower (4.7 lower to 1.4 lower)	1497 (4 RCTs)	⊕⊕⊖⊖ LOW [a b]
Number of headache days per month - Chronic migraine	MD 1.9 days lower (2.7 lower to 1.0 lower)	1384 (2 RCTs)	⊕⊕⊕⊕ HIGH
Number of migraine attacks	MD 0.5 attacks lower (1.3 lower to 0.4 higher)	2004 (6 RCTs)	⊕⊕⊖⊖ LOW [c d]
Headache intensity measure - Chronic migraine (Visual Analogue Score 0-10)	MD 2.7 cm lower (3.3 lower to 2.1 lower)	75 (2 RCTs)	⊕⊖⊖⊖ VERY LOW [e f]
Headache intensity measure - Episodic migraine (Visual Analogue Score 0-10)	MD 4.9 cm lower (6.6 lower to 3.2 lower)	75 (1 RCT)	⊕⊖⊖⊖ VERY LOW [e f]
Headache Impact Test-6	MD 1.6 points higher (2.1 lower to 5.3 higher)	45 (1 RCT)	⊕⊖⊖⊖ VERY LOW [e f]
Total number of participants experiencing an adverse event	RR 1.28 (1.1 to 1.5)	3325 (13 RCTs)	⊕⊕⊕⊖ MODERATE [g]

Footnotes

CI: Confidence interval; RR: Risk ratio; MD: Mean difference. [a] Downgraded once due to inconsistency: Statistical heterogeneity observed despite similarities in populations and doses. [b] Downgraded once due to imprecision: Sensitivity analysis testing robustness of result suggested small studies may be over estimating treatment effect. [c] Downgraded once due to indirectness: Sensitivity of this outcome measure at risk of being too low to detect clinically

For peer review only

meaningful differences. [d] Downgraded once due to publication bias: Evidence found of trials that have never been published which record this outcome. [e] Downgraded once due to risk of bias: High or unclear risk of selective reporting bias and poor reporting of this outcome measure had a large effect on numbers analyzed. [f] Downgraded twice due to imprecision: Study size small, new trial evidence likely to change result. [g] Downgraded once due to imprecision: Study size small, new trial evidence likely to change result. GRADE Working Group grades of evidence- High quality: We are very confident that the true effect lies close to that of the estimate of the effect; Moderate quality: We are moderately confident in the effect estimate: The true effect is likely to be close to the estimate of the effect, but there is a possibility that it is substantially different; Low quality: Our confidence in the effect estimate is limited: The true effect may be substantially different from the estimate of the effect; Very low quality: We have very little confidence in the effect estimate: The true effect is likely to be substantially different from the estimate of effect

For peer review only

BTX-A compared to other established prophylactic agent for the prevention of migraine in adults

Outcomes	Result with BTX-A (95% CI)	Number of participants (studies)	Quality of the evidence (GRADE)
Number of migraine days per month - chronic migraine	One trial using topiramate in its comparison arm reported narratively on this outcome stating that there was no significant difference between groups.	43 (1 RCT)	⊕⊖⊖⊖ VERY LOW [a b c]
Number of headache days per month	MD 1 days lower (4.3 lower to 2.3 higher)	59 (1 RCT)	⊕⊖⊖⊖ VERY LOW [a b]
Headache intensity measure assessed with: 5-point scale, 5 being severe, 1 being mild - chronic migraine only	MD 0.4 points lower (0.79 lower to 0.01 lower)	46 (1 RCT)	⊕⊖⊖⊖ VERY LOW [a b]
Global impression of disease assessed with: Migraine impact and disability assessment scores	MD 4.3 points higher (28 lower to 37 higher)	101 (2 RCTs)	⊕⊖⊖⊖ VERY LOW [a b]
Total number of participants experiencing an adverse event	RR 0.8 (0.4 to 1.9)	114 (2 RCTs)	⊕⊖⊖⊖ VERY LOW [a b]

Footnotes

CI: Confidence interval; RR: Risk ratio; MD: Mean difference. [a] Downgraded once due to risk of bias: Unclear or high risk for selection, performance, detection and attrition bias. [b] Downgraded twice due to imprecision: Study sizes small, new trial evidence likely to change result. [c] Downgraded once due to imprecision: Narrative description only. GRADE

For peer review only

Working Group grades of evidence- High quality: We are very confident that the true effect lies close to that of the estimate of the effect; Moderate quality: We are moderately confident in the effect estimate: The true effect is likely to be close to the estimate of the effect, but there is a possibility that it is substantially different; Low quality: Our confidence in the effect estimate is limited: The true effect may be substantially different from the estimate of the effect; Very low quality: We have very little confidence in the effect estimate: The true effect is likely to be substantially different from the estimate of effect.

For peer review only

PRISMA 2009 Checklist

Section/topic	#	Checklist item	Reported on page #
**TITLE**			
Title	1	Identify the report as a systematic review, meta-analysis, or both.	1
**ABSTRACT**			
Structured summary	2	Provide a structured summary including, as applicable: background; objectives; data sources; study eligibility criteria, participants, and interventions; study appraisal and synthesis methods; results; limitations; conclusions and implications of key findings; systematic review registration number.	1-2
**INTRODUCTION**			
Rationale	3	Describe the rationale for the review in the context of what is already known.	2-3
Objectives	4	Provide an explicit statement of questions being addressed with reference to participants, interventions, comparisons, outcomes, and study design (PICOS).	3
**METHODS**			
Protocol and registration	5	Indicate if a review protocol exists, if and where it can be accessed (e.g., Web address), and, if available, provide registration information including registration number.	3
Eligibility criteria	6	Specify study characteristics (e.g., PICOS, length of follow-up) and report characteristics (e.g., years considered, language, publication status) used as criteria for eligibility, giving rationale.	4
Information sources	7	Describe all information sources (e.g., databases with dates of coverage, contact with study authors to identify additional studies) in the search and date last searched.	3-4
Search	8	Present full electronic search strategy for at least one database, including any limits used, such that it could be repeated.	Suppl.file 1
Study selection	9	State the process for selecting studies (i.e., screening, eligibility, included in systematic review, and, if applicable, included in the meta-analysis).	4
Data collection process	10	Describe method of data extraction from reports (e.g., piloted forms, independently, in duplicate) and any processes for obtaining and confirming data from investigators.	5
Data items	11	List and define all variables for which data were sought (e.g., PICOS, funding sources) and any assumptions and simplifications made.	5
Risk of bias in individual studies	12	Describe methods used for assessing risk of bias of individual studies (including specification of whether this was done at the study or outcome level), and how this information is to be used in any data synthesis.	5
Summary measures	13	State the principal summary measures (e.g., risk ratio, difference in means).	5
Synthesis of results	14	Describe the methods of handling data and combining results of studies, if done, including measures of consistency (e.g., I^2) for each meta-analysis.	5

For peer review only - http://bmjopen.bmj.com/site/about/guidelines.xhtml

PRISMA 2009 Checklist

Section/topic	#	Checklist item	Reported on page #
Risk of bias across studies	15	Specify any assessment of risk of bias that may affect the cumulative evidence (e.g., publication bias, selective reporting within studies).	6
Additional analyses	16	Describe methods of additional analyses (e.g., sensitivity or subgroup analyses, meta-regression), if done, indicating which were pre-specified.	6
**RESULTS**			
Study selection	17	Give numbers of studies screened, assessed for eligibility, and included in the review, with reasons for exclusions at each stage, ideally with a flow diagram.	6 + suppl.file2
Study characteristics	18	For each study, present characteristics for which data were extracted (e.g., study size, PICOS, follow-up period) and provide the citations.	7
Risk of bias within studies	19	Present data on risk of bias of each study and, if available, any outcome level assessment (see item 12).	7
Results of individual studies	20	For all outcomes considered (benefits or harms), present, for each study: (a) simple summary data for each intervention group (b) effect estimates and confidence intervals, ideally with a forest plot.	8-10
Synthesis of results	21	Present results of each meta-analysis done, including confidence intervals and measures of consistency.	8-10
Risk of bias across studies	22	Present results of any assessment of risk of bias across studies (see Item 15).	11+suppl. Files 3&4
Additional analysis	23	Give results of additional analyses, if done (e.g., sensitivity or subgroup analyses, meta-regression [see Item 16]).	8
**DISCUSSION**			
Summary of evidence	24	Summarize the main findings including the strength of evidence for each main outcome; consider their relevance to key groups (e.g., healthcare providers, users, and policy makers).	11
Limitations	25	Discuss limitations at study and outcome level (e.g., risk of bias), and at review-level (e.g., incomplete retrieval of identified research, reporting bias).	11-13
Conclusions	26	Provide a general interpretation of the results in the context of other evidence, and implications for future research.	14
**FUNDING**			
Funding	27	Describe sources of funding for the systematic review and other support (e.g., supply of data); role of funders for the systematic review.	15

From: Moher D, Liberati A, Tetzlaff J, Altman DG, The PRISMA Group (2009). Preferred Reporting Items for Systematic Reviews and Meta-Analyses: The PRISMA Statement. PLoS Med 6(7): e1000097. doi:10.1371/journal.pmed1000097

For more information, visit: **www.prisma-statement.org**.

For peer review only - http://bmjopen.bmj.com/site/about/guidelines.xhtml

BMJ Open

Cochrane systematic review and meta-analysis of Botulinum toxin for the prevention of migraine.

Journal:	*BMJ Open*
Manuscript ID	bmjopen-2018-027953.R2
Article Type:	Research
Date Submitted by the Author:	14-Jun-2019
Complete List of Authors:	Herd, Clare; University of Birmingham, Institute of Applied Health Research<br>Tomlinson, Claire; University of Birmingham, BCTU<br>Rick, Caroline; University of Nottingham, Nottingham Clinical Trials Unit<br>Scotton, William; University of Birmingham, Institute of Metabolism and Systems Research<br>Edwards, Julie; Sandwell and West Birmingham Hospitals NHS Trust, Department of Neurology,<br>Ives, Natalie; University of Birmingham, BCTU<br>Clarke, Carl; University of Birmingham, Neurology<br>Sinclair, AJ; University of Birmingham, Institute of Metabolism and Systems Research
**Primary Subject Heading**:	Neurology
Secondary Subject Heading:	Medical management
Keywords:	Migraine Disorders, Botulinum toxin, Botox, Systematic Review, Randomised Controlled Trial

SCHOLARONE™
Manuscripts

Cochrane systematic review and meta-analysis of Botulinum toxin for the prevention of migraine.

Clare P Herd[1], Claire L Tomlinson[2], Caroline Rick[3], William J Scotton[4], Julie Edwards[5], Natalie Ives[2], Carl E Clarke[1,5], Alexandra Sinclair[4]

[1]Institute of Applied Health Research, University of Birmingham, Birmingham, UK.

[2]Birmingham Clinical Trials Unit, University of Birmingham, Birmingham, UK.

[3]Nottingham Clinical Trials Unit, University of Nottingham, Nottingham, UK

[4]Institute of Metabolism and Systems Research, University of Birmingham, Birmingham, UK

[5]Department of Neurology, Sandwell and West Birmingham Hospitals NHS Trust, Birmingham, UK

C.P.Herd@bham.ac.uk; C.L.Smith.1@bham.ac.uk; C.E.Rick@bham.ac.uk; W.Scotton@bham.ac.uk; julie.edwards14@nhs.net; N.J.Ives@bham.ac.uk; C.E.Clarke@bham.ac.uk;

Corresponding Author: A.B.Sinclair@bham.ac.uk

Word count: 4,343 (inclusive of abstract)

Keywords: Migraine Disorders, Botulinum toxin, Botox, Systematic Review, Randomised Controlled Trial

Data sharing statement

All data relevant to the study are included in this article, uploaded as supplementary information, or available in Cochrane review DOI: 10.1002/14651858.CD011616.pub2. No additional unpublished data was obtained from trial authors beyond what has been included in the Cochrane review.

For peer review only

Abstract

Objectives

To assess the effects of botulinum toxin for prevention of migraine in adults.

Design

Systematic review and meta-analysis.

Data sources

CENTRAL, MEDLINE, EMBASE and trial registries.

Eligibility criteria

We included randomized controlled trials (RCT's) of Botulinum toxin compared with placebo, active treatment or clinically relevant different dose for adults with chronic or episodic migraine, with or without the additional diagnosis of medication overuse headache. Data extraction and synthesis Cochrane methods were used to review double-blind RCT's. Twelve week post-treatment time-point data was analyzed.

Results

Twenty-eight trials (N=4190) were included. Trial quality was mixed. Botulinum toxin treatment resulted in reduced frequency of -2.0 migraine days/month (95% confidence interval -2.8 to -1.1, N=1384) in chronic migraineurs compared with placebo. An improvement was seen in migraine severity, measured on a numerical rating scale 0-10 with 10 being maximal pain, of -2.70 cm (95% confidence interval -3.31 to -2.09, N=75) and -4.9 cm (95% confidence interval -6.56 to -3.24, N=32) for chronic and episodic migraine respectively. Botulinum toxin had a relative risk of treatment related adverse events twice that of placebo, but a reduced risk compared to active comparators (relative risk 0.76, 95% confidence interval 0.59 to 0.98) and a low withdrawal rate (3%). Although individual trials reported non-inferiority to oral treatments, insufficient data were available for meta-analysis of effectiveness outcomes.

For peer review only

Conclusions

In chronic migraine, botulinum toxin reduces migraine frequency by three days/month and has a favorable safety profile. Inclusion of medication overuse headache does not preclude its effectiveness. Evidence to support or refute efficacy in episodic migraine was not identified.

Strengths and limitations

- This paper is a summary of a Cochrane review conducted using systematic and thorough methodology to identify and synthesize all available evidence for the effectiveness of botulinum toxin for prophylactic treatment of migraine.
- No language or date restrictions were placed on the search strategy.
- Many of the included studies were small in size and failed to fully report their data which impacted the quality ratings and the content of the meta-analyses.
- Our chosen primary outcome measure, though recommended in current guidelines for controlled trials of prophylactic treatment of chronic migraine, was not commonly recorded.

For peer review only

Introduction

Migraine is the seventh leading cause of years lived with disability globally and is estimated to affect around 15% of the world's population.[1] Days lost from work and other activities of daily living resulting from migraines have a major economic impact.[2] Many people with migraine suffer prolonged and frequent migraine attacks despite optimised acute and prophylactic treatments.[3-5]

Botulinum toxin type A (BTX-A) has been licensed for use in migraine in some countries, based largely on two commercially sponsored trials.[6 7] The recommended reconstituted dose is 155–195 units, administered intramuscularly as 0.1 ml (5 units) injections to between 31 and 39 sites around the head and neck.[3] Cost of treatment and administration of BTX-A is much higher than standard doses of the two first line treatments for the prevention of migraine, propranolol and topiramate (around 25 times and 15 times respectively in the UK).[8-10]

Migraine can be categorized as chronic or episodic and these terms are commonly used in eligibility criteria for clinical trials and systematic reviews. Chronic migraine is currently defined by the International Headache Society (IHS) as headache for at least 15 days per month with migraine features on eight of those days.[11] Episodic migraine is commonly used to describe patients with symptoms of migraine who have less than 15 headache days per month and according to official guidance is a term which can be used for migraine that is not covered by the definition of chronic migraine.[11] Migraine can occur with medication overuse headache; the IHS definition has evolved, but currently this is defined as an interaction between a therapeutic agent used excessively and a susceptible patient.[11 12] Trials recruiting participants with chronic migraine will come across many patients with this dual diagnosis. Current UK NICE guidelines recommend the use of BTX-A for chronic migraine, but not for

For peer review only

high frequency episodic migraine, and only when the condition is 'appropriately managed' for medication overuse.[8]

The aim of this evidence review was to assess the effects of botulinum toxin (BTX) versus placebo or alternative active treatment for the prophylaxis of episodic migraine or chronic migraine in adults.

This paper is a summary of key aspects from a Cochrane review first published in The Cochrane Library 2018, Issue 6 (see http://www.thecochranelibrary.com/ for information).[13] Cochrane reviews are regularly updated as new evidence emerges and in response to feedback, and The Cochrane Library should be consulted for the most recent version of the review.

Methods

The protocol for this review was published in the Cochrane Database of Systematic Reviews in advance of the publication of the full review which replaced it. Deviations from the protocol are listed in the full review.[13]

Search strategy

A systematic search of the literature published before March 2019 was carried out. We designed a highly sensitive search strategy using methods recommended by the Cochrane collaboration to minimize publication bias. No date, language or publication status restrictions were applied. We used a combination of index terms and free text terms for headache, migraine, cephalalgia or hemicrania; and botulinum toxin, botox, onabotulinum toxin, oculinum or clostridium botulinum. Relevant trials were identified through electronic searches of Cochrane Central Register of Controlled Trials, Medline (see full strategy in supplemental file 1), Embase, clinicaltrials.gov and World Health Organization International clinical trials registry, hand-searching reference lists and citation searches on key

For peer review only

publications, and correspondence with all major manufacturers of BTX products relevant to this review.

Selection criteria

We included randomized, double-blind, controlled trials of people over the age of 18 years suffering from migraine as defined by any edition of the IHS criteria,[11 12 14] or meeting reasonable criteria designed to distinguish between migraine and tension-type headache. Patients with both chronic migraine and episodic migraine were included in this review. Medication overuse headache was included as these types of participants have been included in large and prominent trials in this area. Trials must compare BTX (any sero-type) injected into the head and neck muscles with placebo injections, clinically relevant different dose of same treatment or active preventative agent. Trials allowing the use of concomitant preventative or rescue treatments were included.

Screening of abstracts and assessment of eligibility of full papers were carried out independently in duplicate and according to criteria predefined in the peer reviewed protocol. If disagreements occurred at any stage, a third author considered the available information or if necessary the study authors were contacted for clarification. When eligibility could not be determined through consideration of published materials or contact with trial authors the studies were excluded.

Quality assessment

Eligible material was assessed, independently by two reviewers for each trial, for methodological quality using Cochrane risk of bias methods. Publications were assessed on their method of randomization, blinding and concealment of allocation, the number of participants lost to follow-up, evidence of selective reporting and study size.

We considered the use of funnel plots to assess the risk of publication bias but did not carry them out. We made this decision because of the small number of studies included in the

For peer review only

individual meta-analyses and the true heterogeneity in the trial design (dose, injection paradigm) and populations studied (migraine sub-classifications), which would make it impossible to draw useful conclusions from the plots. GRADE tables were created for each comparison, this process involves assessment of the risk of publication bias for each outcome measure.

Data extraction

Data extraction was carried out independently and in duplicate onto forms designed and tested at protocol stage. The primary outcome was frequency of migraine days per month. Secondary outcomes included: frequency of headache days, frequency of migraine attacks, severity of migraine, duration of migraine, 50% responder rate, global impression scales, quality of life measures and adverse event reporting. We used risk ratios (RRs) as the preferred statistical output for dichotomous outcomes, with 95% confidence intervals (CIs). For continuous data, we used mean differences (MDs) with 95% CIs. Results with p values lower than 0.05 were considered to be statistically significant. Twelve week time-point data following final round of treatment was analyzed. We sought data from the first phase for any cross-over trials identified. We attempted to contact authors and obtain missing data.

Statistical analysis

The review authors assessed trial information and baseline characteristics to identify clinical and methodological differences during the data extraction process. If clinical and methodological homogeneity were confirmed, we carried out meta-analysis of the data using Review Manager (RevMan) 5.3.[15]

Heterogeneity present in doses, injection sites and participant populations led to the decision that a random-effects model should be used for the analysis. RevMan implements a version of random-effects meta-analysis that is described by Dersimonian and Laird[16] and presents an estimate of the between-study variance (Tau^2) at the bottom of each forest plot. We tested for

For peer review only

statistical homogeneity of pooled estimates of effectiveness using the Chi^2 test and the I^2 statistic, for which a statistically significant (P value ≤0.1) value of the Chi^2 test together with I^2 value of at least 50% indicates heterogeneity.

Within our eligible comparisons, we split data into migraine classification subgroups in order to show results for chronic migraine, episodic migraine and a mixed group for which the diagnosis could not be split.

We planned to use the following subgroups to test for variation in the effects of the intervention:

1. Trials including medication overuse headache versus trials excluding this type of patient.
2. Different sero-types of BTX (e.g. A versus B) and within sero-types (Dysport® versus Botox®).
3. Different types of agents for the prevention of migraine versus BTX.
4. Accepted and licensed 31 injection pattern versus other injection patterns used.

At least two trials and 200 participants per group were required for any particular subgroup analysis to be carried out.

We carried out sensitivity analyses for our primary outcome only. Prevailing evidence suggests that smaller trials are more likely to report stronger effect estimates than large trials.[17 18] To assess whether these stronger effect estimates reflected the true treatment effect we carried out a sensitivity analysis in which we examined the effect of removing studies at high risk of bias from study size.

We assessed the validity of our findings as well as the level of confidence suitable to any estimates of effect generated by our analyses using the Grading of Recommendations Assessment, Development and Evaluation (GRADE) approach.[19]

Patient and Public Involvement

For peer review only

There was no patient or public involvement in the design or reviewing process. However, the final Cochrane manuscript including a lay summary, which is accessible to the public through the Cochrane library, was reviewed by a patient representative as part of the editorial process. Their feedback was incorporated into the final draft.

Results

Description of included studies

The flow of information through the review process is given in the PRISMA flow chart in supplemental file 2. The characteristics of studies included in this review are given in supplemental file 3.

We identified 28 eligible trials, involving a total of 4190 participants, which were eligible for inclusion in this review. Twenty-three of these trials compared BTX type-A with placebo injections [6 7 20-40] and three compared with an alternative established oral prophylactic agent.[41-43]

Five trials, reported in four articles, compared alternative doses of BTX type-A,[26 33-35] all but one of these also included a placebo arm[26] and one compared with injections of histamine.[44] Due to the paucity of the data, review of the dosing studies and the histamine study are included as appendices in the Cochrane review and is not repeated here.[13]

The results of the critical appraisal were mixed (fig 1). Across all domains poor reporting was an issue and in all but attrition bias and study size at least 50% of trials provided insufficient information to allow judgments about risk of bias to be made. Only two trials were at low risk of bias due to study size (at least 200 participants per trial arm) and these two trials were also at low risk of bias across all other domains.[6 7]

INSERT FIGURE 1

For peer review only

Sixteen trials were commercially sponsored, including the only two trials at low risk from study size.[6 7 21 22 24-27 32-35 40 41 43]

For those trials providing information on the migraine diagnosis of their participants the ratio of chronic/episodic migraine was 1872/1928, leaving 392 included participants unclassified and analyzed as 'Mixed'. The mean age was 42 years and 85% of all participants were female. Pregnant women were generally explicitly excluded. All included trials used BTX type-A, of these 21 had at least one arm treated with the Botox® formulation,[6 7 20-24 26 27 30 31 33-35 38 40-44] two used Dysport®,[25 32] two used Prosigne®,[28 31] and one HengLi®.[29] The range of doses administered in trials of Botox® was 6 U to 300 U. The trials using Dysport® administered doses of 80 U up to 240 U in treated arms (dose equivalency reported by trial publications: 2 to 3 U:1 U Botox®). HengLi® and Prosigne® trials used doses ranging from 25 U to 96 U (dose equivalency reported by trial publications: 1 U:1 U Botox®).

Effectiveness versus placebo

Comparison with a placebo group was made in 23 trials with 3912 participants.

Meta-analysis of our primary outcome for the four trials in chronic migraine which reported it showed that there was a reduction of 3.1 days of migraine per month (95% CI -4.7 to -1.4) in favor of BTX type-A treatment (fig 2). At least 60% of the participants in this analysis had medication overuse headache. The episodic migraine subgroup involved only one trial of 418 participants which showed no difference in the number of migraine days between treated and placebo groups (P=0.49). Insufficient data were available to carry out any of the planned subgroup analyses on the primary outcome measure. Concern about small trial effects caused us to carry out a sensitivity analysis. Removal of all chronic migraine trials at high risk of bias from study size left just the two PREEMT trials, which gave a more conservative reduction of 2.0 days per month (95% CI -2.8 to -1.1, N=1384).

INSERT FIGURE 2

For peer review only

Migraine severity score on a 10 cm visual analogue scale (VAS), improved by -3.3 cm (95% CI -4.2 to -2.4) more with active treatment (fig 3). Only four small trials reported meta-analyzable data for this outcome. For Chronic migraine the improvement was -2.7 cm (95% CI -3.3 to -2.1, N=75), and for episodic migraine it was -4.9 cm (95% CI -6.6 to -3.2, N=34).

INSERT FIGURE 3

A reduction in the number headache days per month of 1.9 days (95% CI -2.7 to -1.0, 2 trials, N = 1384) in favor of BTX type-A treatment was also seen. However data for number of migraine attacks from six trials of both chronic migraine and episodic migraine participants (N = 2004) showed no significant between group difference (P = 0.30). Duration of migraine in hours was fully reported by only one trial showing a greater reduction of -5.1 hours (95% CI -6.2 to -4.0) for 102 chronic and episodic migraine participants. A further four trials with 420 participants reported no significant difference between groups for this outcome. Global assessment measures and quality of life measures were poorly reported and it was not possible to carry out statistical analysis of these outcome measures.

Effectiveness of BTX versus oral prophylactic agents

Three trials with 178 participants compared Botox® injections with oral prophylactic agents using double dummy techniques. Two trials compared 100 U fixed dose plus optional dose of up to 100 U of Botox® with topiramate maximum dose 200 mg/day.[42 43] The third trial compared treatment with up to 100 U Botox® with sodium valproate 250 mg twice daily.[41] Fourteen of the 178 participants had episodic migraine, all other participants had chronic migraine. Where meta-analysis was possible we pooled data from these three trials as there were insufficient data to allow us to explore comparisons with individual drug types or effects on chronic migraine and episodic migraine populations.

For peer review only

The primary outcome, number of migraine days per month was recorded in only one of the active comparison trials. The trialists reported that there was no statistically significant difference between treatment with BTX type-A and topiramate for this outcome.[43]

The number of headache days per month was recorded in two trials. No difference in number of headache days per month between treatment with BTX type-A and sodium valproate was reported (P=0.55).[35] No data were reported but it was stated that there was also no statistically significant difference between BTX type-A and topiramate treated groups.[42] A 5-point scale was used to compare the effect of BTX type-A with alternative agents in two trials, Blumenfeld et al reported no significant difference and Mathew et al reported within group analysis only.[41 43] Number of migraine attacks and duration of migraine were not reported by any trial. No difference between BTX type-A and topiramate was stated for use of rescue medications.[43]

Of all the secondary outcome measures, data for meta-analysis were available only for the Migraine disability assessment (MIDAS) scores. Results of this showed no significant difference in change scores between the established drug treatments and injection with Botox® (P = 0.80, 2 trials, N = 101).

Safety

BTX type-A had an RR of treatment related adverse events of twice that seen for placebo (2.2, 95% CI 1.7 to 2.8, 6 trials, N=2839) (fig 4). All of these events were transient and non-serious, the most common being blepharoptosis, muscle weakness, injection site pain and neck pain.

INSERT FIGURE 4

Compared with oral treatments, BTX type-A showed a reduced RR of treatment related adverse events of 0.76 (95% CI 0.59 to 0.98, 2 trials, N=73). There was also difference in

For peer review only

favor of BTX type-A in the RR of withdrawing due to adverse events of 0.28 (95% CI 0.10 to 0.79; I2 = 0%) which is a RR reduction of 72%.

A low withdrawal rate of 3% for BTX type-A was generated using data from all those trials treating with more than one injection cycle irrespective of the type of comparison arm.

Quality of the Evidence

The quality of the evidence assessed using GRADE methods was varied but mostly low and very low; the primary outcome measure was low and very low quality evidence for the placebo and active control comparisons respectively. Small trial size, high risk of bias and unexplained heterogeneity were common reasons for downgrading the quality of the evidence. All judgements and reasons for gradings are given in Supplemental files 4 and 5.

Discussion

Evidence was identified to support the use of injections of BTX type-A into the head and neck muscles, to reduce the number of migraine days experienced per month. Mean frequency of migraine days was significantly reduced by 3 days per month more by BTX type-A treatment than by placebo, but this result was revised to 2 days per month as a result of sensitivity analyses. All patients included in this analysis had chronic migraine and so had a high baseline frequency with an average of 20 days per month quoted by the two largest trials in the analysis.[6 7] For patients with chronic migraine, likely to be refractory to first and second line treatment, a 2-3 day improvement may well represent a meaningful difference. BTX type-A groups also fared better than placebo in the frequency of headache days by 2 days per month. Severity of migraine measured on a visual analogue scale was improved by 3 cm for chronic migraine and 5 cm for episodic migraine on a 10 cm scale. Though these results were from few small trials and the estimate is considered to be low quality evidence, the differences in severity scores were in excess of the minimal clinically important

For peer review only

difference of 1.2 cm determined by Kelly *et al.*[45] and indicate that the treatment may be reducing the impact of each migraine attack. In contrast to this no significant difference from placebo was observed for frequency of migraine attacks. Patient and clinician reported global assessment scales and quality of life scales were underused and when they were incorporated into trials they were poorly reported, so no aggregation of data of this type comparing investigative treatment with placebo was possible in this review.

It was not possible to carry out any analysis on headache diary outcomes or severity measures for head-to-head comparisons between BTX type-A and other established agents due to lack of available data. MIDAS scores for 101 patients from two small trials, one comparing Botox® with topiramate and one with sodium valproate were available and these showed no significant between group difference (P=0.8).

Trials included in this review commonly state that BTX's have good safety profiles and the evidence from the 23 trials included in this review which reported adverse events in some form support those assertions. Although an increased risk of experiencing treatment related adverse events was found for the BTX type-A treated group compared with placebo, the event types were non-serious and transient.

A relative risk reduction (RRR) of 24% in treatment related adverse events in favor of BTX type-A was found when comparing with topiramate and sodium valproate in two trials. These two trials found an RRR in favor of BTX type-A of 72% for withdrawal rate due to adverse events. Percentage withdrawals due to adverse events for all of those trials included in this review which used more than one round of BTX type-A injections, irrespective of the comparison arm type, was 3%. The data sets for the direct comparisons with other prophylactic agents were small, but the relationship is supported by the indirect comparison of this percentage with published rates of 20% for topiramate and 12% for sodium

For peer review only

valproate.[46 47] This result suggests that patients tolerate this treatment better than the oral alternatives.

Reporting was generally poor, with only six of 28 trials reporting data on our primary outcome in a usable format, and an additional five providing data for frequency of migraine attacks. These two outcomes are recommended as primary outcomes by the trial guidelines produced by the IHS and should be fully reported to allow individual trials to be placed in the context of the totality of the evidence.[48] A large proportion of the recorded data were missing from the published reports of our included trials. Failure to fully report data in trial publications led to problems throughout the meta-analysis and greater confidence in the conclusions would have been possible if all trials that recorded our outcomes of interest had fully reported them.

Prophylactic treatments for migraine aim to reduce the frequency, duration and/or the intensity of attacks. Frequency of migraine attacks was commonly used as the primary outcome particularly in studies carried out before the publication of the PREEMT trials. Use of this measure may mask an important improvement in symptoms seen in the form of shorter and less intense migraine attacks. Use of the more sensitive measures, number of days or hours spent with migraine per month coupled with a measure of intensity, may enable detection of such changes and could be particularly relevant to episodic migraine patients for whom attacks may be shorter at baseline. Another problem with focusing on this outcome measure was the failure generally to define what was meant by a migraine attack, and therefore, the likelihood of variation in the definitions used across the trials.

Neither efficacy nor safety data were available for long term treatment with BTX. The longest treatment period in any of the studies included in this review was three treatments with 12 weeks between treatments, so we cannot know the implications of treating patients with BTX over a period longer than 9 months.

For peer review only

Most trials did not report whether or not they had included patients with medication overuse symptoms and those that did stated they had largely excluded medication overuse patients. Pooled data for the two PREEMPT trials for the chronic migraine plus medication overuse subgroup (N=906) showed that the difference between groups for both migraine and headache day frequencies was 2 days (P<.001) in favor of treatment with BTX.[49] The medication overuse subgroup result falls within the confidence intervals of the pooled estimate generated by this review for the same outcome measure in combined populations with and without medication overuse headache. It would appear from these data that the inclusion of patients with medication overuse does not change the effectiveness of BTX for prophylactic treatment of migraine.

Conclusions

We have data which suggest that BTX effectively reduces the duration and severity of migraines in sufferers. There are however question marks over the quality of the evidence. Efficacy measures were commonly reported as showing non-inferiority of BTX to topiramate and sodium valproate and the withdrawal rate from BTX is much lower than that for first line prophylactic treatments for migraine. So should we be using more BTX?

It is currently recommended by NICE guidance that medication overuse headache should be addressed before treatment with BTX but trial data suggests it is efficacious in chronic migraine patients with untreated medication overuse headache. So although treatment of medication overuse headache is good practice, perhaps it should not be a requirement before prescription of BTX. NICE recommends the use of BTX to treat chronic migraine that has not responded to at least three prior pharmacological prophylaxis therapies. The confidence in the effectiveness of these drugs is arguably no greater than that for BTX and patients seem better able to tolerate BTX.[4 5 46 47] If, as is suggested by trial data, BTX has the equivalent

For peer review only

efficacy to other agents but lower withdrawal rates, then if it were not for the higher cost, BTX would likely be recommended as an earlier preventative treatment for chronic migraine. The difference between chronic and episodic migraine diagnoses is arbitrary and so there is no pathophysiological reason that treatment with BTX would be efficacious in people with 15 days headache per month and inefficacious in people with 14 days of headache per month in a stepwise fashion. The treatment may well be useful for episodic migraine, particularly in high frequency episodic migraine, but data is lacking.

Acknowledgements

We would like to thank Joanne Abbott, Information Specialist at the Cochrane Pain, Palliative and Supportive Care Group, who ran searches in MEDLINE, Embase and CENTRAL. We would also like to thank Ana Hughes, Cancer Clinical Trials Unit, University of Birmingham for her translation of Blumenkron 2006.

Author contributions

CEC, AS and JE conceived the review. CH ran searches not covered by the review group's information specialist. CH, CLT, CR and AS screened the search results. CH, CLT, CR, WS, CEC and AS assessed the quality of studies and extracted data. CH contacted trial authors, managed data and entered it into RevMan, and carried out data analysis. NI provided statistical advice. CH, CLT, CR, JE, NI, CEC and AS were involved in interpretation of the results. All authors read and edited final version of the review.

Funding

The authors received no financial support for the research, authorship, or publication of this article.

For peer review only

AJS is funded by an NIHR Clinician Scientist Fellowship (NIHR-CS-011-028), by the Medical Research Council, UK (MR/K015184/1)

Competing interests

JE received funding from Allergan in 2017 to attend a Master Class in Botulinum toxin. For all remaining authors none declared.

References

1. Collaborators GDaIIaP. Global, regional, and national incidence, prevalence, and years lived with disability for 310 diseases and injuries, 1990-2015: a systematic analysis for the Global Burden of Disease Study 2015. Lancet (London, England) 2016;**388**(10053):1545-602.
2. Linde M, Gustavsson A, Stovner LJ, et al. The cost of headache disorders in Europe: the Eurolight project. European Journal of Neurology 2012;**19**:703-11.
3. Linde K, Rossnagel K. Propranolol for migraine prophylaxis. Cochrane Database of Systematic Reviews 2004(2).
4. Linde M, Mulleners WM, Chronicle EP, et al. Gabapentin or pregabalin for the prophylaxis of episodic migraine in adults. Cochrane Database of Systematic Reviews 2013(6).
5. Linde M, Mulleners WM, Chronicle EP, et al. Antiepileptics other than gabapentin, pregabalin, topiramate, and valproate for the prophylaxis of episodic migraine in adults. Cochrane Database of Systematic Reviews 2013(6).
6. Aurora SK, Dodick DW, Turkel CC, et al. OnabotulinumtoxinA for treatment of chronic migraine: results from the double-blind, randomized, placebo-controlled phase of the PREEMPT 1 trial. Cephalalgia : an international journal of headache 2010;**30**(7):793-803.
7. Diener HC, Dodick DW, Aurora SK, et al. OnabotulinumtoxinA for treatment of chronic migraine: Results from the double-blind, randomized, placebo-controlled phase of the PREEMPT 2 trial. Cephalalgia 2010;**30**(7):804-14.
8. National Institute for Health Care Excellence. Botulinum toxin type A for the prevention of headaches in adults with chronic migraine. NICE technology appraisal guidance [TA260]. Published date: June 2012. Available from http://wwwniceorguk/guidance/ta260.
9. National Institute for Health Care Excellence. Headaches: Diagnosis and management of headaches in young people and adults. NICE guidelines [CG150]. Published date: September 2012. Available from http://wwwniceorguk/guidance/cg150.
10. British Medical Association, Society RP. British National Formulary (BNF). Available from wwwbnforg.
11. Headache Classification Committee of the International Headache S. The international classification of headache disorders, 3rd edition. Cephalalgia 2013;**33**(9):629-808.

For peer review only

12. Headache Classification Committee of the International Headache Society. The International Classification of Headache Disorders, 2nd edition, 1st revision. Cephalalgia 2004;**24**(Suppl 1):1-160.
13. Herd CP, Tomlinson CL, Rick C, et al. Botulinum toxins for the prevention of migraine in adults. Cochrane Database Syst Rev 2018;**6**:CD011616.
14. Headache Classification Committee of the International Headache Society. Classification and diagnostic criteria for headache disorders, cranial neuralgias and facial pain. Cephalalgia 1988;**8**(Suppl. 7):1-96.
15. Review Manager (RevMan) [program]. Version 5.3 version. Copenhagen: The Cochrane Collaboration, 2014.
16. DerSimonian R, Laird N. Meta-analysis in clinical trials. Controlled clinical trials 1986;**7**(3):177-88.
17. Dechartres A, Trinquart L, Boutron I, et al. Influence of trial sample size on treatment effect estimates: meta-epidemiological study. BMJ 2013;**346**:f2304-f04.
18. Nüesch E, Trelle S, Reichenbach S, et al. Small study effects in meta-analyses of osteoarthritis trials: Meta-epidemiological study. BMJ 2010;**341**:c3515-c15.
19. Guyatt GH, Oxman AD, Vist GE, et al. GRADE: an emerging consensus on rating quality of evidence and strength of recommendations. BMJ 2008;**336**(7650):924-26.
20. Anand KS, Prasad A, Singh MM, et al. Botulinum toxin type A in prophylactic treatment of migraine. American journal of therapeutics 2006;**13**(3):183-7.
21. Aurora SK, Gawel M, Brandes JL, et al. Botulinum toxin type a prophylactic treatment of episodic migraine: a randomized, double-blind, placebo-controlled exploratory study. Headache 2007;**47**(4):486-99.
22. Barrientos N, Chana P. Botulinum toxin type A in prophylactic treatment of migraine headaches: A preliminary study. Journal of headache and pain 2003;**4**(3):146-51.
23. Blumenkron D, Rivera C, Cuevas C. Efficacy of botulinum toxin type A in patients with migraine. [Spanish] Eficacia del tratamiento con toxina botulinica tipo A en pacientes con migrana. Medicina Interna de Mexico 2006;**22**(1):25-31.
24. Cady R, Schreiber C. Botulinum toxin type A as migraine preventive treatment in patients previously failing oral prophylactic treatment due to compliance issues. Headache 2008;**48**(6):900-13.
25. Chankrachang S, Arayawichanont A, Poungvarin N, et al. Prophylactic botulinum type A toxin complex (Dysport®) for migraine without aura. Headache 2011;**51**(1):52-63.
26. Elkind AH, O'Carroll P, Blumenfeld A, et al. A series of three sequential, randomized, controlled studies of repeated treatments with botulinum toxin type A for migraine prophylaxis. The journal of pain : official journal of the American Pain Society 2006;**7**(10):688-96.
27. Freitag FG, Diamond S, Diamond M, et al. Botulinum Toxin Type A in the treatment of chronic migraine without medication overuse. Headache 2008;**48**(2):201-9.
28. Hollanda L, Monteiro L, Melo AS. Botulinum toxin type A for cephalic cutaneous allodynia in chronic migraine: A randomized, double-blinded, placebo-controlled trial. Neurology international 2014;**6**(4):70-3.
29. Hou M, Xie JF, Kong XP, et al. Acupoint injection of onabotulinumtoxin a for migraines. Toxins 2015;**7**(11):4442-54.
30. Jost WH. Low-dosed botulinum toxin a in the prophylactic management of unilateral migraine: A randomized double-blind placebo-controlled crossover study. Open pain journal 2011;**4**(1):4-7.
31. Lauretti GR, Rosa CP, Kitayama A, et al. Comparison of Botox® or Prosigne® and Facial Nerve Blockade as Adjuvant in Chronic Migraine. Journal of Biomedical Science and Engineering 2014;**7**:446-52.

For peer review only

32. Petri S, Tölle T, Straube A, et al. Botulinum toxin as preventive treatment for migraine: a randomized double-blind study. European neurology 2009;**62**(4):204-11.
33. Relja M, Poole AC, Schoenen J, et al. A multicentre, double-blind, randomized, placebo-controlled, parallel group study of multiple treatments of botulinum toxin type A (BoNTA) for the prophylaxis of episodic migraine headaches. Cephalalgia : an international journal of headache 2007;**27**(6):492-503.
34. Saper JR, Mathew NT, Loder EW, et al. A double-blind, randomized, placebo-controlled comparison of botulinum toxin type a injection sites and doses in the prevention of episodic migraine. Pain medicine (Malden, Mass) 2007;**8**(6):478-85.
35. Silberstein S, Mathew N, Saper J, et al. Botulinum toxin type A as a migraine preventive treatment. Headache 2000;**40**(6):445-50.
36. Cady R, Turner I, Dexter K, et al. An exploratory study of salivary calcitonin gene-related peptide levels relative to acute interventions and preventative treatment with onabotulinumtoxinA in chronic migraine. Headache 2014;**54**(2):269-77.
37. Vo AH, Satori R, Jabbari B, et al. Botulinum toxin type-a in the prevention of migraine: a double-blind controlled trial. Aviation, space, and environmental medicine 2007;**78**(5 Suppl):B113-8.
38. Jabbari B. Investigation of Efficacy and Safety of Botulinum Toxin A (Botox-Allergan Inc) in Migraine Headaches. clinicaltrials.gov, 2008.
39. Mazza MR, Ferrigno G, Vescio B, et al. Subcutaneous botulinum toxin type a treatment for prophylaxis of headaches in chronic migraine: A new therapeutic strategy. Cephalalgia 2016;**36**:35.
40. Allergan. Use of a Treatment Benefit Questionnaire in Patients With Chronic Migraine Treated With OnabotulinumtoxinA (BOTOX®). clinicaltrials.gov, 2013.
41. Blumenfeld AM, Schim JD, Chippendale TJ. Botulinum toxin type A and divalproex sodium for prophylactic treatment of episodic or chronic migraine. Headache 2008;**48**(2):210-20.
42. Cady RK, Schreiber CP, Porter JA, et al. A multi-center double-blind pilot comparison of onabotulinumtoxinA and topiramate for the prophylactic treatment of chronic migraine. Headache 2011;**51**(1):21-32.
43. Mathew NT, Jaffri SF. A double-blind comparison of onabotulinumtoxina (BOTOX) and topiramate (TOPAMAX) for the prophylactic treatment of chronic migraine: a pilot study. Headache 2009;**49**(10):1466-78.
44. Millán-Guerrero RO, Isais-Millán S, Barreto-Vizcaíno S, et al. Subcutaneous histamine versus botulinum toxin type A in migraine prophylaxis: a randomized, double-blind study. European journal of neurology 2009;**16**(1):88-94.
45. Kelly AM. The minimum clinically significant difference in visual analogue scale pain score does not differ with severity of pain. Emerg Med J 2001;**18**:205–07.
46. Linde M, Mulleners WM, Chronicle EP, et al. Valproate (valproic acid or sodium valproate or a combination of the two) for the prophylaxis of episodic migraine in adults. Cochrane Database of Systematic Reviews 2013(6).
47. Linde M, Mulleners WM, Chronicle EP, et al. Topiramate for the prophylaxis of episodic migraine in adults. Cochrane Database of Systematic Reviews 2013(6).
48. Tfelt-Hansen P, Pascual J, Ramadan N, et al. Guidelines for controlled trials of drugs in migraine: third edition. A guide for investigators. Cephalalgia 2012;**32**(1):6-38.
49. Silberstein SD, Blumenfeld AM, Cady RK, et al. OnabotulinumtoxinA for treatment of chronic migraine: PREEMPT 24-week pooled subgroup analysis of patients who had acute headache medication overuse at baseline. Journal of the neurological sciences 2013;**331**(1-2):48-56.

For peer review only

Figure legends

Figure 1: Risk of bias graph: review authors' judgements about each risk of bias item presented as percentages across all included studies.

Figure 2: Comparison of BTX type-A versus placebo in relation to number of migraine days per month.

Figure 3: Comparison of BTX type-A versus placebo in relation to severity of migraine measured on a 10 cm visual analogue scale.

Figure 4: Comparison of BTX type-A versus placebo in relation to treatment related adverse events.

For peer review only

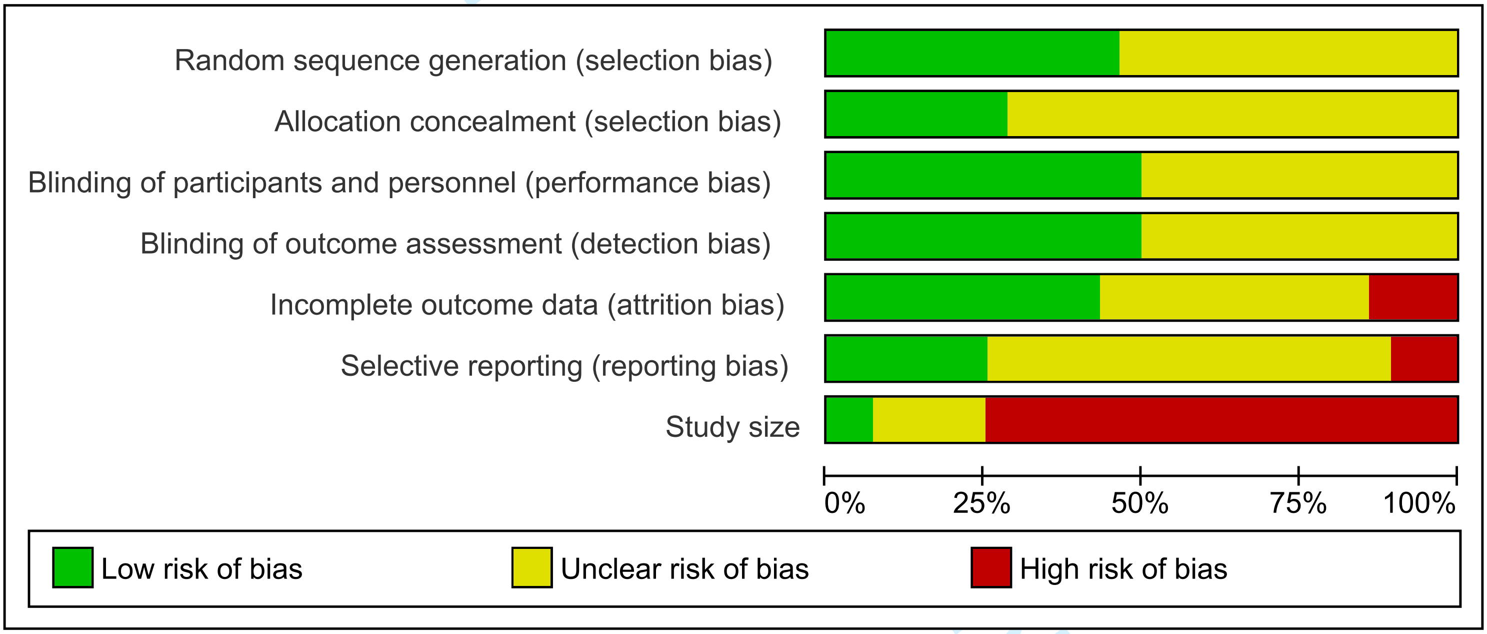
Random sequence generation (selection bias)
Allocation concealment (selection bias)
Blinding of participants and personnel (performance bias)
Blinding of outcome assessment (detection bias)
Incomplete outcome data (attrition bias)
Selective reporting (reporting bias)
Study size
0%
25%
50%
75%
100%
Low risk of bias
Unclear risk of bias
High risk of bias

Study or Subgroup	Botulinum toxin Mean [Days]	SD [Days]	Total	Placebo Mean [Days]	SD [Days]	Total	Weight	Mean Difference IV, Random, 95% CI [Days]
**1.1.1 Chronic migraine**								
Aurora 2010 (PREEMPT 1)	-7.6	6.5	341	-6.1	6.8	338	24.3%	-1.50 [-2.50, -0.50]
Cady 2014	-10.1	5.9	9	-5.2	5.1	10	7.6%	-4.90 [-9.88, 0.08]
Diener 2010 (PREEMPT 2)	-8.7	6.6	347	-6.3	6.7	358	24.4%	-2.40 [-3.38, -1.42]
Mazza 2016	9.4	8.6	63	15.1	1.8	31	17.9%	-5.70 [-7.92, -3.48]
**Subtotal (95% CI)**			**760**			**737**	**74.1%**	**-3.07 [-4.73, -1.41]**
Heterogeneity: Tau² = 1.84; Chi² = 12.56, df = 3 (P = 0.006); I² = 76%								
Test for overall effect: Z = 3.62 (P = 0.0003)								
**1.1.2 Episodic migraine**								
Elkind I 2006	-1.4	2.5	312	-1.2	2.6	106	25.9%	-0.20 [-0.77, 0.37]
**Subtotal (95% CI)**			**312**			**106**	**25.9%**	**-0.20 [-0.77, 0.37]**
Heterogeneity: Not applicable								
Test for overall effect: Z = 0.69 (P = 0.49)								
**Total (95% CI)**			**1072**			**843**	**100.0%**	**-2.39 [-4.02, -0.76]**
Heterogeneity: Tau² = 2.59; Chi² = 35.80, df = 4 (P < 0.00001); I² = 89%								
Test for overall effect: Z = 2.88 (P = 0.004)								
Test for subgroup differences: Chi² = 10.26, df = 1 (P = 0.001), I² = 90.3%								

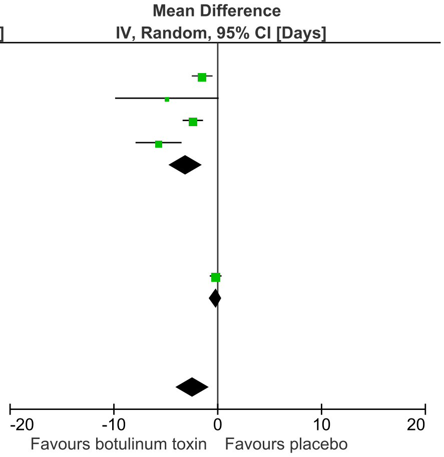

For peer review only

Study or Subgroup	Botulinum toxin Mean	SD	Total	Placebo Mean	SD	Total	Weight	Mean Difference IV, Random, 95% CI
**1.4.1 Chronic migraine**								
Hollanda 2014	3.45	2.39	20	6.17	2.5	18	18.3%	-2.72 [-4.28, -1.16]
Lauretti 2014	6.5	1.3	28	9.2	0.7	9	36.6%	-2.70 [-3.36, -2.04]
**Subtotal (95% CI)**			**48**			**27**	**54.9%**	**-2.70 [-3.31, -2.09]**
Heterogeneity: Tau² = 0.00; Chi² = 0.00, df = 1 (P = 0.98); I² = 0%								
Test for overall effect: Z = 8.67 (P < 0.00001)								
**1.4.2 Episodic migraine**								
Anand 2006	2.4	3.2	16	7.3	1.1	16	17.0%	-4.90 [-6.56, -3.24]
**Subtotal (95% CI)**			**16**			**16**	**17.0%**	**-4.90 [-6.56, -3.24]**
Heterogeneity: Not applicable								
Test for overall effect: Z = 5.79 (P < 0.00001)								
**1.4.3 Mixed**								
Hou 2015	2.8	1.2	83	6.3	2.2	19	28.1%	-3.50 [-4.52, -2.48]
**Subtotal (95% CI)**			**83**			**19**	**28.1%**	**-3.50 [-4.52, -2.48]**
Heterogeneity: Not applicable								
Test for overall effect: Z = 6.71 (P < 0.00001)								
**Total (95% CI)**			**147**			**62**	**100.0%**	**-3.30 [-4.16, -2.45]**
Heterogeneity: Tau² = 0.40; Chi² = 6.74, df = 3 (P = 0.08); I² = 55%								
Test for overall effect: Z = 7.58 (P < 0.00001)								
Test for subgroup differences: Chi² = 6.74, df = 2 (P = 0.03), I² = 70.3%								

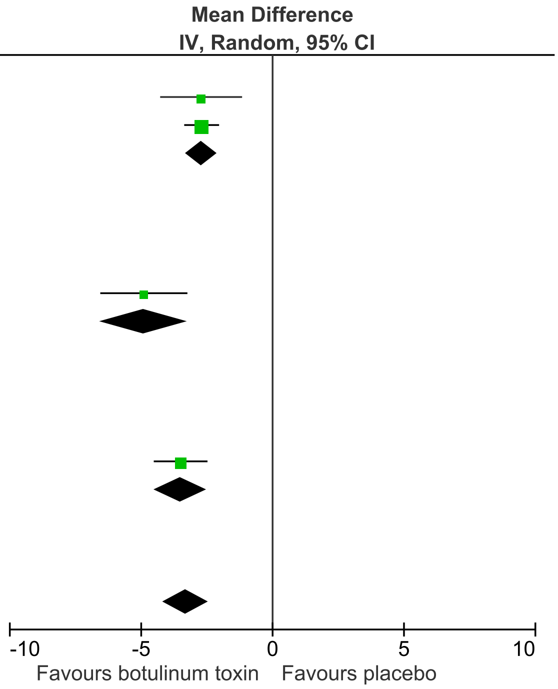

Study or Subgroup	Botulinum toxin Events	Botulinum toxin Total	Placebo Events	Placebo Total	Weight	Risk Ratio M-H, Random, 95% CI
**1.11.1 Chronic migraine**						
Aurora 2010 (PREEMPT 1)	86	340	39	334	18.6%	2.17 [1.53, 3.06]
Diener 2010 (PREEMPT 2)	116	347	49	358	20.8%	2.44 [1.81, 3.30]
**Subtotal (95% CI)**		**687**		**692**	**39.3%**	**2.32 [1.85, 2.91]**
Total events	202		88			
Heterogeneity: Tau² = 0.00; Chi² = 0.26, df = 1 (P = 0.61); I² = 0%						
Test for overall effect: Z = 7.28 (P < 0.00001)						
**1.11.2 Episodic migraine**						
Aurora 2007	113	187	39	182	20.7%	2.82 [2.09, 3.81]
Elkind I 2006	61	312	7	106	7.3%	2.96 [1.40, 6.27]
Relja 2007	243	377	37	118	21.9%	2.06 [1.56, 2.71]
Saper 2007	45	187	11	45	10.8%	0.98 [0.55, 1.75]
**Subtotal (95% CI)**		**1063**		**451**	**60.7%**	**2.06 [1.37, 3.08]**
Total events	462		94			
Heterogeneity: Tau² = 0.11; Chi² = 11.01, df = 3 (P = 0.01); I² = 73%						
Test for overall effect: Z = 3.50 (P = 0.0005)						
**Total (95% CI)**		**1750**		**1143**	**100.0%**	**2.18 [1.73, 2.75]**
Total events	664		182			
Heterogeneity: Tau² = 0.04; Chi² = 11.41, df = 5 (P = 0.04); I² = 56%						
Test for overall effect: Z = 6.59 (P < 0.00001)						
Test for subgroup differences: Chi² = 0.26, df = 1 (P = 0.61), I² = 0%						

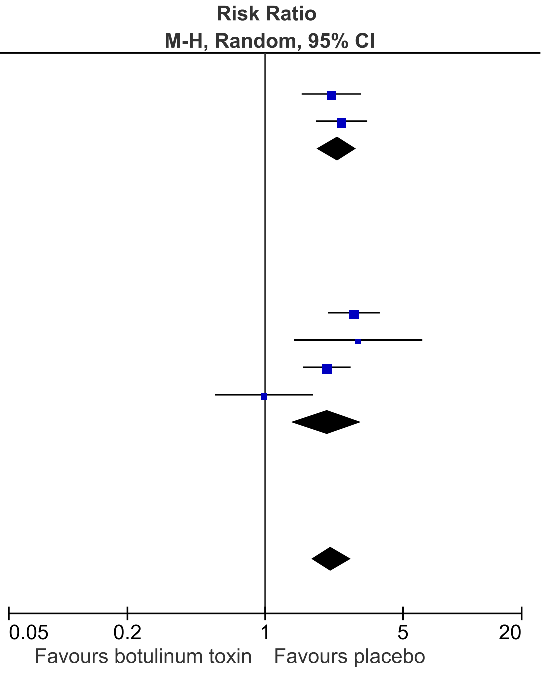

MEDLINE (via OVID)

#1 Exp headache disorders/

#2 headache/

#3 (headache* or migrain* or cephalgi* or cephalalgi* or hemicrani*).mp.

#4 or/1-3

#5 exp botulinum toxins/

#6 (botulin* adj toxin*).tw

#7 (botulinum* or oculinu* or boto* or onabotulinum*).tw.

#8 exp botulinum toxin type A/

#9 Exp clostridium botulinum/

#10 clostridium botulin*.tw.

#11 or/5-10

12 randomized controlled trial.pt.

13 controlled clinical trial.pt.

14 randomized.ab.

15 placebo.ab.

16 drug therapy.fs.

17 randomly.ab.

18 trial.ab.

19 groups.ab.

20 12 or 13 or 14 or 15 or 16 or 17 or 18 or 19

21 exp animals/ not humans.sh.

22 20 not 21

23 4 and 11 and 22

For peer review only

PRISMA 2009 Flow Diagram

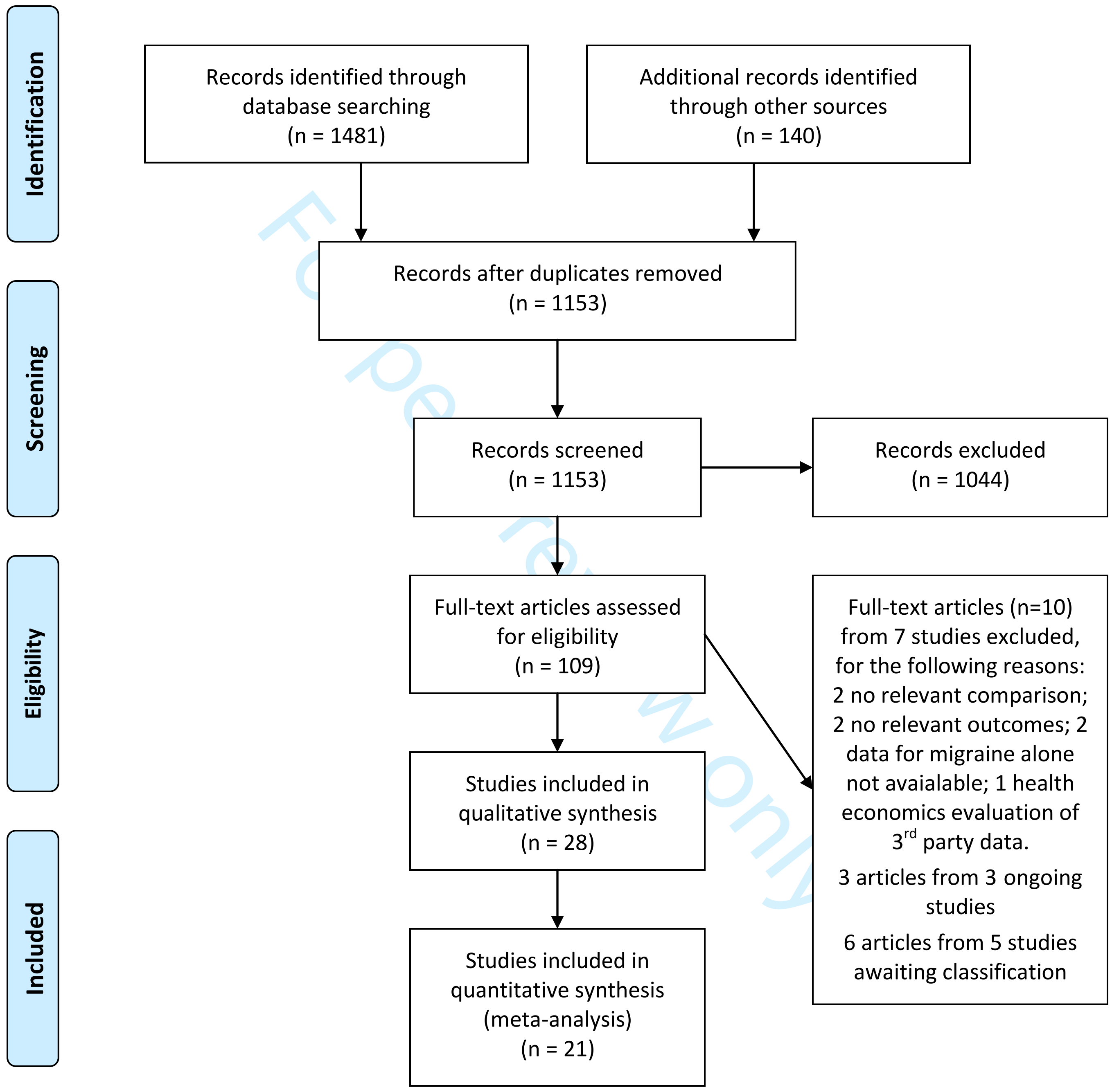

From: Moher D, Liberati A, Tetzlaff J, Altman DG, The PRISMA Group (2009). *P*referred *R*eporting *I*tems for *S*ystematic Reviews and *M*eta-*A*nalyses: The PRISMA Statement. PLoS Med 6(7): e1000097. doi:10.1371/journal.pmed1000097

For more information, visit www.prisma-statement.org.

For peer review only - http://bmjopen.bmj.com/site/about/guidelines.xhtml

Table 1: Characteristics of botulinum toxin vs. placebo studies

Trial (Reference)	Total N randomised	Comparison	No of injection sites (Fixed or FTP)	Location of injection sites	No. treatment cycles	Mean Age (SD)	EM/CM	Ratio of MOH/No MOH
Allergan 2015 (40)	52	155 U Botox® vs Placebo	nr Fixed	Head and neck; no. sites/units per region nr	2	42.8(12.2)	All CM	nr
Anand 2006 (20)	32	50 U Botox® vs Placebo	10 Fixed	3 pericranial muscle regions; no. sites/units per region nr	1	nr	All EM	0/32
Aurora 2010 (PREEMPT 1) (6)	679	Botox® 155-195 U vs Placebo	31 Fixed + 8 OFTP	Seven specific head/neck muscle areas; no. sites/units per region nr	2	41.6	All CM	462/217
Aurora 2007 (21)	369	Pooled Botox® vs Placebo from: Placebo responders(PR) Botox® vs PR Placebo vs Placebo non-responders (PNR) Botox vs PNR	23-58 FTP	Frontal/glabellar, 25-40 U; occipitalis, fixed, 20 U; temporalis, 20-50 U; masseter optional, 0-50 U; Trapezius, 20-60 U; Semispinalis, 10-20 U; Splenius capitis, 10-20 U; no. sites per region nr	3	45	All EM	0/369
Barrientos 2003 (22)	30	Botox® 50 U vs placebo	15 Fixed	Temporalis, 10 U, 2 sites; frontalis, 10 U, 4 sites; glabellar, 8 U, 4 sites; procerus, 2 U, 1 site; trapezius, 10 U, 2 sites; splenius capitis, 10 U, 2 sites.	1	41.1	All EM	0/30
Blumenkron 2006 (23)	30	Botox® 100 U vs placebo	25 Fixed	Front points 10 U, 4 sites, procerus muscle 10 U, 1 site, corrugator muscles 10 U, 2 sites, temporalis muscles 30 U, 6 sites, trapezius muscle 50 U, 10 sites	1	nr	nr	nr
Cady 2008 (24)	61	Botox® 139 U vs placebo	17 Fixed	Corrugator, 12 U, 2 sites; splenius capitis, 20 U, 2 sites; trapezius, 40 U, 4 sites; temporalis, 40 U, 4 sites; procerus, 3 U, 1 site; frontalis, 24 U, 4 sites	1	42.1(11.5)	nr	nr
Cady 2014 (36)	20	Botox® 155 U vs placebo (cross-over design)	31 Fixed + nr OFTP	PREEMPT paradigm + optional follow the pain in occipitalis, temporalis, and trapezius; no. sites/units per region for OFTP nr	1	48.5(12.9)	All CM	nr
Chankrachang 2011 (25)	128	DYSPORT® 120 U vs DYSPORT® 240 U vs placebo	6 Fixed	Frontal, 2 sites; temporal, 2 sites; occipital, 2 sites; units per region nr	1	38.6(9.9)	All CM	nr
Diener 2010 (PREEMPT 2) (7)	705	Botox® 155-195 U vs Placebo	31 Fixed + 8 OFTP	Seven specific head/neck muscle areas; no. sites/units per region nr	2	40.9	All CM	444/261
Elkind I 2006 (26)	418	50 U Botox® vs 25 U Botox® vs 7.5 U Botox® vs Placebo	11 Fixed	Frontal, 4 sites; temporal, 2 sites; glabellar, 5 sites; units per region nr	1	44.1	409 EM/9 CM	nr
Freitag 2008 (27)	41	Botox® 100 U vs placebo	22 Fixed	Glabella, 20 U, 4 sites; temporal 20 U, 4 sites; frontal 10 U, 4 sites; suboccipital 30 U, 6 sites; trapezius 20 U, 4 sites	1	42.3	All CM	0/41
Hollanda 2014 (28)	38	PROSIGNE® 96 U (max) vs Placebo	12-24 FTP	Frontal 12-24 U, 4-8 sites; temporal 16-32 U, 4-8 sites; occipital 20-40 U, 4-8 sites	1	45.3(13.4)	All CM	0/38
Hou 2015 (29)	102	HENGLI® 25 U fixed vs HENGLI® 25 U acupoint vs placebo	10 Fixed	Fixed & placebo: frontal & occipital belly of occipitofrontalis, corrugator supercilii, temporalis & superior part of trapeziue. Acupoint: Yintang (EX-HN3), Taiyang (EX-HN5), Baihui (GV20), Shuaigu (GB8), Fengchi (GB20), Tianzhu (BL10); no. sites/units per region nr	1	40.7(9.0)	66EM/36CM	nr

For peer review only - http://bmjopen.bmj.com/site/about/guidelines.xhtml

Jabbari 2014 (38)	25	Botox 200-300 U vs placebo	22-24 Fixed	Frontalis, 40 U, 10 sites; temporalis, 60 U, 4 sites; occipitalis 10 U, 2 sites; cervical region: splenius cervicis, semispinalis capitis, trapezius muscles, 90-120 U, 6-10 sites	1	60	All CM	nr
Jost 2011 (30)	22	Botox® 10 U vs placebo (cross-over design)	2 FTP	Corrugator muscle and the occipitalis muscle of the side affected; no. sites/units per region nr	1	45.2(11.1)	nr	nr
Lauretti 2009 (31)	40	25 U Botox® vs 25 U Prosigne® vs 33.3 U Prosigne® vs saline	10 Fixed	Frontal region, 8 sites; temporal region, 2 sites; units per region nr	1	45.7(14.3)	All CM	nr
Mazza 2016 (39)	94	Botulinum toxin type A (Brand not reported) up to 200 U vs Placebo	nr FTP	Subcutaneous injections into trigeminal or occipital areas depending upon area of maximal pain; no. sites per region nr	2	nr	All CM	nr
Petri 2009 (32)	127	DYSPORT® 210 U vs DYSPORT® 80 U vs placebo	18 Fixed	Trapezius 45 U, splenius capitis 20 U, temporalis 20 U, frontalis 10 U, corrugator 10 U; no. sites per region nr	1	46.2(11.8)	All EM	0/127
Relja 2007 (33)	495	Botox® 225 U vs Botox ® 150 U vs Botox® 75 U vs Placebo	20 Fixed	Frontalis, 4 sites; corrugator, 2 sites; temporalis, 4 sites; splenius capitis, 2 sites; trapezius, 4 sites; semispinalis capitis, 2 sites; suboccipital region, 2 sites; units per region nr	3	43.2	All EM	0/495
Saper 2007 (34)	232	Botox® (25 U) all muscle sites vs Botox® (10 U) frontal vs Botox® (6 U) temporal vs Botox® (9 U) glabellar vs placebo all muscle sites	11 Fixed	Frontal, 4 sites; temporal, 2 sites; glabellar, 5 sites; units per region nr	1	43.6	All EM	0/232
Silberstein 2000 (35)	123	Botox® 75 U vs Botox® 25 U vs placebo	11 Fixed	Frontalis, 30 U; temporalis, 18 U; glabellar, 27 U; no. sites per region nr	1	44	All EM	0/123
Vo 2007 (37)	49	Botulinum toxin type A (Brand not reported) vs Placebo	22 Fixed	Corrugator 5 U, 2 sites; frontalis, 20 U, 4 sites; temporalis, 20 U (≥65 kg, 40 U), 4 sites; posterior neck, 60 U (≥65 kg, 90 U), 6 sites; occipitalis, 10 U, 2 sites; sternocleidomastoid, 20 U (≥65 kg, 40 U), 4 sites	1	42.4(8.3)	EM/CM ratio nr	nr

CM, chronic migraine; EM, episodic migraine; FTP, follow the pain; MOH, medication overuse headache; nr, not reported; OFTP, optional follow the pain.

For peer review only - http://bmjopen.bmj.com/site/about/guidelines.xhtml

Table 2: Characteristics of botulinum toxin vs. oral prophylactic agent studies

Trial (Reference)	Total N randomised	Comparison	No of injection sites (Fixed or FTP)	Location of injection sites	No. treatment cycles	Mean Age (SD)	EM/CM	Ratio of MOH/No MOH
Blumenfeld 2008 (41)	59	BOTOX ® ≤100 U vs DVPX;DEPAKOTE® 250 mg twice daily	nr FTP	Procerus, 2.5-5 U; Corrugators, 2.5-5 U; frontalis, 25 U; temporalis, 7.5-20 U; splenius capitis, 2.5-10 U; sternocleidomastoid, 7.5-15 U; trapezius, 2.5-5 U; occipitalis, 2.5-5 U; cervical paraspinalis, 7.5-15 U; semispinalis capitis, 5-10 U; masseter, 5-15 U	2	42.1(10.3)	45/14	0/59
Cady 2011 (42)	59	OnabotulinumtoxinA up to 200 U vs Topiramate 25 mg escalated to max 200 mg (ave 136 mg)	nr Fixed + nr OFTP	100 U fixed sites + up to 100 U in FTP	1	39.6	All CM	0/59
Mathew 2009 (43)	60	BOTOX® 200 U vs TOPAMAX® (topiramate) 100-200 mg/day	nr Fixed + nr OFTP	100 U fixed sites + up to 100 U in FTP	2	36.8(10.3)	All CM	nr

CM, chronic migraine; EM, episodic migraine; FTP, follow the pain; MOH, medication overuse headache; nr, not reported; OFTP, optional follow the pain.

For peer review only

BTX-A compared to placebo for the prevention of migraine in adults

Outcomes	Result with BTX-A (95% CI)	Number of participants (studies)	Quality of the evidence (GRADE)
Number of migraine days per month - Chronic migraine	MD 3.1 days lower (4.7 lower to 1.4 lower)	1497 (4 RCTs)	⊕⊕⊖⊖ LOW [a b]
Number of headache days per month - Chronic migraine	MD 1.9 days lower (2.7 lower to 1.0 lower)	1384 (2 RCTs)	⊕⊕⊕⊕ HIGH
Number of migraine attacks	MD 0.5 attacks lower (1.3 lower to 0.4 higher)	2004 (6 RCTs)	⊕⊕⊖⊖ LOW [c d]
Headache intensity measure - Chronic migraine (Visual Analogue Score 0-10)	MD 2.7 cm lower (3.3 lower to 2.1 lower)	75 (2 RCTs)	⊕⊖⊖⊖ VERY LOW [e f]
Headache intensity measure - Episodic migraine (Visual Analogue Score 0-10)	MD 4.9 cm lower (6.6 lower to 3.2 lower)	75 (1 RCT)	⊕⊖⊖⊖ VERY LOW [e f]
Headache Impact Test-6	MD 1.6 points higher (2.1 lower to 5.3 higher)	45 (1 RCT)	⊕⊖⊖⊖ VERY LOW [e f]
Total number of participants experiencing an adverse event	RR 1.28 (1.1 to 1.5)	3325 (13 RCTs)	⊕⊕⊕⊖ MODERATE [g]

Footnotes

CI: Confidence interval; RR: Risk ratio; MD: Mean difference. [a] Downgraded once due to inconsistency: Statistical heterogeneity observed despite similarities in populations and doses. [b] Downgraded once due to imprecision: Sensitivity analysis testing robustness of result suggested small studies may be over estimating treatment effect. [c] Downgraded once due to indirectness: Sensitivity of this outcome measure at risk of being too low to detect clinically

For peer review only

meaningful differences. [d] Downgraded once due to publication bias: Evidence found of trials that have never been published which record this outcome. [e] Downgraded once due to risk of bias: High or unclear risk of selective reporting bias and poor reporting of this outcome measure had a large effect on numbers analyzed. [f] Downgraded twice due to imprecision: Study size small, new trial evidence likely to change result. [g] Downgraded once due to imprecision: Study size small, new trial evidence likely to change result. GRADE Working Group grades of evidence- High quality: We are very confident that the true effect lies close to that of the estimate of the effect; Moderate quality: We are moderately confident in the effect estimate: The true effect is likely to be close to the estimate of the effect, but there is a possibility that it is substantially different; Low quality: Our confidence in the effect estimate is limited: The true effect may be substantially different from the estimate of the effect; Very low quality: We have very little confidence in the effect estimate: The true effect is likely to be substantially different from the estimate of effect

For peer review only

BTX-A compared to other established prophylactic agent for the prevention of migraine in adults

Outcomes	Result with BTX-A (95% CI)	Number of participants (studies)	Quality of the evidence (GRADE)
Number of migraine days per month - chronic migraine	One trial using topiramate in its comparison arm reported narratively on this outcome stating that there was no significant difference between groups.	43 (1 RCT)	⊕⊖⊖⊖ VERY LOW [a b c]
Number of headache days per month	MD 1 days lower (4.3 lower to 2.3 higher)	59 (1 RCT)	⊕⊖⊖⊖ VERY LOW [a b]
Headache intensity measure assessed with: 5-point scale, 5 being severe, 1 being mild - chronic migraine only	MD 0.4 points lower (0.79 lower to 0.01 lower)	46 (1 RCT)	⊕⊖⊖⊖ VERY LOW [a b]
Global impression of disease assessed with: Migraine impact and disability assessment scores	MD 4.3 points higher (28 lower to 37 higher)	101 (2 RCTs)	⊕⊖⊖⊖ VERY LOW [a b]
Total number of participants experiencing an adverse event	RR 0.8 (0.4 to 1.9)	114 (2 RCTs)	⊕⊖⊖⊖ VERY LOW [a b]

Footnotes

CI: Confidence interval; RR: Risk ratio; MD: Mean difference. [a] Downgraded once due to risk of bias: Unclear or high risk for selection, performance, detection and attrition bias. [b] Downgraded twice due to imprecision: Study sizes small, new trial evidence likely to change result. [c] Downgraded once due to imprecision: Narrative description only. GRADE

Working Group grades of evidence- High quality: We are very confident that the true effect lies close to that of the estimate of the effect; Moderate quality: We are moderately confident in the effect estimate: The true effect is likely to be close to the estimate of the effect, but there is a possibility that it is substantially different; Low quality: Our confidence in the effect estimate is limited: The true effect may be substantially different from the estimate of the effect; Very low quality: We have very little confidence in the effect estimate: The true effect is likely to be substantially different from the estimate of effect.

For peer review only

PRISMA 2009 Checklist

Section/topic	#	Checklist item	Reported on page #
**TITLE**			
Title	1	Identify the report as a systematic review, meta-analysis, or both.	1
**ABSTRACT**			
Structured summary	2	Provide a structured summary including, as applicable: background; objectives; data sources; study eligibility criteria, participants, and interventions; study appraisal and synthesis methods; results; limitations; conclusions and implications of key findings; systematic review registration number.	2-3
**INTRODUCTION**			
Rationale	3	Describe the rationale for the review in the context of what is already known.	4-5
Objectives	4	Provide an explicit statement of questions being addressed with reference to participants, interventions, comparisons, outcomes, and study design (PICOS).	5
**METHODS**			
Protocol and registration	5	Indicate if a review protocol exists, if and where it can be accessed (e.g., Web address), and, if available, provide registration information including registration number.	5
Eligibility criteria	6	Specify study characteristics (e.g., PICOS, length of follow-up) and report characteristics (e.g., years considered, language, publication status) used as criteria for eligibility, giving rationale.	6
Information sources	7	Describe all information sources (e.g., databases with dates of coverage, contact with study authors to identify additional studies) in the search and date last searched.	5-6
Search	8	Present full electronic search strategy for at least one database, including any limits used, such that it could be repeated.	Suppl.file 1
Study selection	9	State the process for selecting studies (i.e., screening, eligibility, included in systematic review, and, if applicable, included in the meta-analysis).	6
Data collection process	10	Describe method of data extraction from reports (e.g., piloted forms, independently, in duplicate) and any processes for obtaining and confirming data from investigators.	7
Data items	11	List and define all variables for which data were sought (e.g., PICOS, funding sources) and any assumptions and simplifications made.	7
Risk of bias in individual studies	12	Describe methods used for assessing risk of bias of individual studies (including specification of whether this was done at the study or outcome level), and how this information is to be used in any data synthesis.	6-7
Summary measures	13	State the principal summary measures (e.g., risk ratio, difference in means).	7
Synthesis of results	14	Describe the methods of handling data and combining results of studies, if done, including measures of consistency (e.g., I^2) for each meta-analysis.	7-8

For peer review only - http://bmjopen.bmj.com/site/about/guidelines.xhtml

PRISMA 2009 Checklist

Section/topic	#	Checklist item	Reported on page #
Risk of bias across studies	15	Specify any assessment of risk of bias that may affect the cumulative evidence (e.g., publication bias, selective reporting within studies).	8
Additional analyses	16	Describe methods of additional analyses (e.g., sensitivity or subgroup analyses, meta-regression), if done, indicating which were pre-specified.	8
**RESULTS**			
Study selection	17	Give numbers of studies screened, assessed for eligibility, and included in the review, with reasons for exclusions at each stage, ideally with a flow diagram.	9 + suppl.file2
Study characteristics	18	For each study, present characteristics for which data were extracted (e.g., study size, PICOS, follow-up period) and provide the citations.	9-10
Risk of bias within studies	19	Present data on risk of bias of each study and, if available, any outcome level assessment (see item 12).	9
Results of individual studies	20	For all outcomes considered (benefits or harms), present, for each study: (a) simple summary data for each intervention group (b) effect estimates and confidence intervals, ideally with a forest plot.	10-12
Synthesis of results	21	Present results of each meta-analysis done, including confidence intervals and measures of consistency.	10-12
Risk of bias across studies	22	Present results of any assessment of risk of bias across studies (see Item 15).	13+suppl. Files 4&5
Additional analysis	23	Give results of additional analyses, if done (e.g., sensitivity or subgroup analyses, meta-regression [see Item 16]).	10
**DISCUSSION**			
Summary of evidence	24	Summarize the main findings including the strength of evidence for each main outcome; consider their relevance to key groups (e.g., healthcare providers, users, and policy makers).	13
Limitations	25	Discuss limitations at study and outcome level (e.g., risk of bias), and at review-level (e.g., incomplete retrieval of identified research, reporting bias).	13-16
Conclusions	26	Provide a general interpretation of the results in the context of other evidence, and implications for future research.	16
**FUNDING**			
Funding	27	Describe sources of funding for the systematic review and other support (e.g., supply of data); role of funders for the systematic review.	16-17

From: Moher D, Liberati A, Tetzlaff J, Altman DG, The PRISMA Group (2009). Preferred Reporting Items for Systematic Reviews and Meta-Analyses: The PRISMA Statement. PLoS Med 6(7): e1000097. doi:10.1371/journal.pmed1000097

For more information, visit: **www.prisma-statement.org**.

For peer review only - http://bmjopen.bmj.com/site/about/guidelines.xhtml
